# Developmental and sexual divergence in the olfactory system of the marine insect *Clunio marinus*

**DOI:** 10.1038/s41598-020-59063-7

**Published:** 2020-02-07

**Authors:** Christine Missbach, Heiko Vogel, Bill S. Hansson, Ewald Große-Wilde, Andreas Vilcinskas, Tobias S. Kaiser

**Affiliations:** 1 Max Planck Institute for Chemical Ecology, Department of Evolutionary Neuroethology, Hans-Knoell-Strasse 8, D-07745 Jena, Germany; 2 Max Planck Institute for Chemical Ecology, Department of Entomology, Hans-Knoell-Strasse 8, D-07745 Jena, Germany; 3 Justus-Liebig University Giessen, Institute for Insect Biotechnology, Heinrich-Buff-Ring 26-32, D-35392 Gießen, Germany; 4 Center for Integrative Bioinformatics Vienna (CIBIV), Max F. Perutz Laboratories, University of Vienna, Medical University Vienna, Dr. Bohr Gasse 9, A-1030 Wien, Austria; 5 Max Planck Institute for Evolutionary Biology, Max Planck Research Group “Biological Clocks”, August-Thienemann-Strasse 2, 24306 Plön, Germany; 6 Present Address: Czech University of Life Sciences, Faculty of Forestry and Wood Sciences, EXTEMIT-K, Kamýcká 129, 165 00, Praha, Suchdol Czech Republic

**Keywords:** Chemical ecology, Genomics, Animal behaviour

## Abstract

An animal’s fitness strongly depends on successful feeding, avoidance of predators and reproduction. All of these behaviours commonly involve chemosensation. As a consequence, when species’ ecological niches and life histories differ, their chemosensory abilities need to be adapted accordingly. The intertidal insect *Clunio marinus* (Diptera: Chironomidae) has tuned its olfactory system to two highly divergent niches. The long-lived larvae forage in a marine environment. During the few hours of terrestrial adult life, males have to find the female pupae floating on the water surface, free the cryptic females from their pupal skin, copulate and carry the females to the oviposition sites. In order to explore the possibility for divergent olfactory adaptations within the same species, we investigated the chemosensory system of *C. marinus* larvae, adult males and adult females at the morphological and molecular level. The larvae have a well-developed olfactory system, but olfactory gene expression only partially overlaps with that of adults, likely reflecting their marine vs. terrestrial lifestyles. The olfactory system of the short-lived adults is simple, displaying no glomeruli in the antennal lobes. There is strong sexual dimorphism, the female olfactory system being particularly reduced in terms of number of antennal annuli and sensilla, olfactory brain centre size and gene expression. We found hints for a pheromone detection system in males, including large trichoid sensilla and expression of specific olfactory receptors and odorant binding proteins. Taken together, this makes *C. marinus* an excellent model to study within-species evolution and adaptation of chemosensory systems.

## Introduction

Many insect behaviours are heavily dependent on chemosensation, in particular on their sense of smell. This includes the location of food sources or oviposition sites, as well as intra- and interspecific communication^1^. Therefore, olfactory systems reflect the special needs of the respective insect. In this study we investigated the olfactory repertoire of the extreme specialist *Clunio marinus* Haliday, 1855 (Diptera, Chironomidae), a non-biting midge which inhabits the intertidal zone of the European Atlantic coast^2,3^. Being one of the very few marine insects, *C. marinus* displays a number of remarkable adaptations to this habitat.

First, the intertidal zone constantly changes between marine and terrestrial conditions. The life stages of *C. marinus* are divergently adapted to cope with these harsh changes: *C. marinus* spends most of its lifetime in the marine larval stage, which settle at the lower fringe of the intertidal where they are almost permanently submerged. Larvae live in tubes formed by their salivary gland secretions and sand, powdered shells, detritus or algae^4^. They most likely graze on algae or algal detritus. The terrestrial adult stage is restricted to a few hours, which are exclusively dedicated to reproduction^2,5^. We may thus speculate that the larval and adult olfactory systems are different, one being adjusted for foraging in an aquatic environment, the other for airborne sexual communication.

Second, the very short reproductive adult life is synchronized with the low waters of spring tide days just after full moon and new moon, when the tide reliably exposes the egg laying sites. This synchronization is achieved by a combination of circalunar and circadian clocks, which have made *C. marinus* a long-standing object of chrono-biological research^5,6^.

Finally, *C. marinus* adults are strikingly sexually dimorphic and display a curious mating behaviour^7,8^. The females have a very cryptic lifestyle: they are wingless and have only small legs, eyes and antennae. They are often unable to shed the pupal skin on their own. Female pupae float on the water surface until they are located by swarming males. Males patrol the water surface much like hovercrafts, with their legs resting on the water and their wings serving merely as propellers. The males free the females from the pupal skin in a stereotyped behaviour involving their large forceps-like hypopygium, the modified last abdominal segment. After a few seconds this directly ends in copulation. In that way males ensure to copulate with virgin females, likely enhancing their reproductive success. After mating, the males carry the females during a short nuptial flight and eventually deposit them on the larval substrates, which are exposed by the low tide and where oviposition takes place. Both sexes die in the rising tide. During the mating procedure, the males depend on the ability to localize the female, which could be triggered by visual, tactile, but also olfactory cues. Males are also involved in choosing the oviposition site. We may expect that the sexual dimorphism also extends to the olfactory system, with males having to perform a greater variety of olfactory tasks.

In order to investigate if the characteristic ecology and life-cycle of *C. marinus* are mirrored in the olfactory system at the species-level, but also within the species at the developmental and sex level, we used an array of morphological and genomic tools. The combination of scanning electron microscopy (SEM), immunohistochemistry and antennal backfilling, as well as analysis of the recently published *C. marinus* genome^6^ and RNAseq data allowed us to provide the first detailed description of a chironomid’s olfactory system, compare the olfactory system of *C. marinus* larvae, adult males and adult females and estimate the importance of chemoreception for the extreme specialist *C. marinus*.

## Results

### Morphology

#### Periphery: antennae and maxillary palps

**Figure 1:**
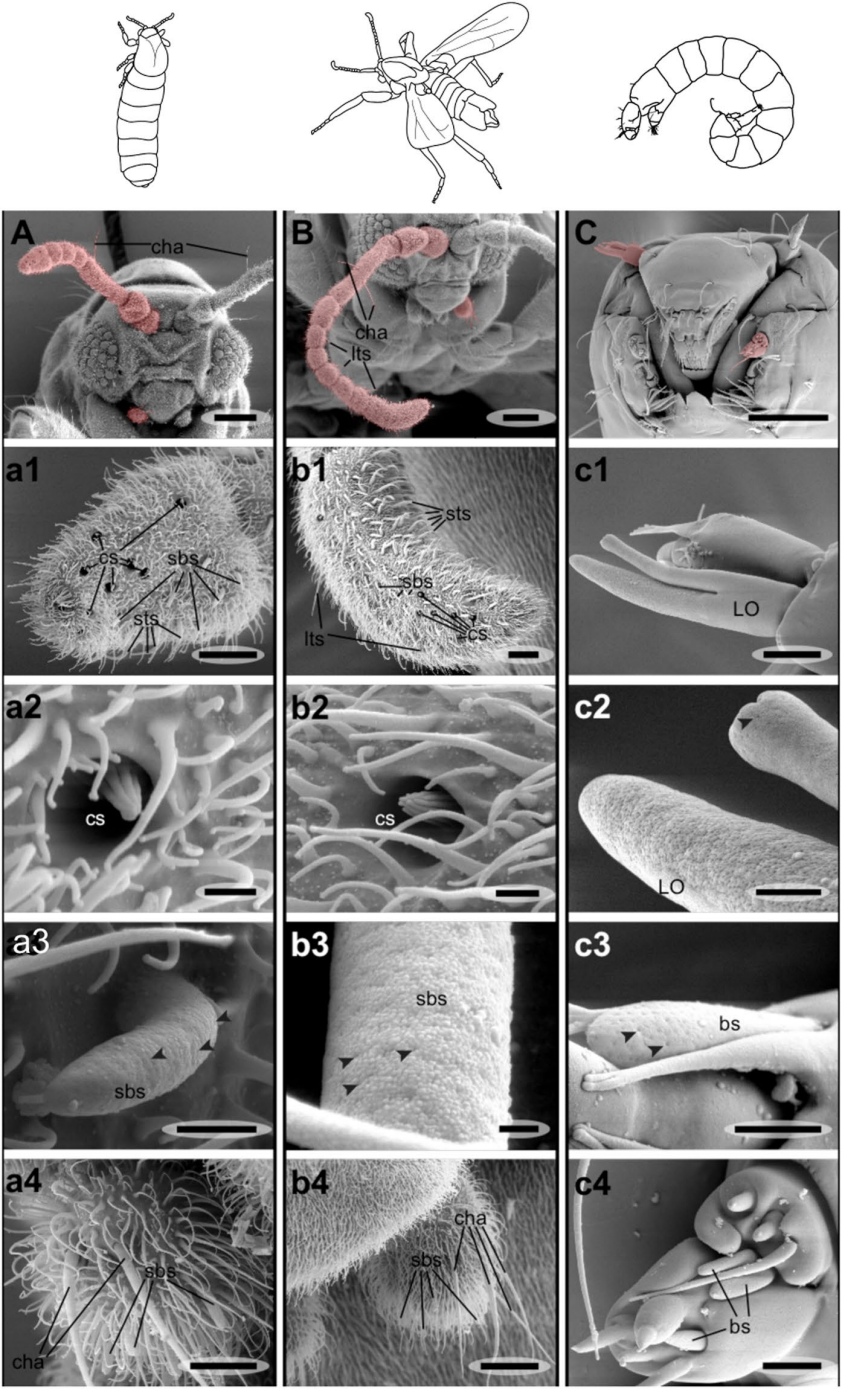
Scanning electron microscopic (SEM) pictures of olfactory organs of *C. marinus*. Antennae and the maxillary palps on the head of the adult female (**A**), adult male (**B**) and larva (**C**) are highlighted in red. The female antenna is shorter than the male antenna and consists of fewer antennal segments. Many putative olfactory sensilla can be found on the last antennal segment of antennae of the female (a1) and the male (b1). These include small trichoid sensilla (sts), as well as putative olfactory coeloconic sensilla (cs: a2, b2), large basiconic sensilla (lbs) and small basiconic sensilla (sbs: a3, b3). Large trichoid sensilla (lts) were only found on male antennae (**B**, b1). Small basiconic sensilla, together with mechanosensory chaetic sensilla (cha) were also found on the maxillary palps of both sexes (a4, b4). Larval antennae are short. The prominent Lauterborn organ (LO) has pores (c1, c2) and might fulfil a chemosensory function. Additionally a basiconic sensillum (bs) can be found (c3). The larval maxilla also bears multiporous basiconic sensilla (c4). Arrowheads in a3, b3, c2 and c3 point towards the pores in the surface of basiconic sensilla. *Scale bars:* A, B, C: 50 µm; a1, b1, c1, b4: 10 µm; a4, c4: 5 µm; c3: 2 µm; a2, a3, b2, c2: 1 µm; b3: 200 nm.

In order to see how the olfactory system of *C. marinus* differs between life stages and between the sexes, we investigated it at several levels. We started in the periphery by examining the main olfactory organs, the antennae and the maxillary palps. Several olfactory sensillum types have been described for dipterans, including basiconic, coeloconic, trichoid sensilla and sensory pits^9–12^. Especially large trichoid sensilla have been suggested to play a role in sex recognition and pheromone detection^13^. Antennae and maxillary palps of *C. marinus* larvae, adult males and females (Fig. 1A–C; red colouration) were screened for the presence of putative olfactory sensilla using scanning electron microscopy (SEM) and confocal laser scanning microscopy exploiting the autofluorescence of the insect cuticle.

**Figure 2:**
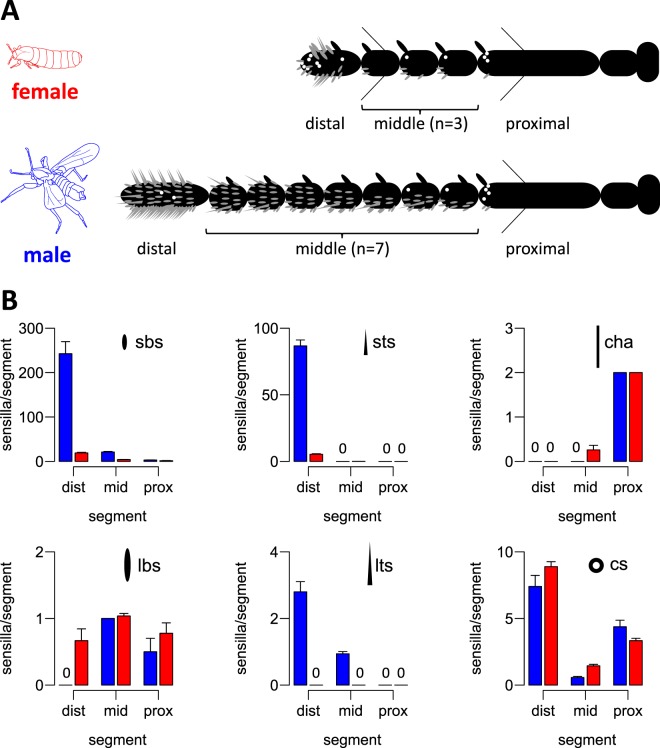
Antennal composition of *C. marinus* females and males. (**A**) Schemes of male and female antennae. The number of middle annuli differs between females (red) and males (blue). Typical sensilla content on each annulus is illustrated with pictograms as defined in panel (**B**). (**B**) Number of different sensilla types per annulus on distal (dist), middle (mid) and proximal (prox) annuli of *C. marinus* male (blue) and female (red) antennae. Numbers are given for small basiconic sensilla (sbs), large basiconic sensilla (lbs), small trichoid sensilla (sts), large trichoid sensilla (lts), chaetic sensilla (cha) and coeloconic sensilla (cs). Complete absence is indicated by a 0 in the plot.

Adults: A sexual dimorphism was already apparent in the antennal composition. Female antennae were composed of only 7 annuli (Figs. 1A, 2A), whereas male antennae had 11 annuli (Figs. 1B, 2A). This difference was sustained in sensillum numbers. Males had significantly more small basiconic sensilla (sbs) and small trichoid sensilla (sts) (Fig. 2B). As only one large basiconic sensillum (lbs) was found per annulus, overall the males also had more lbs due to the different number of annuli. For sbs and sts the highest abundance was found on the distal annulus (Fig. 2B, Suppl 1B,C), which in males was much bigger than in females (Figs. 1A,B, 2A). Interestingly, on this annulus the number of sensilla seemed to be not stereotyped: The bigger the proximal annulus, the higher the number of sensilla. Large trichoid sensilla (lts; Fig. 1b1) and mechanosensory chaetic sensilla (cha; Fig. 1A,B) were distinguished based on their different base structures. Cha arise from a round socket that allows movements of the sensilla (Suppl 2A), whereas the wall of lts is continuous with the antennal cuticle (Suppl 2B,C). SEM micrographs could not resolve a porous cuticular wall in *C. marinus* lts (Suppl 2b). However, lts were completely absent in females (Fig. 2B), suggesting they may play a role in sexual communication, as they do in *Drosophila*^13^. For all other sensillum types we found pores in the cuticle, supporting an olfactory function (Fig. 1a3,b3. Suppl. 2C,c). In contrast to the antennae, maxillary palps of adults showed no obvious sex differences (Fig. 1A,B,a4,b4). In both sexes the only putative palp olfactory sensilla were basiconic sensilla.

Larvae: The larvae do not show any apparent sexual dimorphism. Porous sensilla were detected on both the antennae and the palps (Fig. 1C). On the antennae these structures belong to the Lauterborn organ (LO) and an additional basiconic sensillum (Fig. 1c1,c3). Three putative olfactory basiconic sensilla were detected on the maxillary palp (Fig. 1c4, the sensillum pair is referred to as “bisensillum” in other chironomids^14^). Most other sensilla were sensillum styloconicum-like or poreless basiconic sensilla (referred to as “a and b setae”^14^).

**Figure 3:**
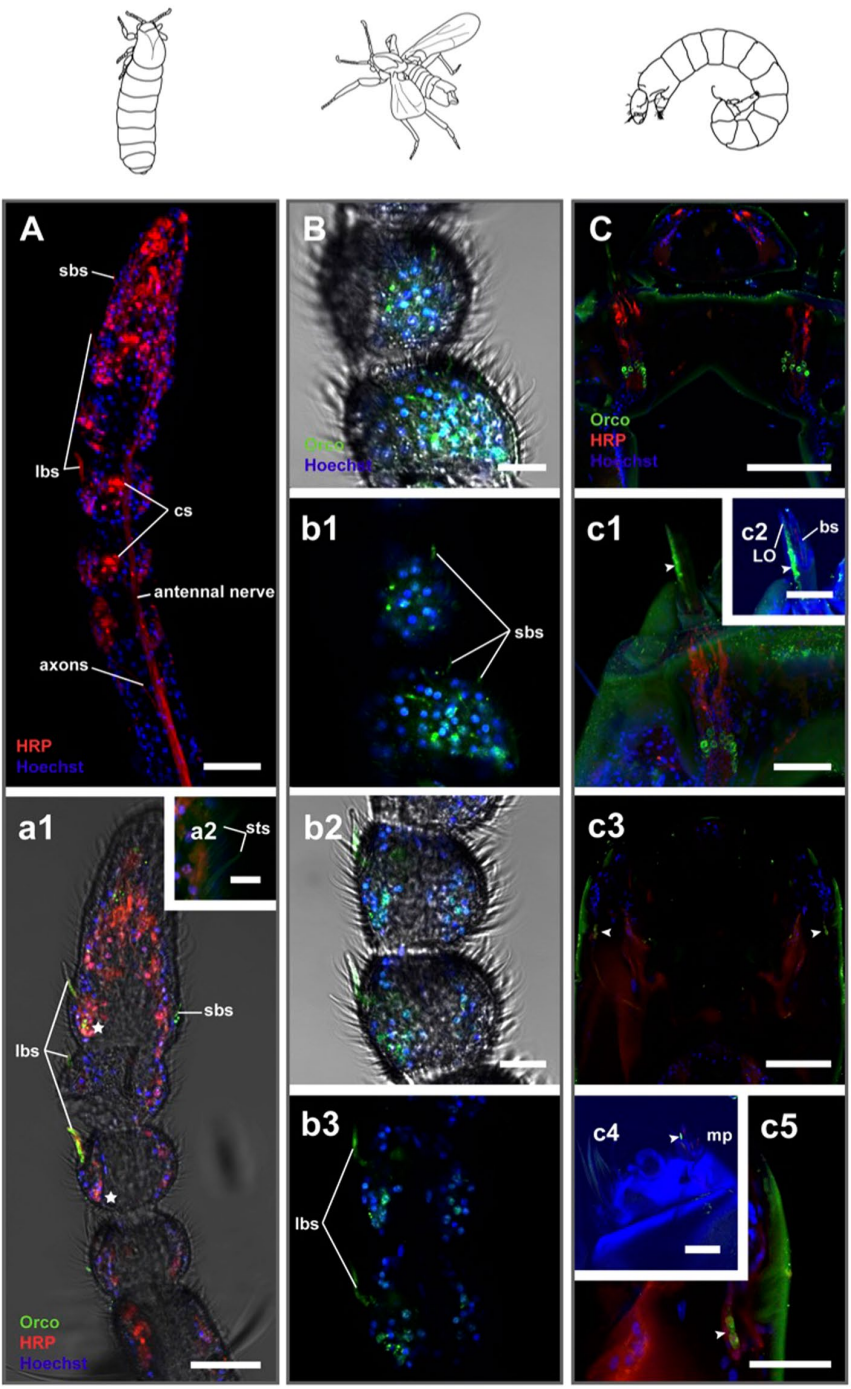
Olfactory sensory neurons (OSN) in the olfactory organs of *C. marinus*. OSN are visualized through Orco-immunoreactivity (Orco-ir) and confocal laser scanning microscopy in the antennae of the adult female (**A**) and adult male (**B**), as well as antennae and maxillary palps of the larva (**C**). (**A**) Total-projection of a female antenna labelled with anti-HRP as neuronal marker (red) and Hoechst for nuclei (blue). HRP-immunoreactivity (HRP-ir) was obtained in the antennal nerve, axons and dendrites of sensory neurons. High autofluorescence was found in the cuticle of coeloconic sensilla (cs). (a1) Single plane of the same confocal z-stack with Orco-ir added (green). Orco-ir was highest in the dendrites of the olfactory sensory neurons of small (sbs) and large basiconic sensilla (lbs). (a2) No HRP-ir and Orco-ir was obtained from small trichoid sensilla (sts). (**B**) Single confocal planes of a male antenna. As in females Orco-ir was found in the dendrites of small (**B**, b1) and large basiconic sensilla (b2, b3). (**C**) Single optical plane through the head of a larva. Both dendrites and OSN cell bodies innervating the larval antenna showed Orco-ir. (c1) Total projection of one hemisphere of the larval head. 17 Orco-ir cell bodies were counted per hemisphere. The dendrites of these neurons are localized in the Lauterborn organ (LO: arrow heads in c1, c2), likely an olfactory larval organ. (c2) Total projection with increased autofluorescence (blue) to visualize the antennal sensillum, which does not show Orco-ir. (c3, c5) Single optical plane through a larval head. Arrow heads point towards two additional Orco-ir OSN cell bodies per hemisphere. (c4) Dendrites of these neurons extend to a multiporous basiconic sensillum (bs; arrowhead) on the maxillary palps (mp). Increased autofluorescence (blue) highlights the outer cuticle of the maxillary palp and its sensilla. *Scale bars:* A, a1, c1, c2, c4, c5: 20 µm; a2: 5 µm; B, b2: 10 µm; C, c3: 50 µm.

Orco staining: To further investigate a putative olfactory function of antennal sensilla, we used an antibody designed against a conserved motif of the olfactory receptor coreceptor (Orco)^15^. Insect olfactory receptors (ORs) function as heteromultimers composed of a specific ligand binding OR and Orco^16,17^. In contrast to the ligand specific ORs, Orco is highly conserved across insects and is co-expressed in all OR-expressing olfactory sensory neurons (OSNs)^18^. We found Orco-immunoreactivity in OSNs in both male and female antennae (Fig. 3A,B). In adults the antibody labelling is best visible in the dendrites of large basiconic sensilla (Fig. 3a1,B,b1,b3). This is probably due to the fact that the large basiconic sensilla of *C. marinus* house a higher number of OSNs compared to small basiconic sensilla, as suggested by anti-HRP staining as a general neuronal marker (Fig. 3A,a1). OSN somata displayed weak labelling (Fig. 3a1,b1,b3). The adult maxillary palps did not show Orco-immunoreactivity. During preparation the palps were left attached on the head and this may have prevented diffusion of the antibodies into the palps. In the larvae dendrites and somata were Orco-immunoreactive (Fig. 3c). In larvae we were able to count the exact number of Orco-immunoreactive OSNs on antennae and palps: There are 17 cells on the antennae with dendrites extending into the Lauterborn organ (Fig. 3c,c1,c2) and two on the palps (Fig. 3c3,c5). The dendrites of the latter two neurons extended into the porous basiconic sensillum that is not belonging to the bisensillum (Fig. 3c4), suggesting the bisensillum does not serve olfactory functions.

#### Brain

Generally, insect OSNs that express the same set of chemoreceptors converge in the same region in the antennal lobe (AL) and form small spherical-like neuropils called glomeruli together with higher order neurons^19^. The number of glomeruli is therefore a good estimate for the number of receptors and for olfactory complexity^20^. We visualized the anatomy of the olfactory brain centres of *C. marinus* by using an antibody against presynaptic proteins (anti-synapsin) as general markers for neuropil structures^21^.

**Figure 4:**
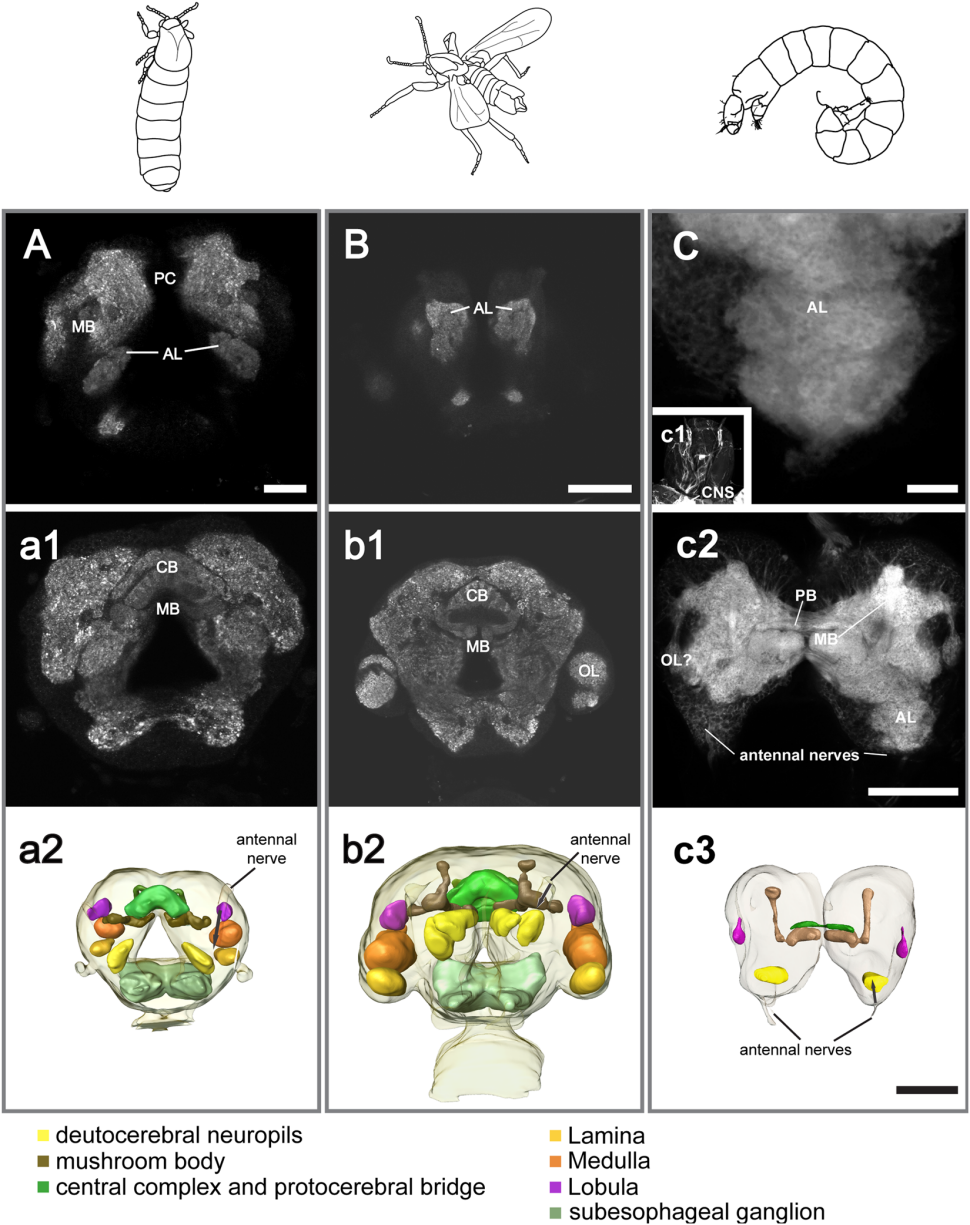
The brains of adult female, adult male and larvae of *C. marinus*. Synapsin-labelling in the brain of the adult female (**A**, a1), adult male (**B**, b1) and larva (**C**, c1, c2). The larval brain (CNS) is not situated within the head capsule (c1). The antennal lobes (AL) do not show glomerular organization in any of the life stages or sexes. Other neuropils including central body (CB) and protocerebral bridge (PB), mushroom bodies (MB), and optic neuropils (OL) are present in the protocerebrum, but in general less developed in females. A central body was absent in larvae, but a PB was found. (a2, b2, c3) Reconstructions of the female, male and larval brains respectively. The sizes of the brains are scaled to each other according to their actual size. *Scale bars:* A: 20 μm; B, c2, c3: 50 μm, C: 10 μm.

Adults: We were not able to identify a clear glomerular structure of the deutocerebral antennal lobes of *C. marinus*, neither in females (Fig. 4A) nor in males (Fig. 4B). This is much in contrast to other Diptera. In order to exclude technical limitations due to the size of the brains and the staining technique, we also performed antennal backfilling of male *C. marinus* (Suppl 3A,C). Again, we could not differentiate distinct glomeruli in the AL (Suppl 3C). So clearly, in *C. marinus* glomerular input regions are at least not as pronounced as in other insects. Male deutocerebral neuropils appeared bigger than those of the females. The mushroom body calices and lobes, which are brain centres associated with olfaction, are also comparatively small in *C. marinus* and especially in females (Fig. 4a1,a2,b1,b2). Furthermore, the antennal backfill highlighted strong contralateral connections of the antennal nerve (Suppl 3A,C). The terminals of these projections were restricted to small parts of the contralateral antennal lobe (Suppl 3A).

Larvae: As in the adults, a glomerular organization of the deutocerebrum was not visible in the larval brain (Fig. 4C,c1,c2).

### Chemosensory gene repertoire

Three receptor families are known to contribute to the insect chemical sense: olfactory receptors (ORs), gustatory receptors (GRs) and the variant ionotropic glutamate receptors (IRs). ORs and GRs belong to the arthropod chemoreceptor superfamily, which are ion channels that in structure look like seven-transmembrane-domain receptors with an inverted topology compared to G-protein coupled receptors^22,23^. To identify members of the three receptor families we made use of the recently published *C. marinus* genome^6^. Additionally, we generated RNAseq datasets of adult males, adult females and larvae to improve our gene models and to get a first impression of gene expression in the different life stages and sexes.

#### Olfactory receptors (ORs)

**Figure 5:**
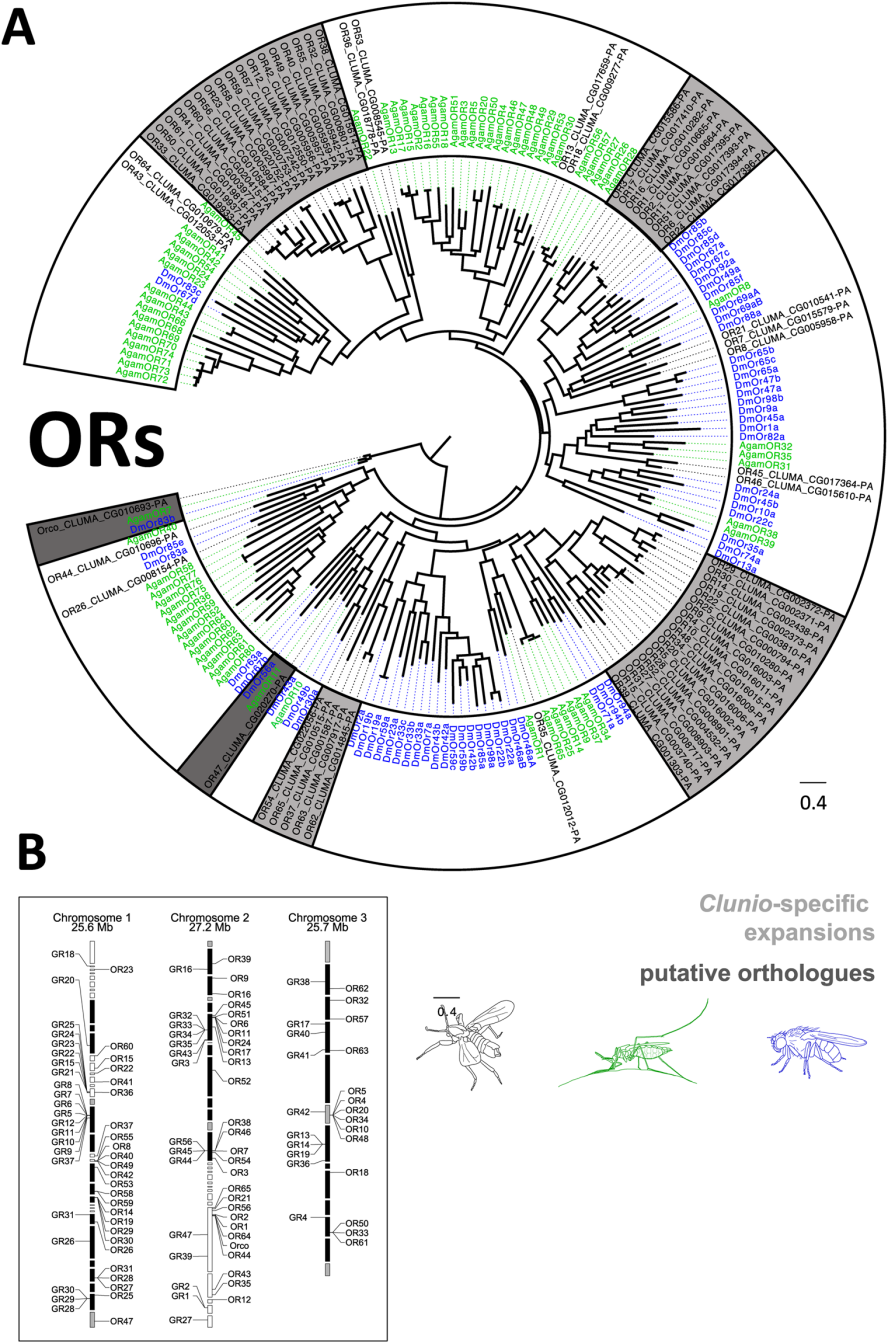
Olfactory receptor (OR) genes in *C. marinus*. (**A**) Dendrogram of *C. marinus* (black), *Anopheles gambiae* (green) and *Drosophila melanogaster* (blue) ORs, based on predicted amino acid sequences. *C. marinus* specific expansions are highlighted in light grey. Orthology relationships are highlighted in dark grey, but were only found for the coreceptor (Orco) and for DmelOR56a, AgamOR11 and the *C. marinus* OR47. (**B**) Genomic locations of OR and GR genes. Many of these genes are found in clusters.

While the olfactory receptor coreceptor (Orco)^24^ is highly conserved among insects, the ligand specific OR genes exhibit very little sequence similarity between insect orders, and even within the same insect order^18^. This likely reflects lineage- and species-specific chemosensory adaptations. In the *C. marinus* genome we annotated 66 OR genes (including the Orco), which is a similar number of ORs as in drosophilid flies. Phylogenetic analysis of *C. marinus* OR candidates along with the set of *Drosophila melanogaster* and *Anopheles gambiae* ORs revealed several lineage specific expansions with up to 19 ORs (Fig. 5A). Beside the Orcos of the different species, only AgamOr11, DmelOr56a and CmarOr47 formed a distinct cluster of orthologous receptors (Fig. 5A, dark grey shading). Across the genus *Drosophila* this particular OR is involved in the geosmin detection system, which senses the presence of harmful microbes^25^. It is possible that this important function is conserved in other dipetrans.

The identification of a similar number of ORs as compared to other dipterans was somewhat discrepant from the comparably simple morphology of the olfactory centres in the *C. marinus* brain. In order to get an idea of whether or not OR genes are expressed, we generated RNAseq data of heads and bodies of *C. marinus* adult males, adult females and larvae. In general, gene expression was very low for all receptors, independent of life stage, sex or tissue (Suppl 4A). Despite the limited data, there are several hints suggesting that the sexual dimorphism that was already found at the morphological level is also present at the level of gene expression: Orco expression was detected at about 6-fold higher levels in males compared to females (Suppl 4A). Many ORs do not have detectable expression, but the number of undetectable ORs is much larger in female heads (n = 49) compared to male heads (n = 23) (Suppl 4A). Finally, CmarOr45 is highly expressed specifically in adult male heads (Suppl 4A), which makes it a candidate for being a pheromone receptor.

Our RNAseq data also suggested the expression of various ORs outside the head of *C. marinus*, possibly being expressed in the legs or genitalia structures of adults or in some sensilla on the larval body. Our results do not rule out that more ORs are expressed at very low levels and an exact quantification will only be possible with additional data.

#### Gustatory receptors (GRs)

GRs belong to the arthropod chemoreceptor superfamily just as the ORs, but in contrast to ORs no universal coreceptor is known. Some GRs are known to bind volatile ligands such as CO_2_, but usually they bind compounds dissolved in aqueous solutions. Additionally, there are certain GR lineages that fulfil a conserved function, such as the sugar, fructose, bitter and CO_2_ receptor lineages^26–29^.

**Figure 6:**
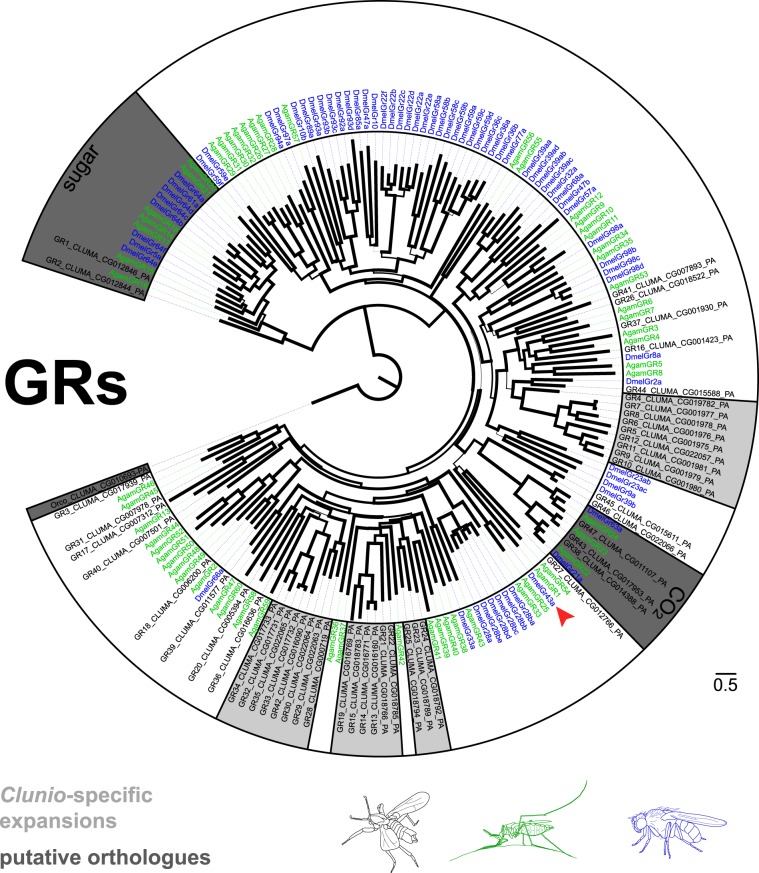
Gustatory receptor (GR) genes in *C. marinus*. Dendrogram of *C. marinus* (black), *Anopheles gambiae* (green) and *Drosophila melanogaster* (blue) GRs, based on predicted amino acid sequences. *C. marinus* specific expansions are highlighted in light grey. Conserved GRs are highlighted in dark grey, which includes the sugar and CO_2_ receptors. There is also an orthologue of the *D. melanogaster* bitter receptor DmelGr66a, but no fructose receptor (red arrow highlights DmelGR43a).

In the *C. marinus* genome we found 47 GR candidates (Fig. 6). We found direct orthologues for all three insect CO_2_ receptors and the bitter receptor DmelGr66a. In contrast, there is no orthologue to the *D. melanogaster* fructose receptor (DmelGR43a) and only two receptors belonging to the sugar receptor group were identified (CmarGR1 and CmarGR2). Expression of these sugar receptors seemed to be largely restricted to the larval stage (Suppl 4B), correlating with the fact that adults do not feed anymore. Similar to the ORs, GR expression levels were generally extremely low (Suppl 4B). The GR family also showed lineage specific expansions (Fig. 6). The genomic locations of both OR and GR genes indicate that these genes are frequently arranged in tandem arrays of up to eight closely related receptors (Fig. 5B). This supports the birth-and-death model previously suggested for chemosensory gene families^30^. The current data does not allow assessing the fraction of pseudogenes.

#### Variant ionotropic receptors (IRs)

The third insect chemoreceptor gene family is the IR family, ligand gated ion channels derived from ionotropic glutamate receptors (iGluRs)^31^. *D. melanogaster* IRs play a role in gustation^32–34^, salt detection^35^, thermosensation^36,37^ and hygrosensation^37^. A number of *D. melanogaster* IRs are involved in olfaction, namely members of the Ir75 clade, Ir41a, Ir92a, Ir84a, and Ir76a^38^. Their main ligands are small water soluble components such as amines and acids, which have an important role in many marine ecosystems^39^. Compared to ORs and GRs, antennal IRs show higher sequence conservation across species^33,40^.

**Figure 7:**
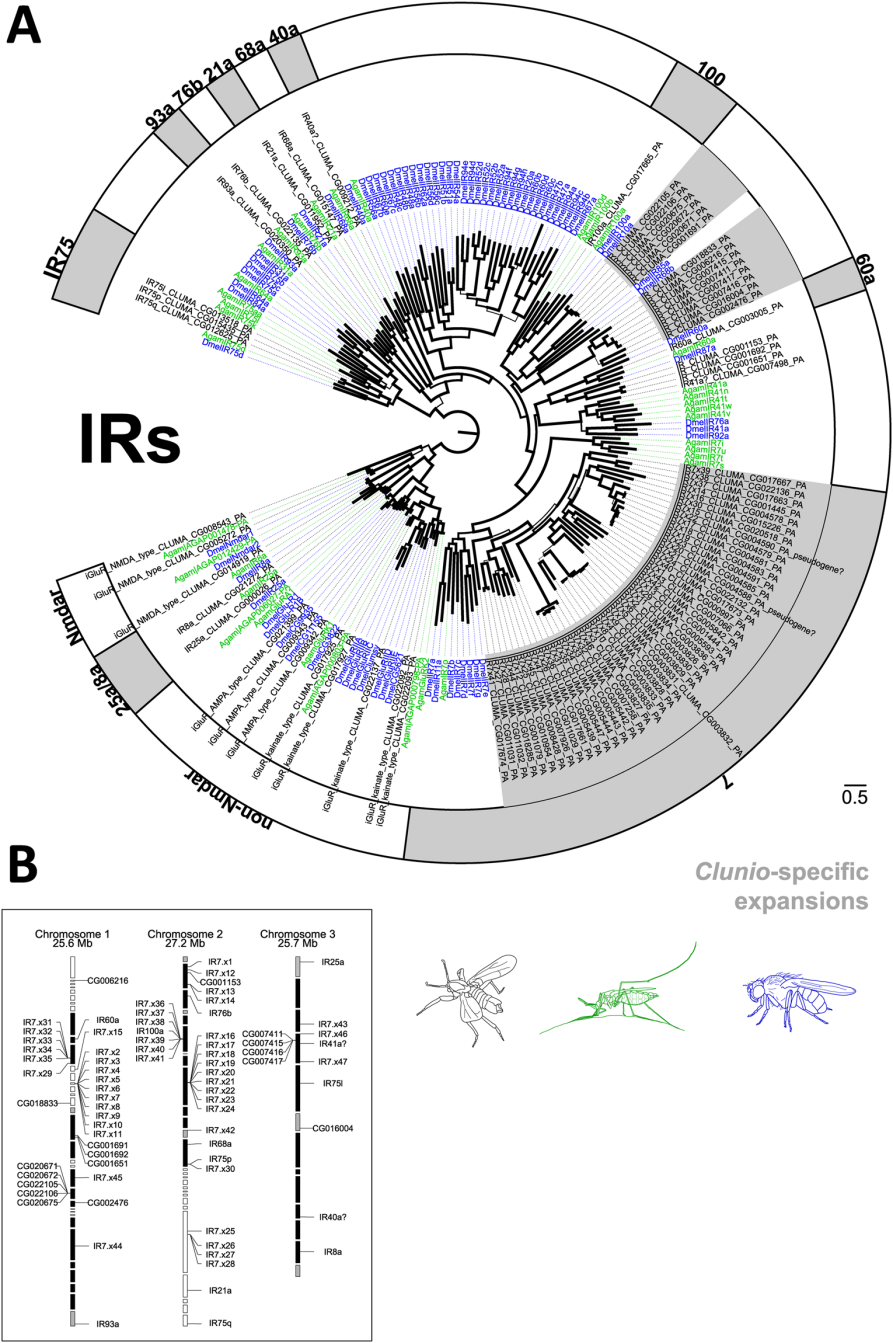
Variant ionotropic receptor (IR) genes in *C. marinus*. (**A**) Dendrogram of *C. marinus* (black), *Anopheles gambiae* (green) and *Drosophila melanogaster* (blue) IRs based on predicted amino acid sequences. *C. marinus* specific expansions are highlighted in light grey, orthology relationships are indicated in the outer ring by names. Clear one to one orthology relationships were found for IR25a, IR8a, IR93a, IR76b, IR21a, IR68a, IR40a, IR60a. An extraordinary big expansion of *C. marinus* IR genes was found in the IR7 clade. (**B**) Genomic location of *C. marinus* IR genes showing several tandem arrays of closely related IR7 genes, indicating several recent duplication events.

We have annotated 77 IR genes in the *C. marinus* genome (Fig. 7). These include direct orthologues to the *D. melanogaster* IRs involved in thermosensation (Ir21a, Ir25a, Ir93a), hygrosensation (IR25a, IR40a, IR93a) and salt detection (Ir76b) (Fig. 7A). There are also direct orthologues to IRs for which no specific elicitor is known (Ir8a, Ir60a, Ir68a), including one expansion with three gene copies in *C. marinus* (Ir87a). Regarding IRs involved in gustation in *D. melanogaster*, we found no orthologues of Ir11a or any member of the Ir20 clade, but one copy of Ir100a and a vast expansion of members of the Ir7 clade. There are 47 candidates belonging to the Ir7 clade in *C. marinus*, spread over several gene clusters in the genome (Fig. 7A,B).

As candidates for olfactory IRs, we found three members of the Ir75 clade and one member of the Ir41a/Ir76a/Ir92a clade, but no orthologue to Ir84a. Ir8a, Ir25a and Ir76b are considered olfactory IR co-receptors, which similar to the Orco for ORs form heteromultimers with other IRs, but do themselves not mediate specific odorant binding^41^. The orthologues of these three IR co-receptors show relatively high expression in *C. marinus* without obvious sex differences (Suppl 5). This is in line with the finding that coeloconic sensilla, which usually host most IR expressing neurons, did not differ in number between males and females.

#### Binding proteins (OBPs and CSPs)

**Figure 8:**
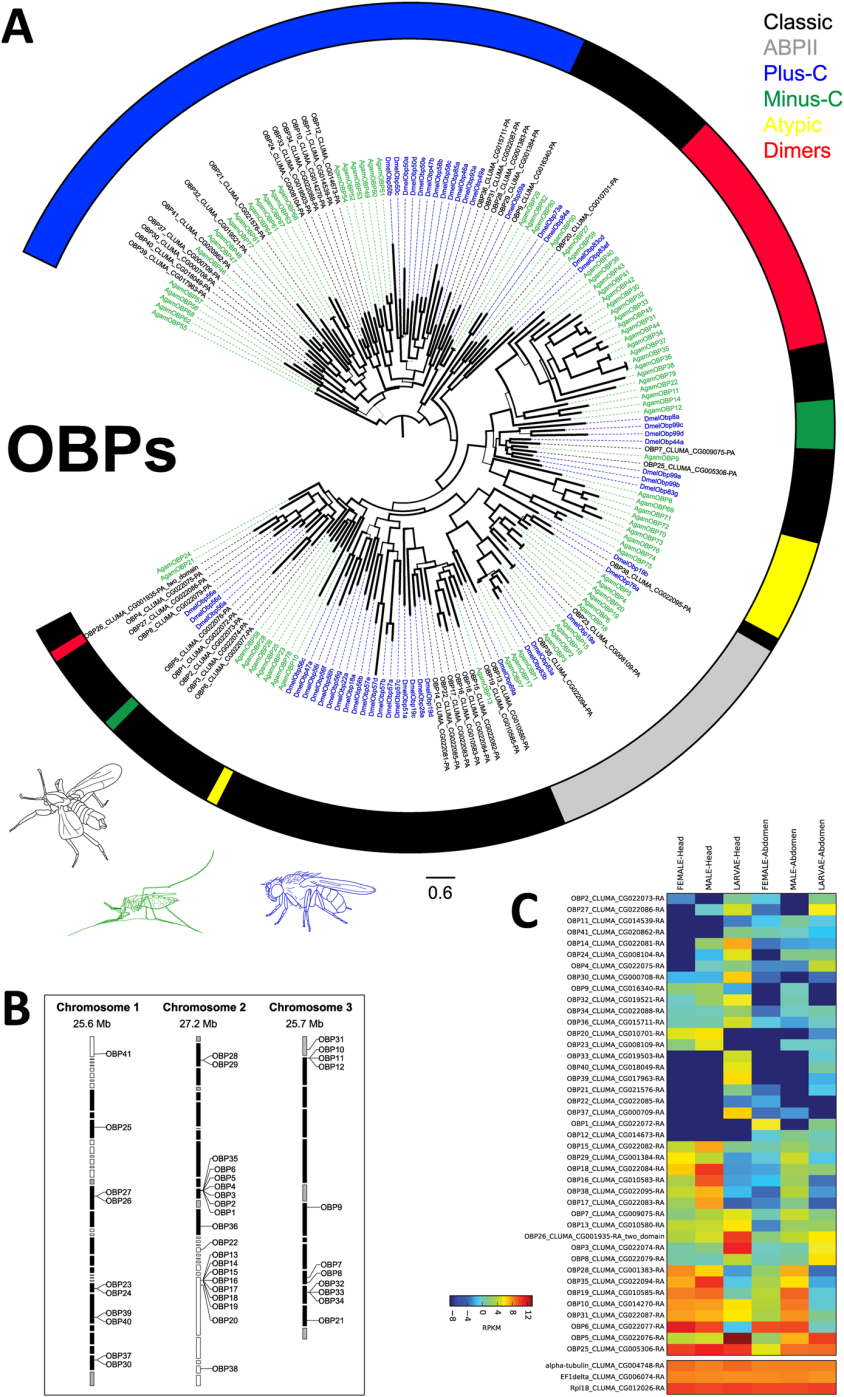
Odorant binding protein (OBP) genes in *C. marinus*. (**A**) Dendrogram of *C. marinus* (black), *Anopheles gambiae* (green) and *Drosophila melanogaster* (blue) OBPs based on predicted amino acid sequences. The colour code of the outer ring corresponds to specific types of OBPs (see legend in the graph). (**B**) Genomic location of *C. marinus* OBP genes. (**C**) Clustered heatmap of OBP expression in heads and abdomens of adult males, adult females and larvae. Compared to ORs, IRs and GRs, expression of many OBPs is much higher. There is a clear-cut difference in the OBP sets expressed in larval heads vs. adult heads.

In addition to the chemoreceptors, we annotated other gene families involved in olfaction or gustation: odorant binding proteins (OBPs), chemosensory proteins (CSPs) and sensory neuron membrane proteins (SNMPs). The OBP and CSP gene families code for proteins that reversibly bind small ligands, including odorants and tastants^42^. Both are expressed in olfactory and gustatory sensilla^43,44^, but are not restricted to chemosensory tissues^45–48^. In the *C. marinus* genome we annotated 41 OBP genes. Based on their cysteine patterns 17 *C. marinus* OBP candidates were assigned to the Plus-C OBPs, two to the Minus-C OBPs, one to OBP-dimers and three to the antennal binding proteins II (Fig. 8A). In contrast to Plus-C OBPs, *C. marinus* Minus-C and OBP-dimers likely evolved independently from those of *D. melanogaster* and *A. gambiae* (Fig. 8A), as was described for other species^49^. OBPs in general display very low overall sequence conservation^42^ and the only OBPs with clear orthology relationships across insects are Obp59a and Obp73a^50,51^. In *C. marinus* we found a putative orthologue of Obp59a (CmarObp9), but not Obp73a (Fig. 8A). We also found a putative orthologue of the *D. melanogaster* binding protein LUSH/DmelObp76a, which is CmarObp38 (Fig. 8A). LUSH has been shown to be essential for the detection of 11-*cis* vaccenyl acetate, one of the *D. melanogaster* pheromones^52^. In line with that CmarObp38 seems highest expressed in male heads (Fig. 8C). In general, OBPs were much higher expressed than receptors. Furthermore, most OBPs showed strong expression differences between larval and adult heads (Fig. 8C), suggesting that marine larvae and terrestrial adults may use different subsets of OBPs to bind waterborne vs. airborne substances.

With six CSP genes *C. marinus* has a similar number of CSPs as *A. gambiae* (8), but less than *C. quinquefasciatus* (21) or *Aedes aegypti* (18)^53,54^ (Suppl 6A). The conserved cysteine pattern, with 4 cysteines was found in all translated coding regions. CmarCSP3 and CmarCSP4 are much longer than previously described CSP proteins (300 and 616 AA), but there is no indication for misannotation and both are supported by transcript sequences. One of the *C. marinus* CSPs belonged to the 5-helical CSPs, whereas the other five grouped within the 6-helical CSPs (Suppl 6A). CmarCSP1 was strongly expressed in adult heads, whereas CmarCSP2 showed the highest expression in larval heads (Suppl 6B). This may suggest that as for OBPs different CSPs are used in marine larvae vs. terrestrial adults. CmarCSP6 was highly expressed in all tissues (Suppl 6B).

#### Sensory neuron membrane proteins (SNMPs)

SNMPs belong to a broad family of receptor proteins homologous to the human CD36 class of scavenger B1 receptor proteins, involved in signalling, transport, and internalization of lipid molecules^55,56^. In insects, SNMPs are characterized as olfactory receptor co-factors, which are required for normal response of OSNs particularly in pheromone detection^57,58^, but are not restricted to pheromone sensitive neurons^59^.

In *C. marinus* we found three SNMP genes, two belonging to the SNMP1 and one to the SNMP2 cluster (Suppl 6C). SNMP2 was found in two isoforms. CmarSNMP1-1 was highest expressed in male heads, but was also expressed in female heads and male body, containing legs and wings (Suppl 6D). CmarSNMP1-2 was expressed in both male and female heads, but barely in other tissues. CmarSNMP2 had a broad expression. Generally, our RNAseq data supported a role of SNMPs outside the head (Suppl 6D).

## Discussion

The life cycle of *C. marinus* is very special in several aspects: The larvae are truly “submarine”, which is very rare among insects, as most insects associated with marine habitats are living in bridging habitats like salt marshes^60^. This implies that the transition from larvae to adults is also a transition from marine to terrestrial habitats, on top of the transition from a feeding to a reproductive stage. In *C. marinus* the latter transition is particularly pronounced, as adults are extremely short-lived, and dedicate the few hours they live exclusively to reproduction. Finally, the adults are strongly sexually dimorphic, which likely facilitates successful reproduction. Because of these large differences between the tasks and ecological requirements of the life stages and sexes, we can expect that studying the olfactory system of *C. marinus* may reveal general patterns and mechanisms in the evolutionary specialisation of olfactory systems.

### Living in a marine environment

Adaptions to the marine habitat might be represented by expansions of certain gene families. As species-specific expansions in ORs and GRs are common, reflecting their birth-and-death mode of evolution, it is difficult to infer their ecological significance without functional testing. Still, the expansion of the IR7 gene family to 47 members in *C. marinus* is extraordinary. In other dipterans, members of this clade are expressed in larval and adult gustatory organs^33,61^. Thus, *C. marinus* IR7 receptors are the prime candidates for gustatory adaptation to the marine habitat.

In *C. marinus*, larval life lasts from 6 weeks to 1 year, depending on latitude. Adult life is reduced to a few hours. This divergence is reflected in the fact that adult olfactory centres are very simple compared to other dipterans, whereas the larval olfactory system seems normally developed. Larval antennae and palps are equipped with all common larval olfactory sense organs and show a relatively high number of Orco-positive neurons. In *A. gambiae* each larval OSN expresses the Orco and one specific OR, which are 12 in total^62^. In *C. marinus* we found 17 Orco-positive neurons in the antennae and two in the maxillary palps. We did not find a glomerular structure in the larval deutocerebrum, in line with findings in other chironomids^63^. However, in some midges synaptic markers, such as the synapsin antibody we employed, may not necessarily display glomerular structures even if present^64^.

Adults of *C. marinus* do not fly. They either hover on the water surface or crawl on the exposed algae and rocks, maintaining contact with water at all times. Thus, with the transition from marine larval life to adult life on the water surface, airborne chemosensory stimuli add to waterborne stimuli, but do not replace them. In line with that, there is a large overlap of expressed receptors between larvae and adults, suggesting that some olfactory tasks may be similar. For example, adults need to identify suitable algae for oviposition, and these algae are also the places where the larvae live and feed. In contrast, some CSPs and many OBPs seem to be expressed at different levels in larvae compared to adults, suggesting that adaptations to the detection of airborne vs. waterborne stimuli may rather happen at the level of odorant/tastant carriers, not necessarily at the level of receptors. This seems reasonable, as many substances may be transmitted both by water and air and can be detected with the same receptor, but need to be taken up from different media.

### Sexual dimorphism in the olfactory system

The difference in behavioural tasks performed by males and females is reflected at all levels of the olfactory system. Starting from the peripheral sensory organs, females are characterized by shorter antennae, with a smaller number of most sensillum types, as well as the complete absence of large trichoid sensilla. The reduction in sensillum types is reflected in lower Orco expression in female heads. Finally, olfactory brain centres are smaller in females.

Males probably perform more complex olfactory tasks, as they need to locate the females and navigate to the oviposition sites during the nuptial flight. The presence of large trichoid sensilla, highly expressed SNMPs and LUSH, three important elements of pheromone detection systems, suggest the presence of pheromones in *C. marinus*. As females are basically immobile and often cannot shed the pupal skin without male assistance, the most likely scenario is that females emit a pheromone to make sure that they are located by males. Signals could be directly emitted by the females or could be water-insoluble compounds evaporating from the pupal skin, when the pupae reach the water surface. Future investigations on the presence of a *C. marinus* pheromone will help to understand, if the sex-specific differences in the olfactory system are only due to the different behavioural tasks or also due to the presence of sex-specific pheromones.

The very passive role of females in the mating behaviour makes female choice unlikely. But once females are deposited on the algal substrates they seem to choose the exact oviposition site. This choice may involve the detection of algal odours or of odours emitted by eggs of conspecifics or larval faeces, as was described in other nematocera^65^. ORs expressed in the female abdomen may play a role in egg laying behaviour.

### Morphology vs. molecular biology

At first sight, it seems contradictory that the antennal lobe (AL) of *C. marinus* shows no glomeruli, which would suggest a very simple olfactory system, but at the same time a normal number of sensilla and olfactory receptors were found. But interestingly, aglomerular ALs have been described for other insects that spent most of their life as aquatic larvae and have only a short adult life-span, e.g. the chironomid *Chironomus dolichotomus*, Ephemeroptera, Odonata, Plecoptera and some Trichoptera^63,66^. In contrast, dipterans with an aquatic development but long adult lifespan possess a glomerular antennal lobe (e.g. *A. gambiae*, *A. aegypti*). Possibly, the typical glomerular organization is only necessary when a long adult life requires signal modulation and integration in the course of learning. *C. marinus* adults live only for a few hours and spend this time exclusively for reproduction. Their reproductive behaviours are highly stereotyped^8^, making it possible that the respective olfactory tasks consist mostly of hard-wired innate responses that do not require learning. This may also be reflected in the small size of the mushroom bodies, especially in females. Furthermore, a lack of a glomerular AL is often correlated with a pronounced reduction or even loss of the mushroom body calyx^67^, as we also found in *C. marinus*.

Finally, a relatively high number of OR genes seems to be expressed in tissues other than the head, especially in the larval abdomen. This may be explained by the presence of olfactory sensilla in the abdomen or by a possible co-option of ORs into non-olfactory functions. Both scenarios would be worth further investigation.

## Conclusions

Our detailed characterization of the *C. marinus* olfactory system underlines that not only species, but also different life stages and sexes of the same species, are equipped with sensory systems that are tuned to their specific needs: In *C. marinus*, the long-lived larvae show a normally developed olfactory system, whereas in the short-lived adults the olfactory system is much simpler. Differences in gene expression between the life stages may reflect their marine vs. terrestrial life-style. The olfactory systems of males and females are strikingly sexually dimorphic, much in line with the highly specific tasks they have to fulfil during the stereotyped mating behaviour. Taken together, this suggests that *C. marinus* is an excellent model for further studies on the evolution and adaptation of olfactory systems.

## Methods

### Animal rearing

All experiments were performed on individuals from the *Jean* laboratory strain of *Clunio marinus*, which was also used to establish the *C. marinus* reference genome. The laboratory stock was bred according to Neumann^2^. Briefly, animals were kept in 20 × 20 × 6 cm plastic containers with sand and natural seawater diluted to 15‰ with desalted water. They were fed diatoms (*Phaeodactylum tricornutum*, strain UTEX 646) in early larval stages and nettle powder in later stages. Temperature in the climate chambers was set to 20 °C and the light dark cycle (LD) was 16:8 hours. All animals were subject to an artificial moonlight cycle with an incandescent flashlight bulb (about 1 Lux) switched on all night for four successive nights every 30 days. Because of the synchronized development, adults were always available for sampling at the same time of the artificial moonlight cycle (days 12 to 15) and of the LD cycles (zeitgeber time 19 to 22). Larvae were sampled at different times of the artificial moonlight cycles, depending on the experimental requirements.

### Scanning electron microscopy (SEM)

For investigation of peripheral olfactory structures we used female, male and larval *C. marinus* preserved in 70% ethanol. Tissue was dehydrated in an ascending ethanol series (80%, 90% 96%, 3 × 100% ethanol, 10 min each), critical point dried (BAL-TEC CPD 030, Bal-Tec Union Ltd., Liechtenstein), mounted on aluminium stubs with adhesive tape and sputter coated with gold on a BAL-TEC SCD005 (Bal-Tec, Liechtenstein). Specimens were examined in a LEO 1530 Gemini scanning electron microscope (Zeiss, Germany) set at 10 kV and 11 to 16 mm working distance.

### Autofluorescence for sensillum count

The sensillum numbers of 8 male and 8 female antennae were counted using the high cuticular autofluorescence at 488 nm excitation. Heads or single antennae of ethanol fixed material were washed several times in 0.1 M phosphate-buffered saline (PBS, pH 7.4, Sigma Aldrich, USA) and mounted in 50% glycerol in PBS between two cover slides. For visualization samples were scanned using confocal laser scanning microscopy (LSM 880, Zeiss, Germany). Cuticle was exited using the 488 nm line of the microscope’s argon laser. All pictures were taken using a 40 × W objective (C-Apochromat 40x/1.20 W Korr M27). Scanning resolution was set to 1024 × 1024 pixel. Because of the small size of the antennae it was possible in most of the cases to scan through the complete antennae and visualize both front and back side. In all other cases the object was inverted and rescanned. By using landmarks such as big sensilla it was possible to combine both data sets for counting.

### Brain morphology

Male, female and larval *C. marinus* were fixed in 4% paraformaldehyde (ROTH, Germany) in 0.1 M PBS overnight at 4 °C. Nervous system was prepared in 0.1 M PBS containing 0.001% Trition X (PBST). Subsequently, samples were washed six times for 30 min in 1 × PBST and thereafter blocked for 3 h in 2% normal goat serum (NGS) in 1xPBST. To label synaptic regions the monoclonal mouse anti-synapsin antibody (anti-SYNORF-1 – 3C11, Developmental Studies Hybridoma Bank, USA) was applied at a 1:500 dilution in 2% NGS-PBST and incubated for 4 days at 4 °C. Detection of the 3C11 antibody was performed by incubating in a goat-anti mouse antibody linked to Alexa Fluor 546 at a dilution of 1:200 (Invitrogen, USA) for 3 days at 4 °C. The incubation was followed by washing steps as described above. For visualization, samples were mounted in 50% glycerol on a microscope slide and scanned using confocal laser scanning microscopy (LSM 510 Meta or LSM 880, Zeiss, Germany). Alexa 546 was exited using the Helium Argon 543 laser. Signals were detected by a spectral detector (555–681 nm). All pictures were taken using a 40 × W objective (C-Apochromat 40x/1.2 W UV-VIS-NIR or C-Apochromat 40x/1.20 W Korr M27). Scanning resolution was set to 1024 × 1024 pixel. 3D reconstructions were prepared with Amira 4.1.1 (Mercury Systems, Germany).

### Olfactory receptor coreceptor (Orco) immunohistochemistry

Antennae and whole heads of fixed male, female and larval *C. marinus* were treated as described in the part brain morphology. The primary antibody against Orco (originally termed anti-R2, kindly provided by Prof. Jürgen Krieger, University of Halle-Wittenberg, Germany) was raised against a 17 amino acid peptide (NH2-NQSNSHPLFTESDARYH-COOH) that is fully conserved in the *Bombyx mori*, *Heliothis virescens* and *Manduca sexta Orcos*^15,68^. This peptide is also highly conserved in the *C. marinus* Orco, with only two conservative amino acid replacements (NH2-NQSNSHPLFTESNSRYH-COOH). A BLAST search of *M. sexta* Orco against all *C. marinus* proteins shows that the two species’ Orcos have 65% amino acid identity, while the second best blast hit (*Cmar*OR1) only has 27% identity. In a BLAST search of the 17 amino acid peptide against all *C. marinus* proteins only Orco had a significant BLAST hit. We therefore do not expect the antibody to cross react with antigens other than Orco. The anti-Orco antibody was applied at a 1:500 dilution in 2% NGS-PBST and incubated for 4 days at 4 °C. Detection of the Orco antibody was performed by incubating in a goat-anti rabbit antibody linked to Alexa Fluor 488 at a dilution of 1:200 (Invitrogen, USA) for 3 days at 4 °C. In addition, we added goat anti-horseradish peroxidase (anti-HRP) antibodies conjugated to Cy3 (Jackson Immuno Research, USA) at a dilution of 1:50 to visualize neuronal tissue and Hoechst33342 (ThermoFisher Scientific, Germany) as nuclear marker (1:2000). For visualization, samples were mounted in 50% glycerol on a microscope slide and scanned using confocal laser scanning microscopy (LSM 880, Zeiss, Germany). Alexa Fluor 488 was exited using the 488 nm line of the microscope’s argon laser, while a Helium Argon 543 laser was used to activate Cy3. Signals were detected by a spectral detector (quasar: 490–553 nm and 555–681 nm). Hoechst was excited by a laser diode at 405 nm. Pictures were taken using a 40 × W (C-Apochromat 40x/1.20 W Korr M27) or 63 × W objective (NCI PlanNeofluar 63x/1.3 Dic Imm Korr). Scanning resolution was set to 1024 × 1024 pixel.

### Antennal nerve backfill

For several living males one antenna was cut carefully without destroying the cuticle of other body parts. The animals were placed in 1xPBS (without detergent) and carefully centrifuged until the animals were submerged. The animals were incubated in a micro ruby solution (Tetramethylrhodamin and biotin; ThermoFisher Scientific, Germany) for two hours at room temperature and immediately fixed in 4% PFA in 0.1 M PBS overnight at 4 °C. The brains were dissected in ice cold 0.1 M PBS and washed several times in PBST for about an hour. The dextran of the microruby was detected with Streptavidin conjugated with Alexa Fluor 488 (Invitrogen) by incubation in the fridge for one day. Afterwards the brains were washed several times in PBST, mounted in Mowiol (Sigma-Aldrich, Germany) and scanned using the 40x(C-Apochromat 40x/1.2 W UV-VIS-NIR) or 63x objective (C-Apochromat 63x/1.2 W corr) of the LSM 510 Meta (Zeiss, Germany).

### Image processing

Contrast and false colour images were optimized in Zen 2011 (Zeiss, Germany). Further image processing, including cutting and image mode conversion was done in Adobe Photoshop® CS4, figures were prepared in Adobe Illustrator® CS4 (Adobe Systems Software, Ireland).

### Annotation of olfactory genes

Annotation of olfactory genes was based on the CLUMA1.0 reference genome. For all examined gene families (ORs, GRs, IRs, OBPs, CSPs, SNMPs) the respective set of *D. melanogaster* genes served as a BLAST query to retrieve *C. marinus* homologs from the CLUMA1.0 gene models (e-value cut-off e^−2^). Then putative *C. marinus* homologs were blasted against NCBI’s nr database and only retained if the best hit in another species was a member of the gene family under investigation. In a second step, all identified *C. marinus* homologs served as BLAST queries against the CLUMA1.0 gene models to obtain additional homologs, which were also checked for being members of the respective gene family as described above. Finally, all gene models were manually curated and improved by comparison to the available transcript data and protein sequences from other insects, as were used for automated annotation of the *C. marinus* reference genome^6^. ORs, GRs and IRs were further checked for the presence of a 7tm domain, OBPs and CSP for their characteristic cysteine residue patterns. A few gene models are partial. The resulting AA sequences are provided in the supplement and the corrected gene models will be published with the second release of the *C. marinus* reference genome (manuscript in preparation).

Alignments with other dipteran members of the respective gene families were carried out using the MAFFT plugin in Geneious Pro 5.0.4 (E-INS-I parameter set; MAFFT 7.453 for the OR tree)^69,70^. Dendrograms were calculated using maximum likelihood analysis with FastTree2^71,72^ and displayed and edited with FigTree (http://tree.bio.ed.ac.uk/software/figtree). Candidates were named with the abbreviation for the gene family and ascending numbers with the exception of receptors were a clear homology could be assigned.

### RNA isolation

RNA was extracted from larval, male and female heads and abdomen. Each of the total of six samples consisted of at least 20 pooled individuals. Total RNA was isolated from sample material homogenized in TriReagent (Molecular Research Centre, Cincinnati, OH, USA) using ball bearings and a TissueLyzer (Qiagen, Hilden, Germany) and subsequently purified using the DirectZol kit (Zymo Research) following the manufacturers’ guidelines. The integrity of the RNA was verified using an Agilent 2100 Bioanalyzer and a RNA 6000 Nano Kit (Agilent Technologies, Palo Alto, CA). The quantity of RNA was determined using a Nanodrop ND-1000 spectrophotometer. Since the goal of the experiment was not to identify differences between individuals, but rather to obtain a robust tissue-specific gene expression map, we have opted for a pooling strategy, combining large numbers of individuals per sample to massively reduce biological variability and the impact of individual outliers on observed gene expression differences.

### RNA-Seq and expression analysis

RNA-Seq was carried out for 6 different RNA samples using poly(A)^+^ enriched RNA fragmented to an average of 150 nucleotides. Sequencing was carried out by the Max Planck Genome Centre Cologne (MPGCC) on an Illumina HiSeq 2500 Genome Analyzer platform using single read (1 × 100 bp) technology. This yielded approximately 25 million reads for each of the 6 samples. Quality control measures, including the filtering of high-quality reads based on fastq file scores, the removal of reads containing primer/adapter sequences, and trimming of the read length, were carried out using CLC Genomics Workbench v9.1 (http://www.clcbio.com). The same software was used for *de novo* transcriptome assembly, combining all 6 RNA-Seq samples, and selecting the presumed optimal consensus transcriptome as previously described^73^. The final *de novo* reference transcriptome assembly of *C. marinus* contained 27,239 contigs (minimum contig size = 200 bp) with a maximum contig length of 17,430 bp. The transcriptome was annotated using BLAST, Gene Ontology and InterProScan searches implemented in BLAST2GO PRO v4.1 (www.blast2go.de)^74^ and used to verify some of the olfactory genes based on the CLUMA1.0 reference genome.

Digital gene expression analysis was carried out using CLC Genomics workbench to generate BAM mapping files, QSeq (DNAStar Inc.) to remap the Illumina reads from all 6 samples onto the CLUMA1.0 reference transcripts (with manually curated and corrected olfactory gene sequences), and finally by counting the mapped sequence reads to estimate the expression levels, using previously described parameters for read mapping and normalization (for a detailed description of the procedure see^73,75^).

The short read data have been deposited in the EBI short read archive (SRA) with the following sample accession numbers: ERS2954175-ERS2954186. The complete study can also be accessed directly using the following URL: http://www.ebi.ac.uk/ena/data/view/ PRJEB30068.

### Ethics approval

There are no regulations on work with *C. marinus* and thus no ethics approval was required. Nevertheless, we have minimized the use of animals to the numbers that were absolutely required.

## Supplementary information

Supplementary Information.

Supplementary Dataset 7.

## Data Availability

As NCBI or ENA do not accept revised gene models, all protein sequences of olfactory genes as used in and revised for this study are given as Supplement 7. They will be included in the next release of the *C. marinus* reference genome. The short read data have been deposited in the EBI short read archive (SRA) under accession numbers ERS2954175-ERS2954186, project number PRJEB30068.
