# Supplementary material for: Developmental and sexual divergence in the olfactory system of the marine insect Clunio marinus
Source: Sci Rep. 2020 Feb 7;10:2125. doi: 10.1038/s41598-020-59063-7 (PMC7005812; doi:10.1038/s41598-020-59063-7)

Supplementary Material

Developmental and sexual divergence in the olfactory system of the marine insect *Clunio marinus*

Christine Missbach[1*], Heiko Vogel[2], Bill S. Hansson[1], Ewald Große-Wilde[1,6], Andreas Vilcinskas[3], Tobias S. Kaiser[4,5*]

[1] Max Planck Institute for Chemical Ecology, Department of Evolutionary Neuroethology, Hans-Knoell-Strasse 8, D-07745 Jena, Germany
[2] Max Planck Institute for Chemical Ecology, Department of Entomology, Hans-Knoell-Strasse 8, D-07745 Jena, Germany
[3] Justus-Liebig University Giessen, Institute for Insect Biotechnology, Heinrich-Buff-Ring 26-32, D-35392 Gießen, Germany
[4] Center for Integrative Bioinformatics Vienna (CIBIV), Max F. Perutz Laboratories, University of Vienna, Medical University Vienna, Dr. Bohr Gasse 9, A-1030 Wien, Austria
[5] Max Planck Institute for Evolutionary Biology, Max Planck Research Group "Biological Clocks", August-Thienemann-Strasse 2, 24306 Plön, Germany
[6] Current address: Czech University of Life Sciences, Faculty of Forestry and Wood Sciences, EXTEMIT-K, Kamýcká 129, 165 00 Praha-Suchdol

Supplementary Figure 1 Comparison of sensillum numbers between eight female and eight male antennae.

(A) Pictures in boxes showing single optical planes of the antennae (left boxes) or a total projection of the distal antennal segments (right side). These illustrate how the different sensilla can be distinguished in the cLSM stacks (cs – coeloconic sensillum, cha – chaetic sensillum, lbs – large basiconic sensillum, sbs – small basiconic sensillum, lts – large trichoid sensillum, sts – small trichoid sensillum). Males (blue) and females (magenta) differ significantly in the numbers of small (p =0.0009, Mann-Whitney-U test) and large basiconic sensilla (p = 0.0006), as well as small (p = 0.0009) and large trichoid sensilla (p = 0.0004). In all cases the males had more sensilla than the females. Large trichoid sensilla were exclusively found on male antennae. The pictograms for each sensilla type are re-used in the other panels. **(B)** Antennal drawing and distribution of sensilla for a female *C. marinus*. (**C)**: Antennal drawing and distribution of sensilla for a male *C. marinus*.

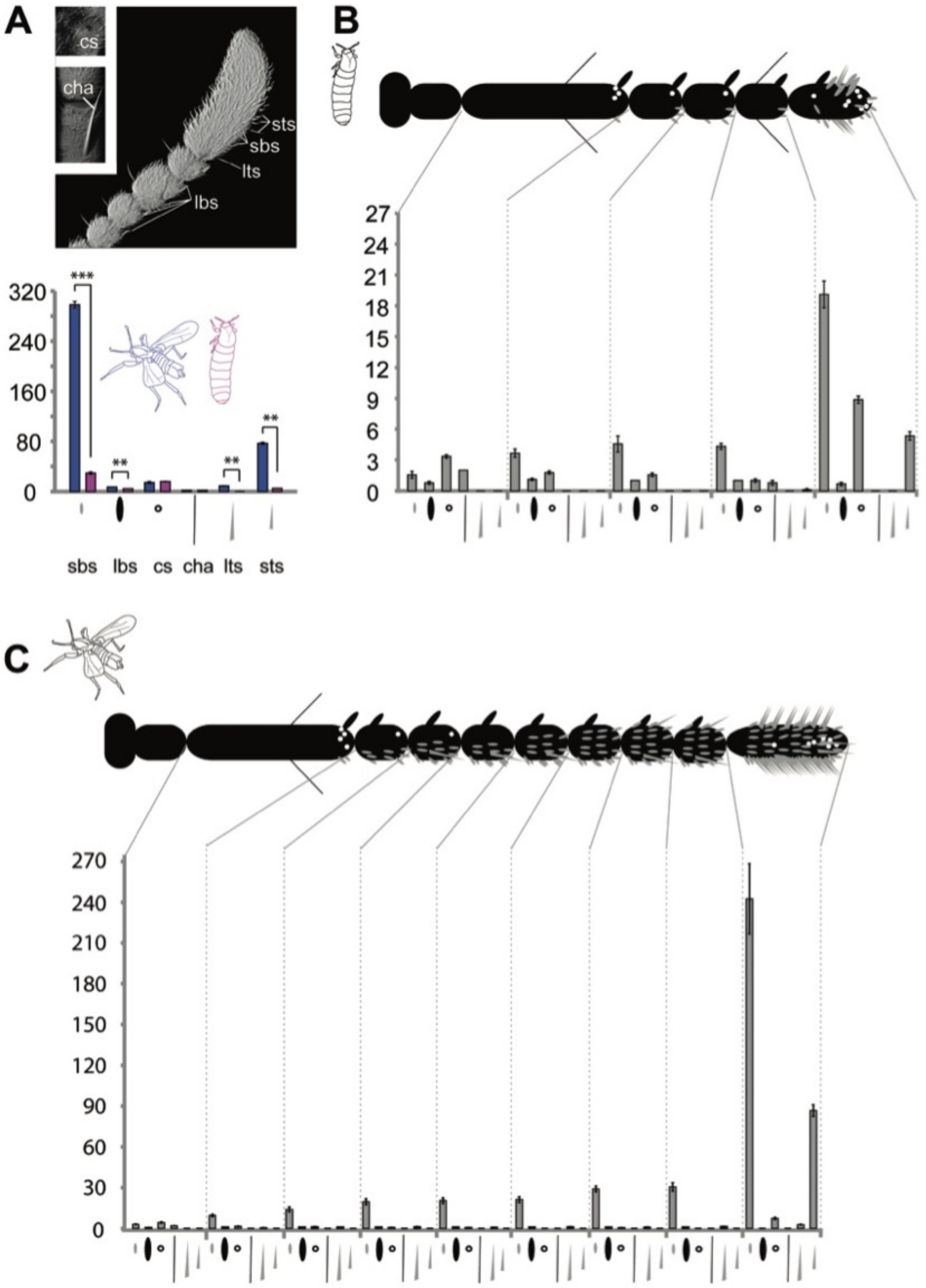

Supplementary Figure 2 Scanning electron microscopic (SEM) pictures of different sensillum types.

(A) Chaetic sensillum. **(B)** Large trichoid sensillum (lts). **(C)** Small trichoid sensillum (sts). The different sensillum types were differentiated based on the base and their size. Chaetic sensilla articulate in a flexible socket (arrow head in **A**), whereas the cuticle in the socket region was continuous for small and trichoid sensilla (arrow heads in **B** and **C**). Their presence in male or female *C. marinus* is indicated by the small pictograms. The small boxes display details of the shaft structure of the corresponding sensillum (**a**, **b**, **c**). Arrowheads in **c** point towards possible pores in the cuticular surface of a small trichoid sensillum. No obvious pores were found in the surface of chaetic and large trichoid sensilla, but the presence of pores cannot be excluded. *Scale bars:* **A**, **B**: 2µm; **C**: 1µm; **a**, **b**, **c**: 250nm.

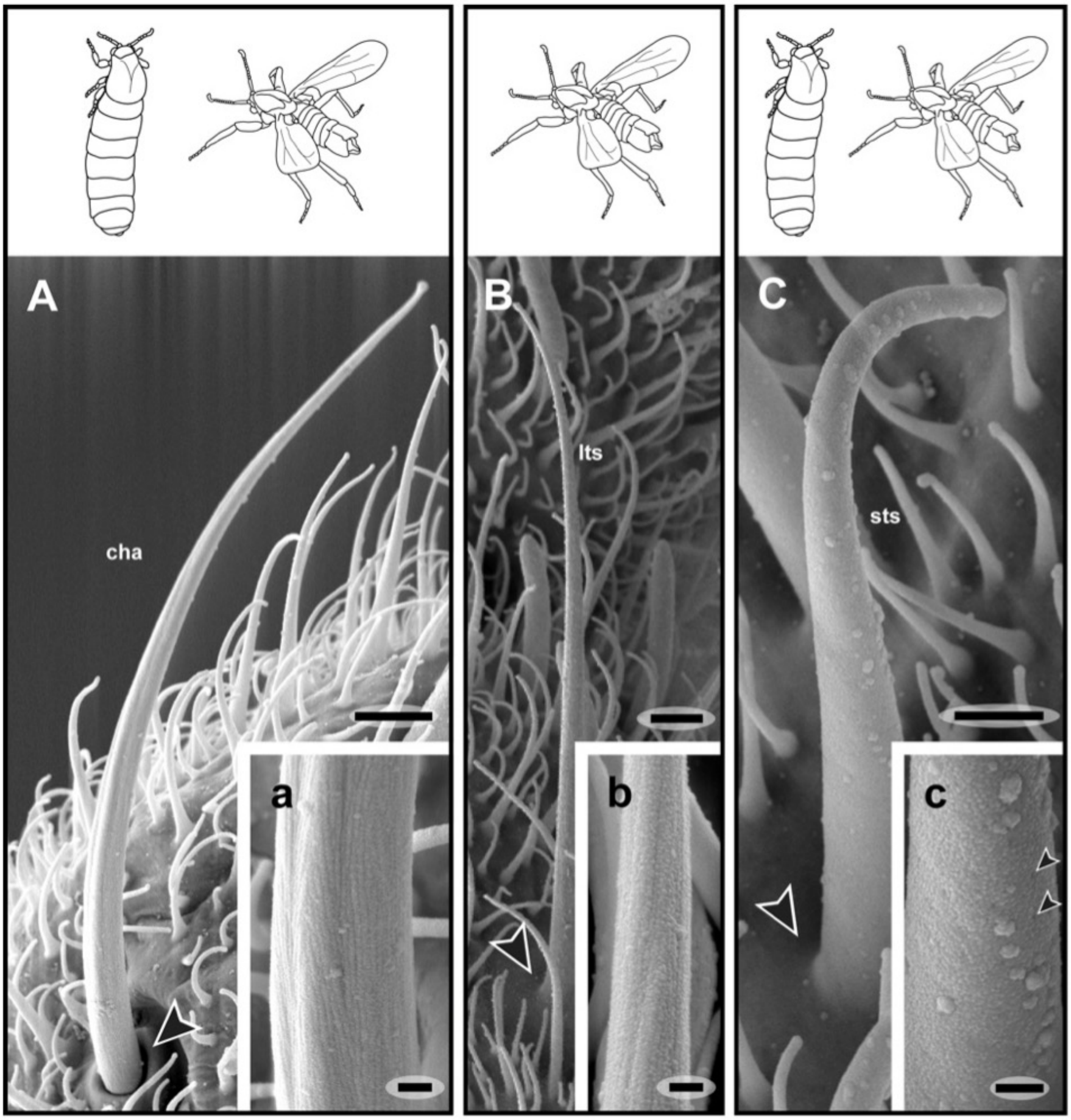

Supplementary Figure 3 Antennal nerve backfill of a *C. marinus* male.
(A) Total projection of confocal laser scanning microscope sections. One antenna was cut for backfilling, so that only one of the antennal nerves is labelled by the neuronal tracer tetramethylrhodamin. The second antenna can be slightly seen due to the autofluorescence of the neurilemma. The antennal nerve enters the brain and neurons within two more or less separated neuropils. These might represent the antennal lobe (AL) and the antennal mechanosensory and motor centre (AMMC). Because a clear glomerular organization is missing in the *C. marinus* deutocerebrum, a definite functional assignment of the neuropils was not possible. The putative assignment is based on position, with the AMMC extending more to the tritocerebrum and the AL to the protocerebrum. Arrow head marks contralateral projections of antennal neurons that form two commissures. In the contralateral AL some regionalization of the terminals can be seen. **(B)** Single optical section through one of the hemispheres showing likely the AMMC with its striated organization. (**C**) Single optical section through one of the hemispheres showing likely the AL. *Scale bars:* **A**: 50µm; **B, C**: 20µm.

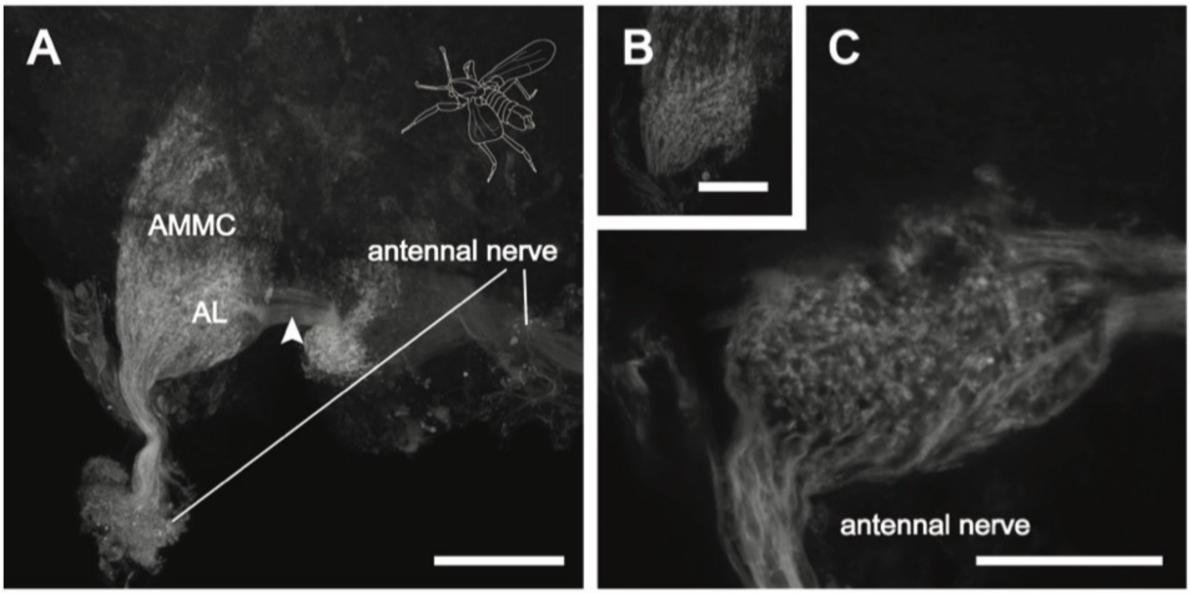

Supplementary Figure 4 **Expression of *C. marinus* Olfactory receptor (OR) and Gustatory receptor (GR) genes.**
(A) Clustered heatmap of OR expression in heads and abdomens of adult males, adult females and larvae. Expression is generally low compared to control genes (alpha tubulin, EF1delta, Rpl18). The Orco is well expressed in all heads. OR45 is highly expressed in male heads. **(B)** Clustered heatmap of GR expression in heads and abdomens of adult males, adult females and larvae. Expression is generally low compared to control genes (alpha tubulin, EF1delta, Rpl18).

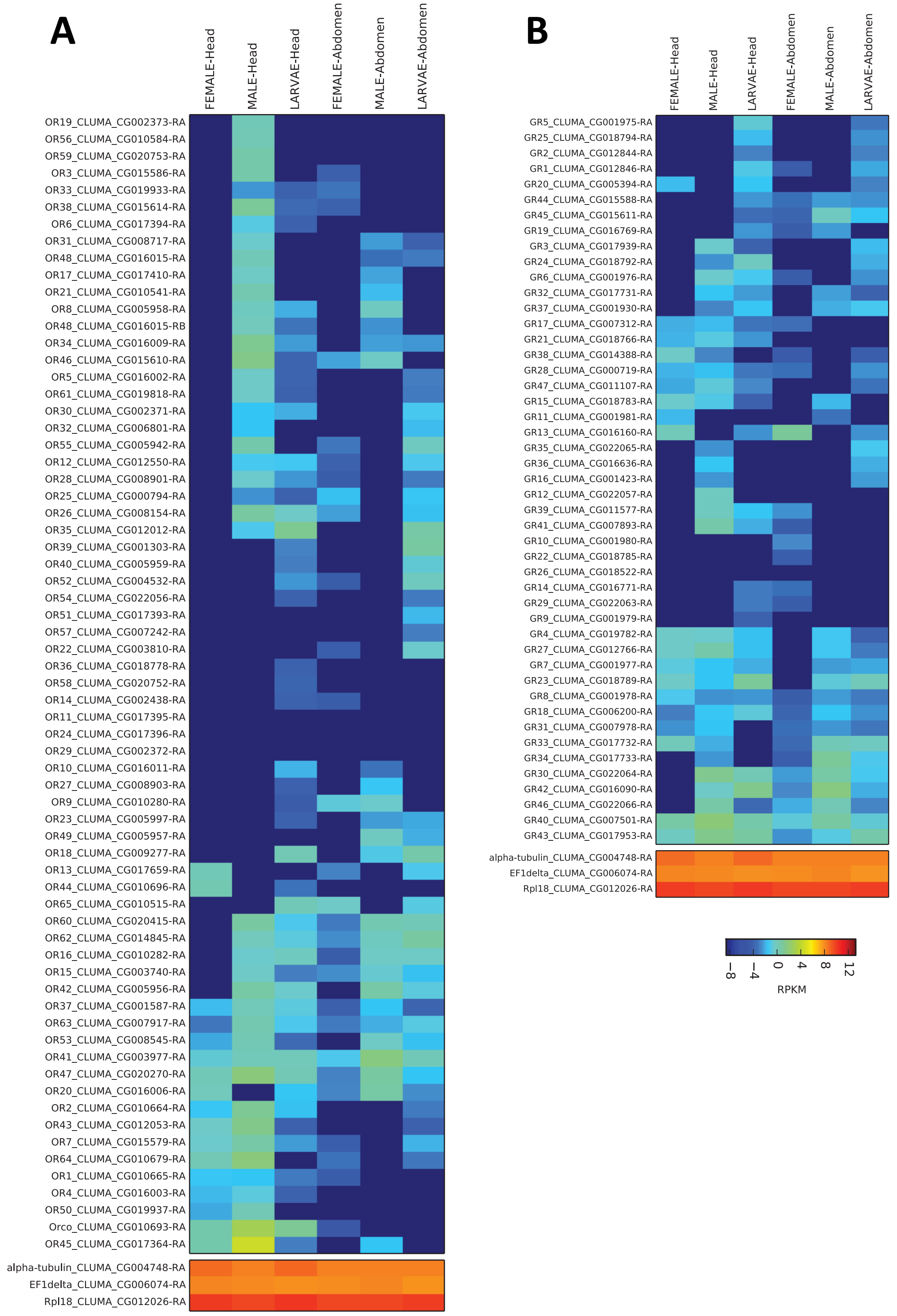

Supplementary Figure 5 **Expression of *C. marinus* Ionotropic receptor (IR) genes.** Clustered heatmap of IR expression in heads and abdomens of adult males, adult females and larvae. Expression is generally low compared to control genes (alpha tubulin, EF1delta, Rpl18). Expression of regular ionotropic glutamate receptors (iGluR) is given for comparison.

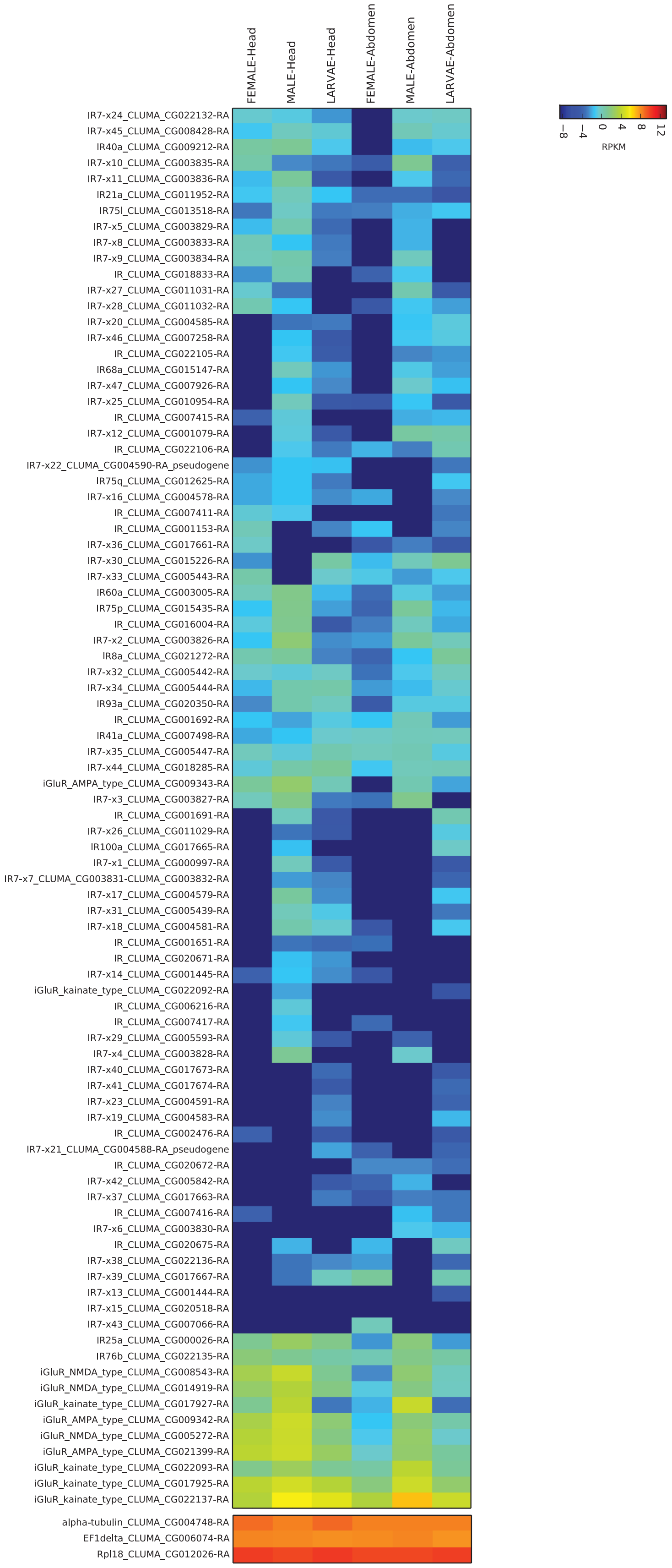

Supplementary Figure 6 Chemosensory protein (CSP) and sensory membrane protein (SNMP) genes in *C. marinus*.
(A) Dendrogram of *C. marinus* (black), *Anopheles gambiae* (green) and *Drosophila melanogaster* (blue) CSPs based on predicted amino acid sequences. **(B)** Clustered heatmap of CSP expression in heads and abdomens of adult males, adult females and larvae. **(C)** Dendrogram of *C. marinus* (black), *Anopheles gambiae* (green) and *Drosophila melanogaster* (blue) SNMPs calculated as described above. Note the duplication of SNMP1 in *C. marinus*, while for SNMP2 two splice-variants were found. **(D)** Clustered heatmap of SNMP expression in heads and abdomens of adult males, adult females and larvae.

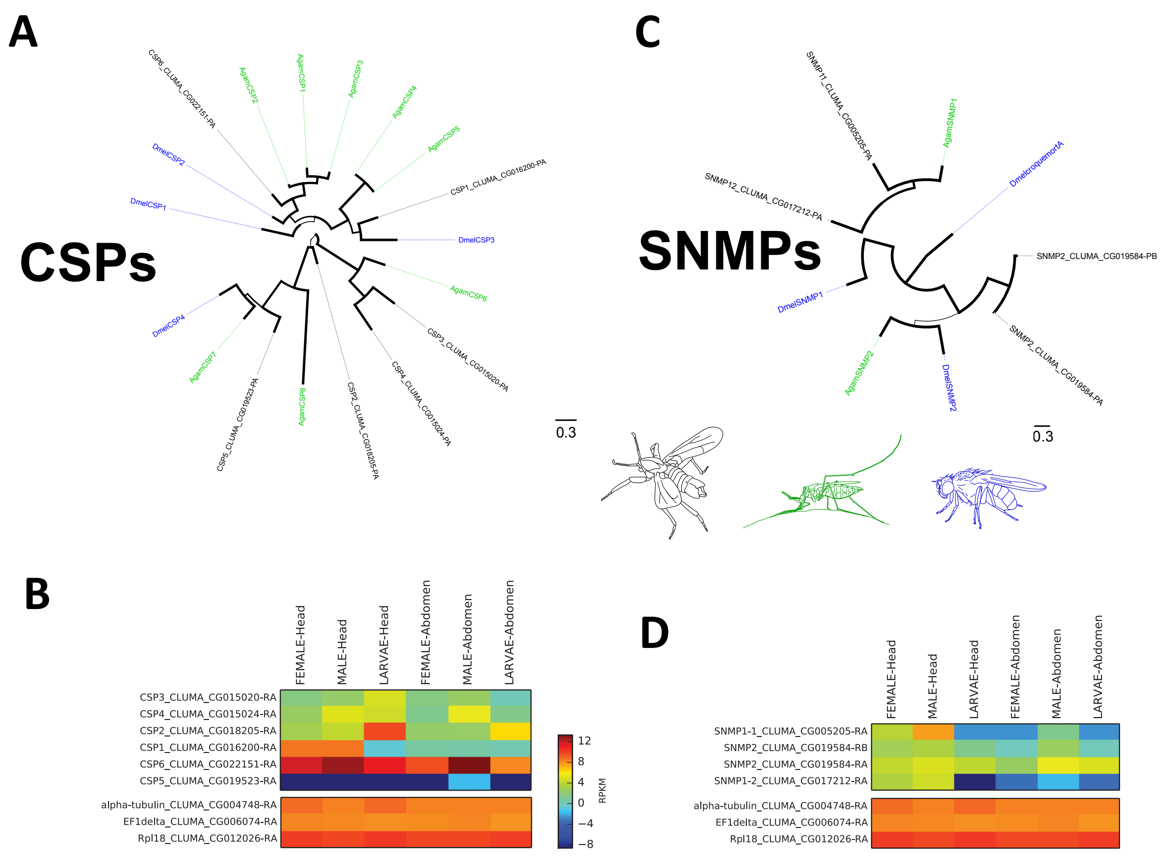

Supplement: Supplementary file 1 — Supplementary Information. [file 41598_2020_59063_MOESM1_ESM.pdf]
