# Supplementary material for: Developmental and sexual divergence in the olfactory system of the marine insect Clunio marinus
Source: Sci Rep. 2020 Feb 7;10:2125. doi: 10.1038/s41598-020-59063-7 (PMC7005812; doi:10.1038/s41598-020-59063-7)
Supplement: Supplementary file 2 — Supplementary Dataset 7. [file 41598_2020_59063_MOESM2_ESM.docx]

>Orco_CLUMA_CG010693-PA

MTSIIESKYVGLVSDLLPNIRLMQGFGHFIFKYINGPIFLRKVYSSVHLVLLLFQFLCIL

LNLAQNTDEVSELTANTITVLFFTHCITKFVYVALSSENISRTLGIWNQSNSHPLFTESN

SRYHQIALAKMRRLLMLVMLTTCIATVCWTTITFFGDSTKLWRDHENNETLTVEIPRLMI

KSWYPWNPVGALYFASLAFQFYYLLFSMVICNTSDVLFCSWLIFACEQLQHLKGIMRPLM

ELSATLDTYRPNSAALFRTVSASSKSELILNDENEKNSNYDLNISTIYNTKSDWGAQYRA

PTTLQNFNGGKGNNNNPNGLTEKQEMMVRSAIKYWVERHKHVVRLVSAIGDTYGLALLLH

MLTSTIMLTLLAYQATKIDGVNVYAFSVIGYLVYSLAQVFLFCIFGNRLIEESSSVMEAA

YSCHWYDGSEEAKTFVQIVCQQCQKAMTISGAKFFTVSLDLFASVLGAVVTYFMVLVQLK

>OR1_CLUMA_CG010665-PA

MKIYSGTLPKVKLSSFKWSETRWNLFSCFLNRFTSSFVKKIIYAARITLSIITLFGNGLI

ISSLTLFSLLDASDFLEASANVPIIFTELLIAVRIIVILFSKQKVWMICNELKSLELNRE

SKFKKFVKENLKEYLRFITTYVIVMVGVMAPIGFPMIQFLINGTISFQINHWTPYDSHAP

ENFLPTSLFLFLFAIYTIIYMMGTDSLLYALMTVVAMEFNILKIDFTQLKFLTDDERNKK

INELIATHNKLLELADDLQSIYGFFLLYNFLTSSLMMCFLAFQISTGGDIGFYASTIAMV

GGQVLLLCFYGQKILDSSSDVASGVYECGWEEFKDKSLKKKLLLVMMRAQNSKKLTAMGF

VDISLPTFTTKEALNSTKQQHFIIFFISFLT

>OR2_CLUMA_CG010664-PA

MVNLRWLNPSEDKINFESFVKFSNICFKLVFFTYRPLDEASSFVKKIVYAARIILSIITL

FGNGLIVTSLTLFSLLDASDILEASANVPFVFTELLIAVRIIVILFSKQKVWMICNELKS

LELNRESKFKKFVKENLKEYLRFITTYVIVMVGVMAPIGFPMIQFLINGTISFQINHWTP

YDSHTPGNFLPTSLFIFVFAINCMFYMMGTDSLLYSLMTVVAMEFNILKIDFTQLKSLTN

DERNKKINELIATHNKLLELADDLQSIYGFFLLYNFLTSSLMMCFLAFQISTGGDIGFYA

STIAMVGGQVLLLCFYGQKILDSSSDVASGVYECGWEEFKDKSLKKKLLLVMMRAQNSKK

LTAMGYVDISLRTFTTVR

>OR3_CLUMA_CG015586-PA

MTCFVYCIFSLCVFGITEAKDFVTASFSIPNVTTVALIFMKTFITYYHKEKIWGICQELK

AIFTRHEESQIKKNLIQAYFSEYILEIRIYGCICVFVFVPIAFPIYKYIAYGTMEFTVDY

WFPFDEYNPRLYPIALLFIVFVCYHFLTYLLATDAILFVIMKILAMEFHILRIDFENLKL

IKSDELKNKLESLINHHQKLLDCGDDLQDIYELMFFYNFGISALILCVLAFQLLFANDAN

AYTFVISYFLLIAGQAFILCLNGQKVADNSMKVAEGAYFNGWEDFHCNKIMKQNFFIILR

SQRAKQLTAMGFADISLSSFAQIITTTWSYFSLLKRVSSYDD

>OR4_CLUMA_CG016003-PA

MDAELYKPFRLSLFFFKRLGMWQDGEQTWTYFVCGYLFHFVFVYLYIFGQVIYLINSADL

LDFAEAFVLTTTFVAQAFKSYNFFMKLKRIKRSMENINNLLEKTDHETNFGRKLIRQEVS

LCYKVYLIFWSSAIVTVISGAFAPVLSSKSPYKVWFPFDTNKETNEVGFWISSYYLVFNS

IPISTLDITLDTFPVIFMAFAIGFLRELSERLKKIRNNHELVQCVVIHKEVKLFIKDIHD

NFAEAIFFQGIMSSLTLCTGVFTMSVTENLTQLIRIITFLIPMLLEIFLPCYFGNELSIA

SSTLTTSIFQSNWIDGDMTFKRALMIFTECNRKELKITSLGLFDVNIATFSSIGRSAYSV

FNVLKQSTNRNSY

>OR5_CLUMA_CG016002-PA

MDKNLYKPLAFCFEVLKRFGLWQDGKQTWSYFFLGYLFHLVFIELILCFQIVYLTKTTDV

MDFIESFSHITALFAAMMKSFIFIWKLKSIKKSVDSLNEMISNSRFSNNDHMKREVLIVF

RIFKIFWMLFACSSLTGFVPIFHNQLPFKMWFPFETVKEKSQIGYWIATIYLQILAFALG

SIVVSLDTLPVFLMSFATGFLKQLSERIMKIEKYDDLIECVNNHRFVKNFIKEIEDNFAS

AIFFQGLISSLTLCTGVFIMSFTESARDFTAITVFLIPITLEIFLPCYFGNKLSVASSAL

TTSIFHSEWIEKDRKLKKAAMIFMECSQKHLKITSLGTFDVNIATFSSILKSAYSFFNVM

KQVN

>OR6_CLUMA_CG017394-PA

MEVTEEIKLITMEDFTKMGIKCFKLIGLNLESRKEVTKKPKAFMLLGEFHFFLFLINIGL

IVCALLGYAYKNLHDIKIVARVLPNLTNAPYLAIKLFVFYWNRDKIKDALSILEESFPKT

EEDQLNLNVQTYLKEVKMFVKGFGFLIIVLQVVLIISQVVLIFMFGTTKLPLDIWLPFSY

ENFIIFGAVSLWMGWICFVVTTGTYAAEIILFATISLSSMKFDSIKRKCENIGVNELKTE

EEELTNIKEIVEEQNEIFKFIFDWDKIFSSLFLYNFVQSSILLCFIIFLLSSSRDISTFV

LYFPYLGTSLNQILLFCFLGQKIVNSSSSVAIGIYNSKWHQIKSLKIRKALLMILIRSQK

PSALTLKKFGVMSFETFTKILAAAYSYFTLMKSFM

>OR7_CLUMA_CG015579-PA

MEKYFEFNRKTLINLGFAFDRDEKKFEKFLKVFTLLTVCLMIFQSFVEIGVQSKSKITIS

TISTISVVLYNIQGLAKFMAILINQRKLCDIKRRLSDFIKVQDENQMKARQLQIQRFPWV

IKFMYIINIGCVWLFTILATFALIYTVITHGNFSKNSIFRRWYPFDEIKYFIPIFIYERI

TNHFLTSIHTNLDGFILLLLGQFCILFQNHGEYFENILNNSMEISNFDNKLKAAIDYHNE

LLELSDKLVTIYEIPLFVNVLNQTSTICFIAFIISTFPLDIAIASFIGLVSSIAQIFILC

WFGDKIKDNSLEIGDKILNSKYQNLNKSQKKSLLLIVMRSQNARYLRGSKFFNLELSGFT

SVKN

>OR8_CLUMA_CG005958-PA

MENYYQFTKKMIFSIGVDFDKQEKYKFLLKILRNISIAFTVLNLIHSLMYFATSNKKDYH

NISSVSMTIFGLQSLIKIFVVCYYRDSFVDIIERIKSRFEKHDEHEKEKSWSFLKRTRKF

LVNWFLAQMVCLWSFNLLPLLTMVIGFFSKGILLKTFPYGYWFPFDTDRYYFIVFCYQLY

AGHVIIAIQMIMDQLFLLISAEIKTQFDRLGDRISKIINNTDPKESVNTKKELNEFIDSH

NDLISIFNDFNDVYTGAVLFDVVTMAVGICFVGVMTMNLDLAHAIPSALGLILSVGKMFI

LCWFGNMISESDSAGRIADEVYGSNFLNLNNSLRKDLVIIIQMSQKPRTISVRNVFSLKL

SAFSNIIRTAFSYFALAREFF

>OR9_CLUMA_CG010280-PA

MSSEWLKVFVKPLKYLKTTGIDTSRNASKKFIAYSVIMNLLFVDLFSFLSLIPVINDVSN

VVDFAHKAGPASSFIGYNMKVKNFIIKRKKIESLLKLTEDIIESFSWIDVKEGKILKARI

AFAEKILKMSMILMLSASCSNPIIILISHNLPIPYNVEDNQFLFWMSAFYQSFTSLVFTP

VLVIMNVLPIIFISFAAGMIDELCLKLQGIAGHIEKKTDSNEKGLSECIKIHIKIKVYVT

EIKDAFGTALFIQAFLSTVILENVLLQDEKAITSLPSVAFMLPMLLEIFLPCYFGNELAL

ASEKISDSLFYSQWQKETKKFKTSMKIIMENSKLSLKIHIFGLFELTLKQFIRIVNSVFS

LYAVLKNIAT

>OR10_CLUMA_CG016011-PA

MDAELYKPFKLTFFILKKIGMLQDGEQSWIYFVCGYFFHFIVIYVYIFGQLTYLINAADL

MDFSEGLALTTTFIALSLRSINFFRKINKIKTSVEALNKLLRKTDHEASIKDRKFIRQEV

SFCYKVYLAFLFSGILTLTSGAFVPVINNKVPYKFWFPFDTNKETSEVGFWIASYILVLH

SFPTGILDISLCMLPVIFMAFAIGLLRELSERLKKIKTIEELVQCVTIHQEIKLFIKDIH

GNFAETIFFQGIMSSLTLCTGVFTMSVTENLTQLIRIITFLIPMLLEIFLPCYFGNELSI

ASSTLTTSIFQSNWIDGDMTFKRALMIFTECNRKELKITSLGLFDVNIATFSSIGRSAYS

VFNVLKQSTNRNSY

>OR11_CLUMA_CG017395-PA

MEVTEEIKLITMEDFTKMGIKCFKLIGLNLESRKEVTKKQKAFMLLGEFHFFLYLINIFL

VICGMLVYAYKNLHDIKIVARVLPNLTNAPYLAIKLFVFYWNRDKIKDALSILEESFPKT

EEDQLNLNVQTYLKEVKMFVKGFGFLIIVLQVVLIISQVVLIFMFGTTKLPLDIWLPFSY

ENFIIFGAVSLWMDWLCLVISVGAYAADIILFATISLTSILLCFIIFLLSSSRDISTFVL

YFPYLGTSLNQILLFCFLGQKIANSSSSVAIGIYNSKWHQIKSLKIRKALLMILIRSQKP

SALTLKKFGVMSFETFTKILAAAYSYFTLMKSFM

>OR12_CLUMA_CG012550-PA

MKLNFSSFQSFSIHDRVYKYLKVFSKLFVFCGKIDNIHEFKGKMVEISENKTLEEMESDK

KYKRIIRVVIAALAVFYFLVTLVLILGSLIFIPTMGMPIYVKVPWTRSNSHPSHEVNVAF

LILYMSVNLLIIGIDALFMLFTLHCVAELSVCCIYASKIDRQTNRDFIKIFVKKHLRVLH

LIETSSEVFNIMSFIQLLTSFGMAVSTLIKLTSEIDIMSITIMSAIASQLFIYCFLGSIL

STMCEKLQESLYCSKWYEIKDISIKKNILIMIGEMQRKQGYSAFGIVNIDLETYSNVIKS

AYGFFNFLLKVL

>OR13_CLUMA_CG017659-PA

MNNIVEIIIPKFKRNCLFEIDFLFLGLSGCSPRHMLNFKIFVVLFLGIILQILPGILFLI

KHSDDIQEMLMCGYEFLTFIITILKIFMLFIFRSKTVSVVDDLKSSNSRWADAGMKVEHI

SFMYTRNMYILIFVATFVYTIFPIVIYFLFTDHDDANNTQWPLPIAVHYGINFSHYPLIY

VLVLIVLIPQCWIFINACYGIEIFFINLLLFCFSYFRFINFEIKRLRKDMKCFLTKDVDI

VKRILNIVVIHNKGIAISKKIEEILNLLMLVLYVVNTFILGFLFLEFHVVSMVINVILII

NQYYDHQKIIHERFHEIKKIVNDTMNFVKVLIAFIVIHLILFIFSYLGTKLMDESDAIGL

AIYDLPWYQIKNYDARKYLLLTLLRSQRPIKITAGKFFPVNLQSYFGALKAAYAYCTVLL

EAI

>OR14_CLUMA_CG002438-PA

MENFDFLSPLKVPLKILQVLGFWFDENASKCYKIYGIFIQTFFIDLCIILLIIEFFRVDG

LFIKAFILAVTFTFLGSMKTKHLMIVSYKIIGIMADLKEIIALLDSNVSDLRILKEKARQ

VTKLFWFYWILCMIVAFCGCVIPWINYFQNPEPPYMVPVPTWSPFNHEQNIFGFVIVTIY

ETLAAAVYCGEHIAGDILPVYFFNVSSGLLQELNKRVTNIGKKRQRSMKSKKVKQDENFL

ELLKCIEIHSKVKSLLHEIENIFSIMVLIPFVVGGIIMCTTGFTLSKISMMEEPSSFIFM

FTYLITTIMVIFLPCYFGNEVLLASQEFSTSLFHSDWIEENKNFKTAMKMVLENTKKPIE

ITVAQGVFPVNLLTFLRIINSAYSFYAVLQSIN

>OR15_CLUMA_CG003740-PA

MEKVDSFSLSFPRILNKATKFHFIHSAVKVKLSRMIEPKEVFQTLIKFAKYTGIWQMPQT

SWIYRVYGGIFMFLFVFLFLFFQLNGPDIKTERKHIKRFTKEVRLLHGILLVCAICSAIS

VSLIPFFNISKHELSVRMHFPMLDYKNNDTIFITIAVLQLTIVLGAIANITLDVFPIYFM

CYAIGLIEELGDRLENIGSNHAVIGHQEENDGNDLNELLKCVEVHRKIKRLVSEIEEHFS

FVIMLQGLMSSLIFCATAFMLSLLSPVKDFPLFIRFGTYAITMVIQIFLPCYYGNEIATS

SMKLSTRFFHCKWKKRDKKFNQAMIFFMENTKRTINIRAFELVDLNIETFNGISKFAYSL

FALVSRVNNRE

>OR16_CLUMA_CG010282-PA

MAAVLKSLFIEDENEVNFDSFINFPISCFKLIFFSFVRPERSASIREKLFYNIRLTYFRV

LMVLFVLCLALIIAYAVIEAEDFVNATMVIPDAATTILIIMKSFITYWRRDEIWEICEEL

GILSNGRSATDITKYGIANHLKEYLRGMRIFTVMYASVFGPFVLQIIAYLIYGKRELMVK

YWFPFDEYDPGNYPFALLFSAVMNWNYITFFVSTDSMMYTLMTVLAMEFDILRIDFMNLG

ASDVVEMKEKMKNLIKHHLRLLNYADKLQEIYGVLLLYDFVIGSMMICVLAFQFLIAKGL

FTSVYIASYLSVIFCRIFFLCYKGQKVADNSSEIVQGVYFSGWENFTDIKLNKQMLLIMI

RSQRPKFLTALGFADVSLENFSKCKLTRIKNLLNKSADYY

>OR17_CLUMA_CG017410-PA

MEAFLDNFRRNTKNEINFDSFTKFALFCFKLGGFKFQQLSADADLKERFYHKFQRNYLRV

VFIAFVYGFFSFIAYAVRDAKDFVSGSLSIPNMATVLLICLKTFITYYHKEKIWNICQDL

KEVFAKRETNQNEKYNIKAYLKEYNLETRICGCICLIIFLSLAFPIFKFLNYGTMEFTIH

PFALFFAAFSGFYYIILGLATDSMLFVLIKILATEFDIFFEISALILCVLAFQLLFADDT

TIYTFCASFFFLMAGHIFLLCSNGQKLEDNSMKVAEGVYASRWETFQSIKVKKHILFIMM

KSQRAKRLTALNFADVSLSSFAHILATTWSYFNLLKRVSNGT

>OR18_CLUMA_CG009277-PA

MKLISLFRYRKEVKKLISQLSQIFKEFKNGPKEIQEYHEKVLKFSFYFIIISSSITTITL

SFYFYIPPIYHFISLLSGKSNLNYPLALKADFVFFDPRDNDYVFALYTILSRIYMTFFCA

QFCVVDIFLDTLSFYIISFFHCINLKLKLLSKEIDENHLKEIEVIKKLKEIIENHTTAIQ

MVKLLDKMLRINLITQFLLYSLSFCFVMFNFAMITDSFDKLVYVLCFVVVSEMTQFIFCY

LLTKLQEQSQLIGLSAYDIKFYNYSTKVQKMVMLIIMRSQDKKIEVTAGKFYTANLESFG

NALKTSIGYFTFMKSIYAVET

>OR19_CLUMA_CG002373-PA

MENFDFLLPLKVPFQLLKIFGFWFDENASKSYKMYGILLHVILADLFVIGQFVEFFRVDG

LFVKTLIIAVSFTFLGASIKTKNVMIVSKKIVEIIADLKEIIALLDSNVSDLRILKGKSR

QVTKFFWVYWIMCLIVISWGSFIPWINYFQNPEPPYKLLYPTWSPFNHEQNIFGFVIVSF

YESLTSGVFCGVVVAEDIFPVYFFNIISGMLQELNERISYITKKRENSGNGESTGKRTKH

DEHFMELLRCVEIHHRIKKVIQKVENIFSAMIVIQGIMSVILICTIAFTLSKMSIMSEPS

LFIFMFTYMVPMILEIFLPCYFGNEVSLASQKFSTTLFHIDWIEEDKNFKSAMKMVLENT

KTPIKIAAAQGVFPVNLWTFLRIINSAFSVYAVLQSMK

>OR20_CLUMA_CG016006-PA

MTSIDILNDLLNITQNYDLLSHELIRKQVAFSYKIYLMLWSSALVMCTAGVFVPFINHKL

PYKVWFPFETDIEKNELGFWVASFLVVFNSFFGSAIDMALDILPVTFMAFAIGLLNELSE

RIKKVRTYDDLVQCVVIHRKIKLFVNEIYENFATAIFIQGIMSSLILCTGVFTMSVTKST

ADFIQITTFIIPMVLEIFLPCYFGNELSIASSELTSSMFQSKWIEGDSKLKKTLMIFMEC

NRKELRLTALGWFEINIPTFTVIGRSAYSFFNLMKQMQ

>OR21_CLUMA_CG010541-PA

MSSEKVYKTKDFFTLAVGILKYLGIDCINGTSFKNKIIFYVFISIQFFGIWSFINFIVWN

INDKDKFFDVVLCFGLIIYTMLANFRGLNIKHKEERIKKLFDELQDLFPNTNVDQKRHKI

PELFRFLKVLSKILLSFSLFVLTVFASVPFFMKFYSLLTSNDIKMAMFYTIQWRPENNAL

FFLIYLQQIVYSLTGLIFLNRNDTLYCICFELICKLYEVIAHDLRNLKNQHELGILIENH

EKLLELTSKFDEVFCVGNLLNILLSILAMSVGLIQVLATESFEMKIKYAVCFLVLIPQVF

ILSFWGDKVEKASESIAKAAYDRWKIEDDKKMKTSLQLILIRSQRPQRLTGYKFIKLSCG

TFTAVIKTAYSYYTLLMTVHEND

>OR22_CLUMA_CG003810-PA

MDFELFFPFSIPKRVLKAFGTWIEERSTFFTYIKGIVMHLFFIELDTYLQLVFLFTFEDF

EDFALLMTLLPTCIGLFFKTSNFAFKIKQIKQLMAIIEDLSTYCTKTEKLKKYLKKVDKI

TKLFMTLAFCSCICGAFHTMYELPYKMWFPYDINNRLNYWISAIYQIIGSTILAQVTICI

DLIPIIFMCYVIAFLDILCDRLQALKKEKIKITEFEDFLVPSNEELSKCIEFGIKIGKCV

ELIEETFSTIFFAQGFFSTLILCTISFSLSLVTDPVKFIRLFSYLILMLVEIYLPCYFGN

EITLKSEKLSENLFHSDWPEENKKFRSNMLVAMEKMKRSFKIAAFGVFGVNMKTFSDVCR

STYSLFAVFKKIHGRKENV

>OR23_CLUMA_CG005997-PA

MDRLKCSFGEIKKKLKKTLTNDLDYKLELNFIEKLFKIIFLDIFNRSGKVPKKILIFATI

FFLFWSIIHVKNIFEDFLYDRTRQFTATTAALISFQIATKIAVICMKADSVSKMREKIVD

DIRNTTNDEMEIEKSYRIFIRVFLSVFAFIYAITAFISAITNLWATVSSKPIILAFNVQI

TWNSPNLHPSQEINFLVLLVWQIIILVLAVGFDSYFVLVTIHCVAKLSTCGSHALKIDGK

TDGKCISILIRKHLHVLELIELSRNVFNPMCFYQLLSTYGLIVSISFNIKNGIDLVSVMT

LAAALFQLFLYCHFGNILSSMCEKFQEAIYCSKWYEIQSISSKKKILFMLMMAQRNKEYS

FLGIKSINLEMYTYVVIQAYRFLSFLLNVM

>OR24_CLUMA_CG017396-PA

MEVLREDEIGSFQMEDFTEFGIKFYKLIGLNMIPQRRTTWKEKLFQIFKSFLFVTFFLNI

FLIEASMMIFVFKNSNDLSLIARVLPHVINLPYVVIKILYQYINRDKVAEIQGSLNEIFP

KTRKEQNIFGTKNYVDELRKFTKIYGTVLVVGFTAFIFKGIVILFISKTNELPTSLWTPF

HYERGIAFIPFLLWIYYIVWTKIIGSFAAEITLYTMVTLFSMSFDILRYKCEHIEMGESK

LEDDELKQIKDIIIEHNKILELSKEWENIFSTFFLINFLQSSFFLCFIVFLMLSNGGMML

NYVPYLFAALSQTILICFLGQKMIDSSSSVGIGILNTELFNSRNTKMKQAILLIVLKSQK

PSVLTMKKFGAISIETFTKILAAAYSYFSLLKGLI

>OR25_CLUMA_CG000794-PA

MNFELFFPFSIPKRVLKAFGTWIEEGSTVATYIKGIVMHLFFIELYTFLQLVFLFTFKDF

EDFTLLMTLLPTYIGLFFKTSNFAFKMKQIKRLMAIIEDLSTYCKETEKLKKYLEKVDKI

SKLYMTLAFCGCLTGAFLTIYELPYKMWFPYDINNRFNYWISAIYQIIDSNICAQVTICI

DLIPIIFMCYVVAFLDILCDRLQALKKEKIKITEFEDFLVPSNEELSKCIEFGIKIGKCV

ELIEETFSTIFFAQGFFSTLILCTISFSLSLITDPVKFVRLFSYLIPMLLEIYLPCYFGN

EITLMSEKLSENLFHSEWPEENKKFRFNMLVAMEKMKRSLKIAAFGVFGVNMNTFTDVCR

STYSLFAVFKNIHGQKENI

>OR26_CLUMA_CG008154-PA

MTVGNFWNSLIKFNQETNLFKHNIAFIWTTGLSVWHKGTSYKAFQYLTILSFILFYITSF

WDVYNHLIVGNIRGVGENMSTAAILMTGLVRFLIYEHFEVMNFINEKKLQDVNGAFNALR

AKKFRRNLYSVVCLHTQGYMVIGSFFIFPIIELIFYGTYRFPQPSYVPLDFASLPLIIFL

TCYVISCFATLASGVNVVATTLYVNTILEYLSVEFKILGLAFEKVFDEHDNEKAVSEFKK

LIDHHQKLLSTARKIKNLLSFPLFYQMITAGIILSTSAYEIYSVDNLMSIKFVARLNYAL

YVIIDLLIASSASENILLESKNIKDKIFNSGWLNIVQEKKNRLLFQLSVQLSYQPIVITA

MGFFKPSYSTLLDVMK

>OR27_CLUMA_CG008903-PA

MENKEINWYYSFNIPLFFLKCIGFYVENNSMFLSFISLVLHLIIMGSATFCYTIFFIETI

QSGTIQEISYVTNLLFTFYGSVLKSFWLLKNFQKIKEMLNDLKIITKVHKFNEGEKKCLK

HLRIDRVVKVVKIYFSAAFVTGLMGTLNALIKYNEKEIMFETWWFFDYKASIIHYWTIFI

YQHLFLAYIVVIACSTESILILFMNTTTALIDDLIVEIKLWKHNPIKKILRQKVFFKNSQ

DYHALKKLEIAMEYHLKIKKIVEDISKHISFVFFVQTFISSIVICGSVFLMTVLSQKHET

SDFFRTAVFGCLMTFQIFLPCYYGNELSVASDKILSTLFHSDWVSSSQKYKNSLKIFLEN

TKRTIKVRSFKIVNIDFETYTTIMNAAYSLYALMNKINQK

>OR28_CLUMA_CG008901-PA

MSSENYLKCFKFPFFVLKVFGLWQEKSSSWIYRLYGVAFHLFFLGYIVICHSMFFLDTLL

TGDMKEICEALSIMFTVYSIFIKVIWFLIRLKKIKNLFQHLEELLKVNSFGKMNERNLMI

RENKRMMRIEKLFFGLPIFTCLVTVIKALSFDMKRRELPFKTWVYWDYKNNLPSYWFTVI

IQCVGAPILSLLNFSIDMIQIAFMHYLNVMLKEFSVEIESLEPDKMSFNDTKEYDRLCKC

VDCFVQLKDLGSKISTHLSTTFFIQAILSALILCTSTFLLTTLSFVNEAPTFIRTTFYCL

TMLIQIFLPCYYGNEVTLTSDKISTTLFHSKWVDGDQKYKKAVTIFMENTKRSIKISAFG

FVFVDIETLTTICNTTYSLYALVSKIAQK

>OR29_CLUMA_CG002372-PA

MDNFDYFLPMKVPIKIFKMFGFWYDKEASKFYKAYGVFMHLFFIELFLTGQLGRFFLTDS

LVERAAITSVLFTFVGIFAKASIVMFITDELVLVIEDLKHFISFLEVGDGSLKFLRNRVN

QSTKLLRVYWIICYTAWFAGALIPMVNYFRNPIPPYAVAFPVWSPFDHEQNLFGFACVAF

YQSFTTFSVCAIVVIGGLVPAFFFNVNAGLLEELNDRIANIGKPRKSIGKSGKDKKKQLK

STKFRDLLECIEMHHRVKILISRVGNIFSTMILAQGIMSVITICTTAFTLTKISIVTEPS

LFIFTFFYMMPMILEIFIPCYFGNEILLASQKLSTSLFHSDWIQEDKNFKTALKMVLENT

KNPIKIAAAQGVFPVSLVTFLRIINSAFSVYAVLQSIK

>OR30_CLUMA_CG002371-PA

MDNFDYFLPMKVPIKIFKMFGFWYDKEASKFYKAYGVFMHLFFIELFLTGQLGRFFLTDS

LVERAAITSVLFTYVGLVVKTTNVMLITEEMESITEDLKSFISFLEVRGSSLKFLRNRVN

RFSKLLWVYWTMCHIVVFSGTLIPMVSYFRNPIPPYVVAFPVWSPFDHEQNLFGFVCVAF

YQSLSTFALCGMVVIEDLVPAFFFNVNAGLLEELNDRIANIGKSVGKSGKDKKKQSKSTK

FRDLLECIEMHHRVKILISRVGNIFSTMILAQGIMSVIIICTTAFTLTKISILEESSLFI

FMFTYMVPMILEIFLPCYYGNEILLASQKLSTSLFHSDWIQEDKKFKTAMKMILENTKNP

IKIAAAQGVFPVNLVTFLRIMNSAFSVYAVLQSIK

>OR31_CLUMA_CG008717-PA

MELNYSSVFNLPFFFLKNLGLWQSKSSWKIYRSYGFILYLIFLGLPLVCHTKFFFYIVKN

GTIADISHAINILFSLYGTLFKTFWFVIKLESIKEMLEVISTDHKTHQPRKTFLNNFSSY

GNIMKVVKFYYGSALWAVLMASLNSVIHYKRKEVIYETWWFWDYKSNDNYVYWPFLCYQI

LLILYGCLVNYSIDIIPVLFMNFASMMLNNLSDKISVKNNVKENEVKDIKEIKNLVEYHK

KIKQFTDDISTHTTFVFFIQTFISSFILCACTFLITTTSPINNTSEFMRTFLYGCSMLFQ

IFLPCYYGNEVSATSDKISTSLFHSNWEGRSKKYRKSVEIIMENLKHSIKIRFNNFITID

FGTFTKICNYAYSLYAVFQKVNVK

>OR32_CLUMA_CG006801-PA

MLKGSFEIDLCDEASISTLLEIISRQGMEEKMLRLTIRIWNLKLFIKLSNYQMFTKYSVF

YILNKKLFTLKKKILADGKSNEIDFESRKIIEKHRRVIRMAIFVTIALYATVILGSFLCC

LIKVDEFLMITEIQLPWTRPNNYVSHGMNMTTMMVWISHMAFIVIGSDSFFILTTVHTMS

EASVCGHLVSKIGRNTDDKYLNDLIERHLHVLDLIESSNTIFSVINFCQLISAFLMIVAA

LFQLNSVVDAVSLLIIVAILSQNFVYCFLGTCIETSCEQLEQSLYCSQWYNLRNVSLKRK

FLMMLVMSQRKRGYFAIGIKPMSLETHAELLKAAFSFLNVLLTML

>OR33_CLUMA_CG019933-PA

MKFLNNGLDFEKEFQFIEKIYKIISLDIFGGSKKVSKRFLLQSAIYFLFWLVVHFKNVFV

DFVSDRAKQFTGIIGISVFYQMIVKSYAICSQLDIVSKIKSTMTEDAMNTTEEEMQIEIK

YRKYSRILIAGIAFLSCLVMGISVLWSLIFIKQMVMITNIQIPWTLPDTHPSHEINLIMM

FLWQLISTPLIASFDSFFILVTFHCVAKMSVCCSKASKIDGKTDENFISDLVEKHLRVME

MIKISAKIFNQTCFHQLFTTFGVIVCSTFNFTNRFELFPFVMLSAAILQLFFYCFLGNIL

SSMCEKFQESIYCSKWYEIESISIRKKILLILMMSQRRKEYSFFGIKPLNLETFADVVKQ

AYTFINILLRLL

>OR34_CLUMA_CG016009-PA

MDIELFKPFKLIFKKFQWFGMWQDGEQTWAYFIFGYLLHLLLVNLFILCQLLYLINSVDL

MDFIEAFVLLTTFIAISCKSINFFLKLKKIKKSYDELKDLLQSTNYEESVDRKTLKKEIA

FCYKVYLAFWSSALVTCASGAFVPFLSSKLPYKVWFPFDVNKETNEIGFWIASLYLIFHS

FIVSTLDITLDIFPVVFMAFSIGLLNEFSQRLSKIESNGDLIQCIIVHKKIKHFVNGIHA

NFATAIFFQGVTSSLTLCTGVLTMTFTTNLTQLIRIVTFIVPMVLEIFLPCYFGNELSIA

SSHLTTSIFQSKLIEGDQKLKKTAIIFTECNRKELKITSLGLFDVNIGTFTIIIRSAYSF

FNVMKQIN

>OR35_CLUMA_CG012012-PA

MERIYKLFVGPINPKKPNESFALQIWILKRTGYWEPDTDSKLIKTFYNVYSYVFRWFFLY

IYTFTQAMFFLNVKDLKGSKSFFLLLTQISLLYKVENFYANRHRIKECERFLRSTMFKPK

NEDEDLVVKDAMAKTFLLPFLFLTFAHLTIGQWAISVFFDDKHDKLRLPISAWYPFSYEN

PWVYSIVYIYQVYGISMSAVVNSATDTFASSMIFHLDSQIKRLGIQLSKVEEVDEHMKTV

NSHVNKLTKLNGKHYGEVMHCILFHKNIFWCKELEDIFKGSVFGQFIASCVISCMTLLLI

ASSTSSPLKVINQSTYLVTMMTQLSLFCWSGNELTHSSHQLTQKAFCSNFIHFDKKTSQI

LLIFMQRTIRDVAIKAGGLFAVQLSLPTFVAIMRSSYSYFAVLNSVQNKEN

>OR36_CLUMA_CG018778-PA

MEKISELRHKILELNYRINLKFWKNNLIFLLIVINHIAITFLYLIYGYSSYADFSGTDMT

EFFILMIRSNFYWAFVYRNSDKIKLFASKIISDRKTFSNNERIIKICRNYERSENYVLLA

FIISGTLSALMAIVMTFFTKFNNFHLPMNFYIFFLPLNESSINWWINYFYQANVIAFGSI

LANIYFPLTLWLINHSSWGHEVLISCMKEFHEVVERKSTSNDVEIDHKLNDVVKQSYLVL

YDHEEIRQFLKFNFLIEFSLLAIMFCMSIYTITTSAFVSSFIFTSMLYPIAQVYIYCAIS

SKVMTQVDKLVDVIYETSWYTLNTNQRKALQLMLCKFQNFKGFNGIFIKVDMDAFQKLMI

DIVSERETKYD

>OR37_CLUMA_CG001587-PA

MKRVMGLFGFHPDRFNMISFQQMITLLRSISYQNGFNFENLSIIDVIANVYTTSLALKLF

AFHHNFEKISILIDQISNLMTETTLNSCSRKSKIFDYFVHIYLSVYTLLISVIYLLMFFY

EESSFDPNELFPFETAIHPLKEISIFVSMLSFAFLEISSLIYQCCFFNICIHVGSLYAQI

ENDIQKMENGNFSKTINNSLIEVRELIVDTNKYFRGVMCLSFIIDVAYIGIALTMFGKLD

DALTFVFYAPMVIYDLWIFCYGSSLMVEKGDTITDVIYNIPWYNFNKKDKTLTQMMIQRS

QIPIKVNIFHWRIDRELFLQKVKNGLENIKSILTKYAFSTLSTGYDCIMKYLN

>OR38_CLUMA_CG015614-PA

MILSKLTFFILFFSNLLRNALVDFSGDHTKQFSPFIGIFSDVQEMILNDVAIFVKEEFEI

EKKYRKITRGIFLAVSFFYSMVLVLFFLWPIVSAGTIVMPYSFQLFLESPFMFALNYAIL

ICYVTSAIYFIMAFDGFFILLSLHCIAKFTVLCHLASKIGDFKNFELTTEKCQSHFINGF

VRKHNHALKLVATANNTFKFINLGQLFTALGMVVVVSFLIQFNVPFNVYINFLTILIQLF

YYCFIGEVMLNMSDALSQFLYCSQWYALKSIATKKKVLIILMITQREKGYSAGGFVYMTL

RIFSIIINRAYSFITVLKSFI

>OR39_CLUMA_CG001303-PA

MSTQRDALEIFFKVFNYYGLSAFKKREKKSFLFFLLFVFAVPGNTIILLLATAQATTIDE

KVRNFRLVPLYVAALVKGVNLVANLKDILDLIASMKEMMKEIDDQEIVKRANVQSMRILI

MYLVTGFSPLIATQIFSLITQEQVIQLPSLPTGYESFQICVFAIYWILYNFGANYSTLLS

LLLSFILSYFLTCFQAYSEFLCGEDQPSKKTIQNFYKLKRTAKKYSEIFSTVILYQVCLA

MLTLASHAFAIADLKNIFSVESIPIFLMTIIIILVSIVPCYIGNEVQLNTGKIIDKFFET

NWYELQPKQRKNMIVMQSILQERPIQLKVAGIFIINLDSFFILINSTYAIFAFMKNFKN

>OR40_CLUMA_CG005959-PA

MSNYVGDLKIKIQTLLNNNFDYKLEFQIIDKCFKISTFDPFKVRNGSWIYLSYVTSFWVF

FILITLKNIFVDFSGDNAKQVSSMVGAFMWIQNAVKFLVFCFCNKELLMLKKMILDDGKE

RCSDIERKYRRNNRILLWNMMKINDFVLAIEMKVPWTRPKSHPSHEINLAAFTLISLTSL

IFAIGCDSFSITVTTYGLSKMAICSDLASKIGRNTDDKFIEDLIKKHLHALDVIETAGNV

LQPINFCLLISDFMLIVLTIFQFKSGKGEPIAVVAAMCMTFLLFQFCFMGTAVSSSVRNI

HKFNLFTQMKYLQCEDFERSIYCSKWYELENVSLKKKILLLLNVAQRKREYSALGIKPIN

LYTFADVINKAYGLINFFLRRF

>OR41_CLUMA_CG003977-PA

MKFLKIKFMKTKFLKMELDFKIEFRFIEKLFQIMCKKNVKRSFTEKFYLIKVFPQLSPRF

KTQFVIYVAFWSIVHMKNIFVDFASNRVTMKILSFCLKLDSIRTLKSKILEDVADTTEEE

MKIERKYRKYARVLIFSTAIIYLFTSIISFLTALGFKSDTALPNTHSSHEINLMMTTFYQ

IIGVVIVVGCDSFFIVLTFHCVAKMDVCCSEASKIDGRTDKKFISNLVEKHFRVLEIIRI

SEEVLNPIYFHQLVSTFGVLVCTGFNIKAKADYLSSILLILSLFQFYFYCFLGNYVSSMC

EKFQSSIYCSKWYEIKDISTKKKILFMMMMAQRRKGFSILRIKPLNLETYADVMKQAYTF

LNILLGVL

>OR42_CLUMA_CG005956-PA

MDKISNLVRDLKIKTHTLLNNNLDYKLEFQIINKCFKISTFDPLKVRNGSWIYLSYVSIF

WVFFVLITLKNIFVDFSGDNAKQVSSVVGAFGWIQIVVKLLVFCFYNKELLMLKKMILDD

GKERCSDIERKYRRNNRIALLLLLYSLSTTAISYIVWNMMKINDFVMTIEMKVPWTRPNS

HPSHEINLAALTLITFTSVLSTIGCDSFLVIMTTYGLSKMAICSDLASKIGRNTDDKFIE

DLIKKHLHALDVIETAGNVLQPINFCIIISDFMLIVLTTFQYRSGKGEPIAVVAAMCMTF

LLFQFCFMGTAVSSSCEDFERSIYCSKWYELENVSLKKNILLLLNVAQRKREYSALGIKP

INLNTFADVINKAYGLINFFLRFV

>OR43_CLUMA_CG012053-PA

MINLPKTSRFIVEFDSTLDLFRKTVKTANIALWASGANIFKENHTFLTLQLFCTVSYIIA

TMAIIFYNLYLFHDDFIRCCFMLTIFFIAIQGFTKVYTLFRYKIGILAMVARCETIMMKY

NYPKVNEIFEKWMLITWYLFVLLAVTFCTAGVLAAIYPIIVYLITGTKFLHFGYQLPWLQ

WDGRYGYLCNFVFCCIATFLYALPLSATVCIYLLFVLIVLAQYDMLEVFIEELDESIVSN

DNGRNAKLIKSQIKRIAENYIEICDFVKAYNNTYQLVIFVEVGCLIGESTICLYANATDI

FFPGILIIFTCAFQIFLPCVLGTILIIAGDNFYDNLCKIHWNLLSIPDQHSFILLLISSK

TSEVITIQMNVLHLETFVAIYKSIYSYFTLLMSME

>OR44_CLUMA_CG010696-PA

MDLRTTNDTQSNCPKEKFSYKVMDKGKNWFTYHKLFLEICGMKPLKFSFKSERMKFLNYI

LTFLSYIFWHFIIIHLSVFQTYSVILNFDKALDKFIDIVINWMIYLYGYSILCFCQLNWK

KLSELLIFIEENFRQRSAKGLTFISIDESIKKVKKFILFWIFSCLSGTLMWVAYPLIQNP

CCKSLPIKTWYPVNVAEFPNYQIAYILQFFGQIYVAIGYGICGGLYVSIILLLCGQYDIL

YASLKNLDEFPDTEISNDSYLKDVVKPLTSLHEHREHRHLNEDEQECNQYFVSTEKYDDS

ENMLKFSSKAIKGRKTLSDCVEHHNMILHTAKMIENYFKWLILPRFTFTTLLLCTLAYVL

STMEEFSVGKIASLLIYMALSASELLLFTYSGELLKHHSCRGSEALMRTKWETFDATLRR

DMVLVQRIAAKPIIMTAGGMFYINMNQFRSVISSAFSVFTLLQNLKEKSS

>OR45_CLUMA_CG017364-PA

MSMWDFTIYHLKIIACQRRYIFKDKIWVIFNVFSYAFFGIPIFLFIIQSFKVITDVAEML

LPFVIEFNVFIKICTFYSNRETFYLLMEEMEEVRANRVNNFVVKYEMSRMEYYLNIFKVL

YSRILIFAGTSFWLAPIIHTCIMKKFLGHDDFQRYMPIKAIYPFDMYATPYYEFLYFYIS

YPIFLSIYGSGGVDIMFFEFCRALCDEFDIIQRAADTETEDKKILIGHHSKVLKVADQLV

ALMEPIIFVQFLIGSLTLCVVGFQLVMSDDFVQKFVVTFLGLSVTGQLFFYSLFAQLLAD

YSTTAADFSFALDTDYVLFIMRAQKSVVVKGGFYLATLETFNDIVSSAGSLIALMQSFVA

>OR46_CLUMA_CG015610-PA

MGMFSYIEWHLRFVCCHRPNSLKHKIWLAFNIIQFFCFFIPGLTFVIRSLDEITYAAEAF

LPNLIIINLCAKLAVFYFQKNRFYALMDGIENLRMNRSFKSETYNAINKMSRMEYLLNMF

KVLYSRFLRLTGITFSFTPLIHGTVMKILGHQDFERYLPIKAIYPFDMYASPYYEILYVV

MLYPVFITSYASVGFDLLFFDICRALCDEFDIIKHCVDSEDKKNIMSHHSKVLKIADEFK

ELFAPIIFIQFIEGSLALCVSGFQLVMSDVFAQKIVVTFLGVALMMQLFFYSLFSEFLKS

KSESAADLCYIPDRDYILFIMRSHRSVVIKGGFYYATLETFNNIVKTAGSLIALLQSFEE

>OR47_CLUMA_CG020270-PA

MNPLIGANLRAMKIFGYMIYDDQKYKRLHCFRGVLLTVSFIIFNISQFIDLFQHFTNIDE

ITKNAATTLLFATTSFRMINFYKNRQRYLAIIKYVDSEIKKMLSIEDNHEKEIIHSSIKY

MRNLTACFWIIALITGNLMCVNAAVQAFLHTSDKSPPLILRNWFPFNNFWLSYFIQYYIM

NIGMLIVPCWHSFIVSIMIFVITKLKILNKRLSGIDEEYSHFVECVKKRSDIYIFVKELT

SLISSSLFMDFIVFSILLCALLFQATQVDFGIQLAIIFFYIVTMTTILWMYYNHANEITF

YSNQLSISAYECNWYEHSRHFRKQILILITASKPIIIHAVFIIMKLDTFLSILRASYSYF

TLLSNIANDKALK

>OR48_CLUMA_CG016015-PA

MDIELFKPFKLIFKKFQWFGMWQDGEQTWAYFIFGYLLHLLLDGEQTWTYFVCGYLFHFV

FVYLYIFGQVIYLINSADLLDFAEAFVLTTTFVAQAFKSYNFFMKLKRIKRSMENINNLL

EKTDHETNFGRKLIRQEVSLCYKVYLIFWSSAIVTVISGAFAPVLSSKSPYKVWFPFDTN

KETNEVGFWISSYYLVFNSIPISTLDITLDTFPVIFMAFAIGFLRELSERLKKIRNNHEL

VQCVVIHKEVKLFIKDIHDNFAEAIFFQGIMSSLTLCTGVFTMSVTENLTQLIRIITFLI

PMLLEIFLPCYFGNELSIASSTLTTSIFQSNWIDGDMKFKRALMIFTECNRKELKITSLG

LFDVNIATFSSIGRSAYSVFNVLKQSTNRNSH

>OR49_CLUMA_CG005957-PA

MDKISNFVGDLKIKIHTLLNNNLDYKLEFQIIDKCFKISTFDPFKIRNGSWIYLSYVTIF

WVFFILITLKNIFVDFSGDNAKQVSSVVGAFGWIQIVVKLLVFCFYNKELLMLKKMILDD

GKERCSDIERKYRRNNRIALLLLIYSLTTTAISYIVWNMMKINDFVLAIEMKVPWTRPNS

HPSHEINLAALTLITFTSVLSTIGCDSFLVIMTTYGLSKMAICSDLASKIGRNTDDKFIE

DLIKKHLHALDVIETAGNVLQPINFCLIISDFLIFVLTIIQFKSGKGEPIAVVGAMCMTF

LLFQFCFMGTAVSSSCEDFERSIYCSKWYELKNVSLKKNILLLLNVAQRKREYSALGIKP

INLNTFADVMNKAYGLIKFFLSLV

>OR50_CLUMA_CG019937-PA

MKFLNNGLDFEREFQFIEKIYKIISLDIFGGSKNVSKRFLLQSAIYFLFWVVVYSKNVFV

DFVSNRAKQFAGIIGIFVFYQMIVKSYAICSQLDIVSKIKSAITEDANCTTEEEMQIEIK

YRKYSRILIVGTAFLSCSVMGLSVLWSLIFIKQMVMLINIQIPWTLPDTHPSHEINLIMM

FLWHFISTTLIAGFDSFFILVTFHCVAKMSVCCSKASRIDGKTDENFISDLVEKHLRVME

MIKISAKIFNQTCFHQLFTTFGVIVCSTFNFTNRFELFPFVMLSAAIFQLFFYCFQGNIL

SSMV

>OR51_CLUMA_CG017393-PA

MEAIEEIKLITMEDFTEMGIKFFKLIGLNLESRKEVTKKQKAFMLLGEFHFFLYLINIFL

VICGMLVYAYKNLHDIKIVARVLPNLTNAPYLAIKLFVFYWNRDKIKDALSILEESFPKT

EEDQLNLNVQTYLKEVKMFVKGFGFLIIVLNVVLIISQVVLIFMFGTTKLPLDIWLPFSY

ENFIIFGAVSLWMDWLCLVISVGAYAADIILFATISLTSILLCFIIFLLSSSRDISTFVL

YFPYLGTSLNQILLFCFLGQKIANSSSSVAIGIYNSKWHQIKSLKIRKALLMILIRSQKP

SALTLKKFGVMSFETFTKILAAAYSYFTLMKSFM

>OR52_CLUMA_CG004532-PA

MNTENYLICFKLPILILKTFGFWKEEPWSKLRWMYSILIRYLFLDCIVIAYSVHLIKSLG

IRSIADISTTLSYLLTIYGLFFKSMWIIIKQKKIRSMMKSLEELLKVTSFGKTNERNLMQ

HENNRMMKVERIFYGSSIVLAFFVLIKAIAFDSRIGKIPLDTWIFFNMEPNSFLYWLVVA

QECFATLFVIIVNFSLDMIQIVFMHYLTVMLKELSIEIESIQPEDDTKDYDRFCKCVDCF

VQLKDLGGEISTHLSLTFFVQAILSSVILCTSAFLLSTLSFTNEMPIIIRTVIYGISMMI

QIFLPCYYGNELTLISNKISTTLFHSKWLDGSKKYKKAVTIFMENAKRSINISAFGFVFV

DIGTLTTILNSTYSLYALVSKLNTK

>OR53_CLUMA_CG008545-PA

MKKLVQKLLFDRESTLNDKKLWQLSQNYSKFECFVIFCFDGMLILSGILLMFVTTFVRVN

EYRLPLNAYLIFLPIEGMSLNWAINYVFQLTTIGAAMIVFGSHTCVTLTIINQICFWIDV

VIVEVERLAELLNNPDFNYELQKTSTSDCIKNIIDSSNACSIWHREVQKLFQFFFLCQFT

VISIILSLNMVSIFINQMQSKFVFMAFFSFLLQFLLFCLMGTRIITKYETLCDAIYGTSW

FMIDVKSQKKVLFILMFCQDQRSINGVFYPVNLETFKKFSELNGIHFVTLKTFIITFF

>OR54_CLUMA_CG022056-PA

MELNIKKSFWFNFTCAKLFGFYGDTTFSKRKLRDFIFLSIVVFPCVASLMTFLTHRNLQT

FNDVSTIFVTFSCFGFSLIILMLKKKKFEKCLKTFHDLDALDEDEILKQNDNRIYILMFF

NVSCYIFTSVLYVIVTSIWRDEGSLIMPFPIDIKNDYLNIFMISFQAYIAVLAATINSAV

TTIIITFLAVYAANIDCLRIRIEKLSNEDIKQDLQICVEYHVKLMELFETIRNIFKESIF

YQSNVTVIILCTLTYQLTEISALENLAESIKIIFVASMMIFQIYLPHLFGSIIAEKSLKV

REAAYFSKWYDMNIEARRSLSIIMTQKEMEMDIMGFNKMNLEYFMTICNRTYSLYCLLKQ

FQ

>OR55_CLUMA_CG005942-PA

MTQIIKYIRDLKLKFDYLLNNNFDYLFEFEVIDKCFKFSTCDPFKIRNGSWIFLIYFTFL

WGFTIFTFMKNILKDFSGTDQLSAFVGTCVWLQTFTKLLVISFHLKKFSKLREIILSDGN

EIFDETEKAYRRTTRILVLVSIAIYSVCGSFLVLWNFVTIKDFVLTVEIRIPWTIPNSHP

SHEINMATISVMVVLTVFFYISFDSFFIMVTIHGLSKMSICSGLASKIGKNADDNFIDDL

VKKHLHALNVIETTSLILQPINFFLMLSDFGAIVSTLFQLKSGMKSVASFASLCLLVQFF

TYCYMGTKVSTACEEFEQSVYCSKWYELEKISLKKKVLMILNINRLEESEEF

>OR56_CLUMA_CG010584-PA

MDFFKGELNFKIEFNFIQNLFKIFSLDFFGGSENITRKFLIQAGIYFSFWSIVHVKNIFV

DFVDDRTKLFTAITGAFIFFQAIIFMKTVSFCTRLDSVKVLKAKILQDAAKTTKEEMKIE

RRYRKFTRVLIVATASIYFFTIVLALMWELIFIRQMVMMIRMQMPWTLPDEHPSHEINLF

MMFSWQVVTLIVLVGYESFFYLVTFHCVAKMSVCCFDAMKIDGKTDEKSISDLVNKHLHV

LELIEISAKIFNPICFHQLFTTFGLLVCSAFNRKSKFDILPLMIVMSGAFQFFYYCFLGN

ILSTMCEKFQESLYCSKWYEIESVKSKKKILFMLMMAQRRKEYSVLGVKPLNLQTYADVV

KQAYTFLNILLGAL

>OR57_CLUMA_CG007242-PA

MKLNSSSLKSSSKFYVFQVNGKLSKRFMLKAGIFFLFWAIVFIKNIFSDFAEDTKKQFSA

ILGFCLNCQTFLKLMALCNKLHCISAMRVHIVEDIKHSKPSEVEVELKYRRLIRRFIFSI

GFAYLITTVLMSFWSLICIQKMVMPFQVQVPLTKPDSHPFHEVNLAMIFLFLIVIIPLTV

GLDSMYVLVTLHCVSKLSLCCHLASKIDKKTDEKFILELVKKHLRAQEIIHESGCVFNIF

GFIQLASAFGFIVICFFNLGNNFDFISAIILSASLFQLFFHCYFGNILTSKFEEFQQSIY

CSNWYEIKNISIRKNILFMLKMTQQGKGYFALGIKSKPLNLRTYGEVIKQSYAFMNFLLK

IM

>OR58_CLUMA_CG020752-PA

MDRSQNPLVNIKKKFIALNIFDGVRMPSKRFFLTTTSYLIFWWIIHLKNICFDFVNDTKK

QFSAIYGFCAIFEFLLFKQISLKFIMTCSNLKFMSYLKNKILEDVRKTTEEDFEIEKIHR

KDGRKIIALYTIFFFSLPFLLSFGSFLCGFIIITRTQSWVYSHMGDTFAGLDSLYVSLTY

HCIGKLAVCSHLASNINQHTDEQYILKLIKKHLDVLDLIKVSAKFYNKLNSVQLCVSFGA

IATSMYNMKLNFEILLIVPLSAALIQLFFYFYVGHQLSSMNAQLQHSIYGSKWYEIKSIS

LKKKILFMLMMMQHDKGYSVMGLTTIMNFESYLDVMKQV

>OR59_CLUMA_CG020753-PA

XXFDFANDKKKQFSAIYGFCAIFEFLLFKQISFKFIMTCSNLKFMSHLKSKILEDVRKTT

EEDFEIEKMHRKNGRIIIISYTIFFLSLPFLLSLGSFLFRFFIITRTQSWVYGHMDGLDS

LYVLVTYHCIGKLAVCSHLASNINQHTDEQYILKLIKKHLDVLDLIKVSAKFYNKLNSVQ

LCVSFGAIATSMYNMKLNFEILLIVPLSAALIQLFFYFYVGHQLSSMNAQLQHSIYGSKW

YEIKSISLKKKILFMLMMMQHDKGYSVMGLKTIMNFESFSDVMKQVYGFLNFLLNVM

>OR60_CLUMA_CG020415-PA

MKFLKMELDFKIEFYFIEKLFKIFCEKNKLCRIILINEIFYSALNCFDGKRNISKRFIIQ

FAIYAAFWLIVHMKNIFVDFALNTTKQIAAICTAFLSYQVRFSLFKQTVTMKILSLCLKL

DSIKTLKSKILEDVADTTEEEMKIERKYRKYARVLIFFTAVIYLFTSIISFLTALGFKSD

TAMFVNIQIPWTIPNTHPSREINLMMTTFYQIIGVIIVVGCDSFFNVLTFHCVAKMDVCC

SEVSKINGKTDKKFISNLVEKHFRVLEIIRISEEVLNPICFHQLVTTFGVLVCTGFNIKA

KANYVSLVFLMSALFQFYFYCFLGNYVSSM

>OR61_CLUMA_CG019818-PA

MDRITKFLNNGLDFEKEFQFIEKIYKIISLDIFGGSKNVSKRFLLQSANYFLFWVVVHFK

NVFVDFVSDRAKQFAGIIGIFVFYQMFMKSFSICSQLDIVSKIKSAITEDAMNTIEEEMQ

IEIKYRKYSRILIVGSLFVYCSTMGISVLWSLIFIKQRVMITNIQIPWTLPDTHPSHEIN

LTMMFLWQFFAATMIAGFDSFFILVTFHCVAKMSVCCSKASRIDGKTDENFISDLVEKHL

RVMEMIRMSARIFNQTCFHQLFTTFGVIVCSTFNFINRFELFPFVNFGSIIFQLFFYCFL

GNILSSMCEKFQESIYCSKWYEIESISDRKKILFMLMMSQRRKEYSFFGIKPLNLETFAY

VVKQAYTFVNILFRLL

>OR62_CLUMA_CG014845-PA

MGFEGMNRILFLFGYHPFKFGDVWQRRWRLFLIGFLTFNFFYYILIIIYKIWFNNFTPTQ

LFGNFYYLINATKFFFFNHYFEKIISSLKEIMNLLCKDSSKKASENLKCFNYFSYAYLTF

YLILAISSFVIMAYDSKLSNENTQDAWPFETNMGIFQDVIILQTVTAICVTEIFGLCFQC

CYFNICMNIRMLYDKVLNEYQNLIFYHVISYKKELKDIFTSLQRIKLIINEINECFKMVI

FISFSVDVLTLGVFLSMIEDIHSIVTYFVYTPFVLYDVWVYCYGSSLMVEKIEEISSMIY

DKTWWFMLKSEKKMFRTLLQYTQRPLKIKVFIWDIDRELLILFIRFSYSVFNFVVMLKGK

KEKI

>OR63_CLUMA_CG007917-PA

MGFEGMNRILFLFGYHPFKFDDVWQRRWRQFLIGLSTFSFFYFIFIIIYKFLFNNYILTH

FLENCNYLINILKFFFFNHYFEKIISCLKAIMNLLCKDSSRKASENLKYFNYYSYVYLTL

YFILAISTSVVITFDSKLSDEYTREVWPFEANMGIIQDVIILQAVTSIYVGEIFRLCFHC

CYFNICMNIRMLHDKVLNEYQNLIFDHVISYKKELKDIFASLQRIKLIINDINECFKMVI

FISFSVDVLTLGAFVSVIEDFHIIMMYFMYTPFVLYDVWVYCYGSSLMVEKFIRFSYSIF

NFVVMLKGKKNEIRAPTK

>OR64_CLUMA_CG010679-PA

MKWKISDINRYFSLDSLSNIDIYHKIAGTVNSLGGMIGVPYLTTDFKFFSFHCILTIFNV

FGFFALSCYDFYLYRDDTNRCIFLIVTFLPAVQAIPLIYVFAYNHERMKNLLQRSEKFIK

NSDSITIEKVFHFWLIIGFKLLILTVVMFSFCCFLAFIFPIIYYFIMGEKILQFGFEIPI

IEWDSTLGYPLNFFYGAYLISVFYFALTSTLGYCTVCVLMALGQFSALKVMFEDLEEMIV

NNDDGSKHGAIKQQIKLIAEMHNELLEFLTCYDEWFQILFFITMGCMILQNTVTLYAIVS

FNFIIGYAFLVVTALQIFFPNLLGTVLIVAGDNYYNHLCNISWHMLSVSDQKSVLLLLVS

AINPKKITAGLAVLQLQTFIEIYKSIYSYFTLLTGGE

>OR65_CLUMA_CG010515-PA

MNFDLVKKLMGFFGIHPEKTKDSRQIIRRRFFLVILFFFNIFPMSYGTVWNILSHQNINA

YAEHIMVNAYNISGYMKYCMLIWNLEDYSKIFKTMTDLVPTRSMTKSDKNRKSLYRVIRS

YVLFLCTSTAIPFLMLTLTDEVFKDVLKYPFDFNFTPFKEVVHTMGFLTGMTFGALNFTT

HICFYSCCIQVITLYDYLTEQVLEVNTKPENEKEIIRNSVEFHMKTKEIIRDISSNFHGF

LLSDVLSYIIMLGTILFNLSYSLIFIRLATYSLSSFYGSWIFCNGGNILILQSQEMAVKL

YTEIHWPTINSPSLKRSLIIMIMNFQKPVQMILFKMRIVDLELFIFIVQTAYTLFNVMIK

LNY

>GR1_CLUMA_CG012846-PA

MLHVKSNIFTTLVSVDWHIDNLCDVLLLSQAMFGIFPICGLRHLNSAQLSFKYRSFSFIY

SMLIQCGIVLMFTTSIYKQLNSRIEYSKAVKFIFLFLNFLVYFNFMMVARKWPEYVTRWE

DAEQKLMKLNMMPHDQEHRIKQQIKKIIFFIMLIAFSKRFKSRITFSPTLVFLVEHNLGI

ASGIYSSKSCWSMQPADEAYFRNSFIDFFTVFNIFAWNFIDAYLIIISVLLTENFTCINR

KLNTNNTRIKSSQFWNDAWAAYKVSVNLVEETNDLNGGTILISFFSNLYFVCVQLLGCFK

SESTFVDGLHLWLSLIFLIGRTLAVCWFASQINDESMKPLHILRSVQSDYFDITMKRFSE

HLATYSVALSGMNFFQLTRKLILSISGTVVTYELVLVQFNEAEQEISDNNSCT

>GR2_CLUMA_CG012844-PA

MPYCSNIEQVSSMKGKLSRIHAVYCLMVQIFIVIMLSTLIYYFVKAEHFHYGKIIPVIFF

MNCFAISINLVYISWYLPSVFTSWSDFEAKYSEDDEKLTTKPWKFFSFFMTVAFLEHFVS

KVVDYEGASFCFHFHSTKFEAFSRGIIPDFFKVVPYSHLYGFYVVFTCFLSTVLWNFCDV

FLIIIFRLIHTKLKKFNEKISKKTFYHHDENFWHNARLNYEALHDQVKATNRVISCLVMI

SLLNDFYFVCNQALGAFKQTESIMQSIYFWFSFVFLISRTMAVLWNGSLVYQESKVIIRI

INNVPSEFYYREVKFFQHYAESETIAMSSFGIFDITKSSILEVR

>GR3_CLUMA_CG017939-PA

MPNLHVRFVLKSIDSIAKIFFLSPFKSYSINKQLPSWKRVTFWNVWTITGFLFYSIFHLS

YVSHNDQPTVRPKLVTVFIDMYNKYCGLILNCTLIIVGFFQQTNIANINMIFEEIEEFFL

VKLKIKIKNSKTLRFVYLQTIVIFSALIYTEMTNCLMYIGPTIGLSVDRACIVICLTPMM

TTAMVECQLFAYFLLMKERLSIINQSIYRYNNFHSDHNYIDRISSLEIIYTKLYEISDLI

NNAYGIQTIAIIAVQFITLTTMMYHCTMKVIRILSTELSDVENDETLEEVTSTVMWIAIY

MYKLFSLCYCIHATNDEGKKIGHYLHIFGLHSNHLGDKFKIKMFLLKLLQQRIKFTPYGL

FDIDLGLFSMITGAVVTYILILIQFDIAQKN

>GR4_CLUMA_CG019782-PA

MSDIFSEEMSVFIRIYQLFSLFPISLNPITNILLKIYSVFNILFVIGIFFSAFFVFPVLE

DSNSLSLLVGGLMFIGLLVTHVINLMQAFTSRHDQMMIYKKFDEIDHLLQNELLVNINYK

RLRQRLLAKYLLITFFVVAIQTTTIASSVVYNLYFRYFTHLILTIFIVRIRCIQNMFYVD

LIKEKLNLVNNKLDDTIRRNREKMAFAIYTNKLQRFDQRIEKRSSLYDQLMALKQVYGKI

WDISNLINDCFGWSLLAIVTQYFIEFTSNGYWLFLALENLLDRSIAIQSLCSIFPIVIIL

TTMAYSCHRCSENSQQTGVLIHKIERDINNDLQNALIREFSLQIIHEPIEISANGFFNIN

FTLLGSMSAATVTYLVILIQFQLSEIKKSGNVEN

>GR5_CLUMA_CG001975-PA

MEFPQETPVSKIYQDKVNGSLVLLEVFCLQHFSVKKLDDKTFKDCPSIFRLIYMICFLTL

VASVMVFYIIEDEGSLSIKEVQSKNFLAFAIKTTMNLGIFGVVCTNVIQSYLTTKNTKKI

HLTSMEMARILRDVLKCPVDFDRLTKFTMRRLLFTVCFFLINHCALILFKLDDRRLMSVV

VGSLPMFMLFLCAFKFNIYVWWINNQIQYLNMFLKTIFQKPVTKVISTINQKVFPINPQI

SYQNYMAQLLEARKMFNKIFSISSLLNQNQGMLILMMLTVLLVALTASIYDVFIIALGEM

KVQELPDKLHIISICSSLLLSIAIYCQKTQNVMCELATTLDVIQCEQFENIPTNVQIFLQ

SFYLQVNHQPVSFSAFGFYTINLTLLASIITGIVSYEIILVQFYAS

>GR6_CLUMA_CG001976-PA

MESQKKPVRPSMEEDLNNCVQIFEIVGLQFFSLKSIADKNFMDRPSTGRKVFVIVLIVVI

TFMTILFLSKNDRINTKNVTAKNVVMFSIRNFMNVGLIFVVCSSIIQSYLTTHKAKIYFK

NVKEISKILENEFGVVSNFKDIRNQTWKRFLLSFLFYNIVHITNGIFTKDLQDFLNKSLG

IIPLLYLWMFTFKFVFHVKLINHQLKTLQKMLQNIFKYQPLKIIDAVNIPQISIIKPMKP

SLDLLRHLQGLRKIYNIIYANSILVNECFGITILVLLCNIIIALTVCGYETFIFMLGELE

NSKTIETVSIMIMCISLLIALVFCCQRTHQILKRIGATLNEMECDYYDLLTSETREFLQK

FYLQLSHQPVIFTAFGFCNINLAMLGAIVIEIASYQIILVQFYAS

>GR7_CLUMA_CG001977-PA

MEKEINSLLSCFEAFGLQYFSLRKDDLHSRPSIWRTVYFAFILFLITVAFVTTAVISNVF

YLEEMSTKCLVLYAVRNFMIIGLILAIYTSIIQSYFSTRSTKKIFNNCKDIFDAFQVEFN

VNINLKDLKKRTWKKVAQAMMFLTATHSTMLALHENSSISIAEKFLSFIPMTFYISVVFK

YIFYVTTINFHLELLEKLLRNVAIKKEDKIMEKIIPGRNLRPVSVTSQEYIDPMKQFRAA

RKIYNLISENGDLVNKCHGFTILVLLLGIVLTLTGSGYRAFMIIMKNLSWRETPGVLYVL

VMTITILIAIVVHCNNTTQLIKKLATTVNKIECENFDYLKADVRQVLENFYSQLISQPVE

FTANDFYTINLALLGSIITGVMSYEVILVQFYAS

>GR8_CLUMA_CG001978-PA

MEKEINSLLSCFEAFGLQYFSLRKDDLHSRPSIWRTVYFAFLLFLITIAFVMAAVISDVF

YLEEASAKSFVLNTTRNFMIIGLILVVYTSIIQSYFSTRSTKKIFKNCKDIFDAFQMEFN

VKINLKDLKKQIQKKFIQAMMFLIVTCSTVLAIHESSLMNITERCLGFIPITFYIFVVFK

YIFYVTTINFHLELLEKLLRNVAIKKEYKMLEKVIPGRNLSRVSVTSQEYFDPMKQFRAA

RKIYNLISENGDLVNKCHGFTILVLLLGIVLTLTGSGYRAFMIIMKNLSWRETPGVLYVL

VMTITILIAIVVHCNNTTQLIKKLATTVNKIECENFDYLKTDIRQVLESFYSQLISQPVE

FTANDFYTINLALLGSIITSVTFYEIILVQFYAS

>GR9_CLUMA_CG001979-PA

MNSIIKYLSQCATIFQLFGHQYFSVKTLTVKNQNRYPTLGYIIYFIFIFLTLLIFSSIFV

STADIDTDDDTITPNTILSEVIAHSMLAGLVLIVIVGFVESFIATPKFKRIYLNFIQVAH

ICQNEFQFSINYRRIRNKMFLKCFVYFCLFVLCELLLFFYERSFNPKEALIQMSISFVPI

FFVIMSIFKFIHNVRLINLHLEILSKLMKNLFDARYKTIEHSNINKRQIRRSKYFNLSTR

LRFIKKIYNILHETSELINQSFGITVLLITLVMVISITESAYRIFLISVKKETIDAIGGA

VFNLLMGIHSLIIIVLTCDSSGKHMKKILECINNFETEHFQDIDDVSDELLTKLYMQINL

QPVEFTTAGFYTINLGFLASVNEVSVREVIKVN

>GR10_CLUMA_CG001980-PA

MNSAVKYLSQCATIFQFFSHQYFSVKTLSVENLSRQPTIGYKLYFIFIFSTLITFVTFYV

YLTKELLKSENLVLGAVINNLMFLSLILIILIGSIQSFFLTFTLKKIHLNFFKVSEICEN

EFQFIINYRSIRNKLFRNFLFYYFLYFSIYASYYVLQIIFKVPSTSFRFCAAFIPFNFML

MTIFKIIYFVKIINYHLEIIVVLTNKTFTSNYETSMEVDFYGKPMRHHKFICLTTKIREL

KKLYKILYDSSEFINGSVGVTILTIMLIVVALITASGYWLFIIIIRKESIDKVSGAMFAV

FTSFLTLIIITFNCSNCEKYMKDLLDSIDKFECESFYDIDEVSFDMLLKFYKQLSIEPMK

FTTAGLYNINLKLWSSELFPT

>GR11_CLUMA_CG001981-PA

MHSAVKYLSQCATIFQLFGHQYFSVKNLSDENLNRQPTIGYKIYFTLLFSLLVILSTIMV

ALLSADYKVDKITAKVIFSAAVTHSMLACLILIIVMSFVQSFTTTSKVKKIYLNFVRVAG

ICQNNFQFIIDYRQIRNKIYKTFLFHISFMFILLSSTYLYESSVGMPDAFVRIIAAVIPL

FFISMTVFKFIFYVKLINYHIEVLITLMSQTDKNLIQVSIINKKSLNPRLKLYGKINYLK

TIYDILYENSMLVNECFGTTILVVIINMVILLTTVGYQSFLIIVGENSINQSAGILFSAA

SSIFTFQSIVFCCDRCYKLINNFIACINNFECDRYQEIDYITNELLKRFYLQLIYQPIRF

TTGGFYTINLGLLTSALYHI

>GR12_CLUMA_CG022057-PA

MKQRSYDFEKSFNFFTMIVQIFGFQCFSVNNLSMNGSSLKFKFYSFIVFISILIASSYFV

IFKCNLFTNKKINSKNVLTYVIEYMAQGGGLVVIIVSFVQSIASTNITKRVYDNCFKINI

ICEREFEYSIKYLSTKKRFKMFLIFLSSYVIAALLNYFFLTNEPQINHFLCFLFILIQVV

IYKFILLVDLVNVHLRTICKLLHENDNIQFTSNDLILLSDVNQMHFNKTSLNSFKLLTIR

RIYNIINDNSELINQSFGYTILTIAVYTICVITSTLHDLILMIVGNLPVSRLGGIILLIL

VNYGILISIVCTCETTQRHISDFHVQLNKTELDKPKFKRQVMVKFMENFDLQLIYRPIYF

TAGGFYTLNLKFLATEQFRTK

>GR13_CLUMA_CG016160-PA

MTKVKIQKDIFYSSHYSFYVLKINGLAFYNFNRKLTKFEMNFMNYVHLSISIIFWLLLIY

LQFLVHDDSIIIPTSKVLSEVFKWQYRCEYFFSLLIIIFNFNKRSQIETFLKSLKTFDEK

LEELDWRFKVVQHKSLTFVACFILTTIIMAFHIILEYFISVDQGIVPDHSVIAVLNVLSD

LIVSQFLLLVSFQFISSVACVATRLTILIQNIKELLPLNEGEVREVQLNGAKEAFVLRNI

ALLHDKLLDAVDEINEVFSKEMIALFLLSTAIEVLVSYFVVKVSMLEILPIVKTWILINL

MIWSVLLVLPCIISIYISDKATNESRRLLNHVERYSSSYRDESALLKLDFLLNKLRCQPI

ILSCGMFNINWSFALSMSGAIITYIIILTQFENNV

>GR14_CLUMA_CG016771-PA

MTKVKIQKDIFYSSHYSFYVLKINGLAFYSFNRKLTKFEINFMNYIHFIIMVLFWIFLTC

LQFFGTAQEFLIVKTSKILSEVFIWQYRFEYFFAIFIIIFNFNKTSQIENFLKSLKSFDE

KLEELDWRIKIVHHKSLKFLICVVMASIIMMLHIALAYAMYAYFGIPSINTVVEVLKILS

DFIVCQFFFLVSLQFISSVACVVTRLTILTNNIKELLPLSEGQIKEVQHNRRIEAIILRN

IAILYDNLLDAVDQINEVFSNEMTFLFLLSTAVEVLVSYFMVKVSFLEMDAIVKTWISLN

FFIWSIFLILPSIFSIYISDKATTLSRHLLNHIEKYSSFCEDSNNSRDFQFQNIFSLLKL

NFLLNKLRCQPIMFSCRMFNINWSFALSMFGAITTYIIIITQLEHDV

>GR15_CLUMA_CG018783-PA

MHKNEKNIYHSNRTLFLFTKVFGAAPYQINKESLKIEMKVINHIFFVLSISAWVVVGIYR

TNVYLQVYEEAKETNSLASDQLWEITLTIQAIMLPFLVAFNVSKRQHIENLMISLKSFDD

CLKSLKWRFQIFHGRNYWIPSILIIPNLIYLISFEIGPHIYYHKDSEERSWFVLICDMIS

YLTSMELWLIFYLQFVVSVWCINTRFDALLKNFRFILRYDFCVKEFLNLGSTYNKDTLNK

ISLLYDILADIVEEVNSIFTKQVKFNKTVVKSVNYLILMLQLIPIFLIALLMETGTIHLI

IELTRGDVEEPIKWILINSFYWFVFLIAPSLVSIYVADGSSNKRSELSNLVENVSNSCDD

EDIFQEILSLKVKISNRPVIFTCGLFDINGELALSIFGTIASYLIIIIQFEQPAVA

>GR16_CLUMA_CG001423-PA

MFMKIIKIFQVAGIYPVRTFELTVYQSNVEEKRFYGNLSTFWSFFNLTIVLSLIIAIGMN

NQIFFYDESSIGNLSDILVYYSLLFAHLVIIIESLRKRKHLVKYWKFYTRSLRISKMFRQ

NIWIRKLFVKVLIHATITCLFELIIYIGITSDREWTLFWSAEVFSLMITRLHNLLHIFLI

DVIFFTLQDMNSRLKSDILWLKVIGEDKKFTKKFLYKNIKAIKTQFKNVMEMVLCVNRYF

RWTQVFNMGQTFIEITSELYWVYVFSVKVDTEFLWGKIVIIAIQYFPKLTTRCIKEAHEK

LAFNINHIDVFIDDRELRSLIMEFSLLIAQSPKYFTARNFFIIDYTLAGKMLSAVLTYLV

IFISFEPR

>GR17_CLUMA_CG007312-PA

MTSKFKNPFKDLMNPTDIYTAQWPLLKLSFFMGIVPYKIVNRENSRLQISVFGFTILILH

LILFGFCYIRTITVHESIVSYFFKTEISVLGDTLQLCIGLIGICTVFLYSVIQRHKFVAW

>GR18_CLUMA_CG006200-PA

MVESFTRVNVDKKKSRSPIVESLDFLFYLSRLLGVIPYSLSEYVSKKRFELSQFGNIFCL

VSMIHFIAHYQFLMGSSIFSGEMNNSSGTLTTVIGIFIIYLEPLMMAVDVLASLINQNRL

ISIFDRLKRIDDKLVKENISLNYQVIRKYSIIFIAIAAICEISLQAVYLISFDSDPFSWY

SLYWFVVTIPTFSNSVAKTWFLVLILLVQQRLRAINDYLNDTKRVLSERKMRNINTIGSN

HRKDNLFMENIEYLEKEFLSTRNAKIKSDNAWAVNSGMTNKVNDFNIFGQTKSKSIIKVR

PFALNREEAEAGGYKRSMHMNGWNVNDRDILIGDKMDEKLINLCRAHDEICEIAKEVNRM

FSLQMLLTMAYGFLLITAEFYFLYCGLLKQPVSTLFERTSNIPITISAIVYTAYKCVVVI

YFSWKTKVDSQKTGIQLHKIANVVDESHCYNVCNHLSLKLLNHHLNFTACGFFDLDMTTI

YAISGAITSYLIILIQFNLAAIRERSSSPIASLDSNALQWNNGTKVELSIPTFSPPTLDT

INP

>GR19_CLUMA_CG016769-PA

MRKYDVITVNKRKGIASYKDKDFMIIGLAPYEINKNSLNIEMKSFNYVQLLVSVFGWFAL

SVVKVRTLIFTFNNLFGLHSELLDHIRKLSFILIYIVTPFFPVFNYFKRHRIGNVLCFIR

NFDEKLHSLNWRFQIVHKRGFHFTFASIIPLTIAIVFIVQRVFISKLRNDFIINNISTEL

VLHILLYYLIMEVWWIIYLQFIGSVNCVNTRLDALQNNMSLILPNEPYEIFKVQKKDMQD

VGILYDLLADISEEINSVFTRQLIPLFAVALLFEAFFAFVTVKVLSTGIIFDSWIYSEFC

FWMLNLVFPSLLSIHVSVQASQKGRNLFIHLERVSNYIENKESLEKISALKKKIINRPII

FTCGLFNIDWTIALSIIAAITTYLTIITQFDK

>GR20_CLUMA_CG005394-PA

MSKVSICEVAIKVGSFCKFFGFAVFSVVDGKVKVTIFDKICFILNFLFIIALGYFGIYYQ

FSSFEDSILAAMGTCVAAAGGTLSSFFSMTITLYFRHDIWKLITLLDNMTNTFSKMNINP

NWKRFKVMCIVFIMSIVTFITTHFIVMKKLFEKIPRTSIMYSILFMKNSFSVGLTMIFSY

LLSLQISLINETLSLKSIHLDCIKCVQLINKIFGVLALGSVATDFLVSIFSNFVAFKAFY

FNDVNKTYAVECLCFGIFLKYTVMFMLYACNRVSSESKRLQTSISKLSFKEDYSILQLQA

FGNQVKNLPVKLTCGLFDFDYSLVMMMISSVCTYLLIVIQFEISAQQQSFKT

>GR21_CLUMA_CG018766-PA

MPTKKEENIFTVILPFYLIIKIFGLFPASFDGEPRLGIFQFKCRDRVSSLLSLIILFGVS

ILNILHPIEKLHKSFIITTGLFCGMILLIITMVYQLLKRHEIIKLLHILNDFDAKAKALE

VKLNLKASRKFILKTVMFILCGVLVVSIGTAFIFEMKIHYGAGCLMPLSYGYLLLYLSML

ILQFSFSTLAIKNRFCLLNDNLRFTFQNSLAKGNSTLANGNANRLHEQLSNTIRDLYSNL

CDGIDLVNESLTFQLIPFLVYYLTANLFAIYSTIREVFYQTPLMYIAIGTNICWMILHSV

IASIALYSGHITTKCALRTPIIVSSILRNCKDKESPFTKDAFKTFLLEVQHRNMFIENEF

FRIDWKLLFSMISTITTFLVITCQFDASLSNDSVIKNSTLG

>GR22_CLUMA_CG018785-PA

MKILTKSTSIYETFSPLSKVLQFVGIIPFRVNSESFEVSVKFCDVFWMFMIWILWLVFIV

YNFLQESHHGDDSVELLLMAWQWMQIFQTISCFCIQVINILRRNRIEKLLKLLHEVDEMN

SNMTFKEQHSKVKNLTLNTIIGSQIITFVIYSFTIFILMYKYNEKFHLDVVFEMQFYINF

TFFILTYHQFIFTLANIKSYFTLHHLSPYAGLFPQKTIETFGISFVRKVAIMHDKLNDAV

GIVNSTFSYEILPGMAYTSCLTISLAFGIFHTVYDEKNDDLLSNVIYFIWTWYMLAFLIA

AIFTADTTTKAANDTLKILSRVINNAYDELLLERLTIFSHQINHGTSVVSCGLFTIDWSL

AFSVRWFYLHSKS

>GR23_CLUMA_CG018789-PA

MMTEVTKMFSSNTEHIYDVAKPMHICSQLLGLTSFKVVKKNKVIVDSVTVFNVLILILST

FLYIFAIFLFCTNYIAGWKVKQVITSKIFECTIWWVAVCYFVTHIIMNWWFLFSRKHFIN

IFNKLNAVDENLSDLDVELKTTRHRNVIFWVSLITSTYFVLTTTLGYVASTNQDTLRIGI

AIYVIFFSVIYRNFFLNYHFIFIMWGIKIRYQKINSLMKEFLGHLNPSQNVEKGDEILNQ

IAKIHDKLVDVSEHINRTYGFPMLMGTATNFSYLTVGIFGIIRLLLHKTDQAAHAIIHIV

WMLFFSALIFANFYFGHVTNKEARKTANLLHKIGNKEFAIVSLTSVTKFSQQLMHRMPVF

SCGLFPFDGALAFKIVGAISLYLVILFQFENTEQK

>GR24_CLUMA_CG018792-PA

MLKANNICEVINPIHRCSQIIGLTSFSIKKDNHQNYEGFISFYDAMWIILSTVFRLFLIY

KHTSSNDFSMANREYLDQFYEYCNRVFLLLSILNELFIIWWLLLNRRKLVTVLTMINNID

NELIRLNCPLDHKSHKLTLIIYLVLSKLFNILFLLVLILSTHLTDIFKVHSMVYVMDVST

LEFYSLFCSQFMLLMWTVKERYKLINDYFITMMICQNSFELKNSKLRNVMRLNNQLIKIV

DDISFYYGLPMIMIIGVGFLVIILCTFVGIKISITENNWNHFSISFAAIFYNIILLSYFI

GIVSVGHWVKSEFQKTVSLVHKFLTNVKDEKIIKTFLDFSFQLLHRKPNFCCGLFSFDLK

LAFTMFGAAATYLVIAIQMDTAVSKHV

>GR25_CLUMA_CG018794-PA

MFSLKAKNIYEVARPLLNLSRYFGINAFTIEVKNERVTARVSWIDLTLILSANFNIIFSS

ICFLHVFSDLTKLDDMFYSKIYEKTFKCSYVFAFITSFVISWWTFSKRNSLIDLFNKLIE

ADAELENMKVPVNLKKVKKCILMFIFLGGFVISIAFVIYLPKIIAEDIHWIHHFGMISAQ

ATTFQIFLTFLQFQFIVHSIKARYVCLNRFLTDNFLKSSNNVYVGKAADLHKVTCIHESL

VECSDLVNKFYGVPAMLLMAQEFCYTITCIFVNIQFMYQNDSTFTFVSIPSLFWSANSTF

ILFIMTYWGHVARQEAMKTASLIHKIANRDKLNVLSPKILILSQQLLHRIPLFSCGLFDF

DLNLTFKMIGTAAIYVVILIQFDHAQIDMDDIEAKNVTLI

>GR26_CLUMA_CG018522-PA

MKFVPTIYQICAFDCVEGSLFSIPTVCEKIFVKIFVVLHVVYVILNLKFQYNFHLELFKH

PDVLGNITNLIEMILPLLCHFCIVVESFVKRRKERKIKMLMRKIRLNLNNTSRTNVSHFP

FVKFFFLFIVDTIIYIVVIVMLEEVPVWRLHMIYIFPSTVLVNMFDFYYATHVHFIEKSS

IMVQHKLIDTSISNAYHLSILTMLKETSFDLWHLTELINNRFAYEMLLSVVTKLAVFVID

IYWVYIRILHAVFNYHFIRIISCLYHYVAASLLCVHPLVSLIGIFYSCNKAVNEYKNISF

ALHQTSTYNIKSDFHFKKVQKFSLQLMSLKLQFTVKGFFEVTNRTLQEVNRFINSNLFKL

FSLKN

>GR27_CLUMA_CG012766-PA

MFLRQNNILKGNLAFMPLMLLLNLLGFYLKTTNKYKFFRMIHSVSFIILLLFVVSPSVQY

KDETSMVVSTITYINTRCIYYTLIIIYISNMIARISLSTIFKCLDNSDQRLQTIFDIKIK

DDESSWFIRIMIILLFISAFWSFVFECCSNKIEYNVGQFVHAMLVFTLSVKILFYCMLCA

SIKVRFIALIKHLKEIKSSQTSFVVNTTTTKYIAVTKSIEKLSQLKEVSLIYDEVLEIIL

LLNESFSTLLSFAFGTLFLFFTLNSFCTFFMMTTKNFTIFQLIEKAHWILLSAFAIFSLC

YTTESTNKAISDCASLLNTIYSHDDFENINHKVEIEIILTKIIIQMESFVFTCGYFDINM

KMLHSFVYTSITYLVILLQFNFEEVETSSPPTIAH

>GR28_CLUMA_CG000719-PA

MIKIGTTKTYSIWDSIRKLFKFVNFFGCAAFTIDGKIENGKIKTTLIDVLYFLLHLGFGV

MRCYWNIKYDLVFCTTNSVIIKIGTRLNSFISIISYLTSCIAIQFLMKDFWALNKKLSDI

DKEVNSMEKFNLRVNHKLVYYKSLILISIPLTIILTSGIYLCVLTKEFMEPENFIYYVPS

TLMNFAVINMIMVTFFFNTFLFYDRFKLINDCLKKYFKTDADSKFKNIKKLSTKFQIEMV

LKLAELHDNLTESLLFINENLGFTMLNITAFTVSGSIFSIFSLYRMIVRNELQYFNLALM

NNLCCGYFFIFLVVMIACCSLLTKTGKYAAILVHNVIAYIDDYDDPIIDYLKMFSQQLQH

RSPTVHCGFFAFDFTLLFMIVGTSTTYVVILIQFDSNGRAAHTDLMGNGNSTIF

>GR29_CLUMA_CG022063-PA

MITTSSLKKYSVWNTILVFYYITKFAGYIFFSIDGKPENAKIKTTFPDVVAYLAHMGAAI

FLFYITCTHDITFITTNNFIIDFCSRKVLQYMTLTLTISSFMMGARRCQIWENFQLLFNF

DNEMEHLSMRMNHKRTLLNFILFCLTCTLINLMLVAYTFYIRNDVKDPESHFYTALCTSM

IYLTLSMCFFGFFLIMYLAYERFDLINTCIERNFKTQEDKEDGIITVRKNQSNDTLKVIL

KLGELHDKLVDFIMIINSLFSILLINLFGNIFLSNVLSLFAMYRIFFGHDQENYYNGMIQ

FLWNVYFMTLTICFIALCSLMTRTGKSTAVLVHKAINYIDNDNDPIIFYLKMFSQQMFHR

SPTVSCGLFVVDFSLLFTNPVTEHVNDFLQILAATTTYIGRIYHKT

>GR30_CLUMA_CG022064-PA

MITVRSSKKYSIWNCFKMLHTYGKLSGFIFYSFDGKIENGKMKTTIFDMALFVISAVFCF

SLLYLHYTHDFLFMKTNNSIIDDASKGVLQFVILTIFMFNIKFYYNRKEVWISFKLNHKR

TCKIFLTFMLITSLIILSLILFTVYFTSYIFDSTLAVVVISITIMFNNGKIIASLLNFIC

DIYLFYERFDAINACLEKYFNVHDEENQRVEKKYHSKFLQKIVLKLAETHDSIINIVMDF

NRYFSMLVINFVGVIFIVNVFSLFSLYRVFFGNDHKNFYNAIIQSSWTFTVALYLLGFIS

ISSLMTRTGKYSAVLTHKAINFIEDDDDPIIFYLHRSPTISCGLFVVDFSLLFTILGATT

TYCVILIQFDSGVPMNGLGSNHTFNETSNDFYNISSLK

>GR31_CLUMA_CG007978-PA

MKFYESLKFVILGQNFYRLANFRVNKTYTKLVTDKKEIFATLIFILLCVLELCGIFYKFY

YAGDDSSFEIYDSTTGGFQLVTFIVYIELSLTVLTSFYFFFVSIFKRKQQIKFLEKVEKI

DETMRNNFNINIDYRMYSLNSLMSLIFTLIYYNIILSGILLKFLIQLKSWFGVVGFVTYI

VESGMSGIFTYGYISYVSLIQDRMWKLNEKLQVIIQKPPEELEKEYETKELLCNEMMRFT

KLYKNLCSCVEDINQIYGSSMVLFFAHDFTLLTTQIFGMFYISFFGDPQQLVYKLLALAV

WLLPNIIKMTIICFVCHFTRNMIETCGTYIRKFSNEAKEDDLADLVDMFSLQSIHLKTEF

SANNFFSIDMSLFFSIISACTTYLVILIQFKNYEDENVDVVLHENFTTTLSVN

>GR32_CLUMA_CG017731-PA

MKLFEKRNFYEVCAFTVGLTRIFGFFFFSVKSNSTESRKVKWPYISFIFFLTLGLISYLK

TVEMQFNSDSLIIHLGHKNLLPASIFMATFSIRIINFLMRNRHVKIIESIKSIDEDLEIH

GININHRKHFLTAFVSTILYFMIVNITLCWVIVLKLATTNDNNKYYWIINLFAAYSFNSF

LNNCISHMLITGSIYKRFQYVNEIISIKSFDTNLLKSIGKIHNKVTDTLSLVNSSFSLIL

IIYSTMTFIHCTFTSYQIFFYVRLPQFTDFGGIEMISSMLASIYFFFYASWMMIVSTGII

KEENKTKFLIHSKKILSDKNLLKNCEYFEMQMEHSKAEISSGLFTVDMNFLFSFISTVFS

YLIILIQFESIV

>GR33_CLUMA_CG017732-PA

IQSIDDNLHELGIDFNYRKQFFFALAVTGFVNILFCIFYYSLFKVYSMIEIEMGEKFLFF

SFILNLVVDAPSLNYSMCYLLLIAAIYKRFKYVDQLLTQSNVSLSTIKKLRRIHDKLLDT

LNLINSCFIIYVLCILGGVFITIVFNLYSLYHFLEANVQDAKSIIANFMMLFTLIFPLLQ

SLWIITYSKWIAEDNLKIRTFINAKLTRNQKFLESLNLFEMQLQHRKPRISVGLFDVDLK

FLFTFITALCSYLIILIQNENIFN

>GR34_CLUMA_CG017733-PA

MNIFKKKSFYEVCALNCGLARFFGYFFFSIGSGKAKNNEKWPYLHFLVFLALGLIGFIRG

KSTPVNSDSIIIYLGNTFLIYATTLIPNFFRIHNFLSRHRHVEIIDNINSIDDVLEKHGI

KTNHEKHFLTAIAVTVFHLLLLFITLFINYSIIQIYGKSNENDNQLNNFFYALSFFSHQS

FFLSNMLVTGAIYARYQNFNEILLHNKFDLTLLKELRKTHYKLADTLSLISSCFALNVTY

IFTMYSINCTFWLYQIYFSVTSKVLIEYDIFFLLLTTFYTLYFVGYPIWMIIFSNWIIKE

ENKTKFLIHSKKILSDKNLLKNCEYFEMQMEHSKAEISSGLFTVDMNFLFSFISTVFSYL

IILIQFESIV

>GR35_CLUMA_CG022065-PA

MKLFNKKNFTEVCSFNCGLASSFGFFFFSLDKQKTGCCQRCSNRCQKKCLCLNFLLVVIG

MVISYKRVDVDFLNSIESVVLRIGLKMYTQQFICIPLMMAFHNFLNRRRHVTIIENIQSI

DDNLHELGIDFNYRKQFFFALAVTGFFNSSLCIFYCWFYKVYSMIEIEKGGKFFFFNVIL

NVTADAPILNYLIAYLLLIAAIYKRFQYVDQLLTQSNVSLRAFRKLRRIHDKLLDTLNLI

NSCFVICVLCLLDAAFITLVFNLYSLYHFLEANVQDTKNIIANFMMLFILLFPFLLSVWI

ITYSKWIAEDNLKIRTFINSMLNRNQKFLESLNLFEMQLQHRKPRISVGLFDIDLKFLFT

FISALCSYLIILIQNESIF

>GR36_CLUMA_CG016636-PA

MAFVPYTIRSYEKYYKISKLLGYRTTTIRNGKTVLTLSDVLILILALSIGIGICFLGFSY

SSALVTSESDIASYGNYIATVSSIFISLSSMISLFIFRKRLWGIVLKFSEMDEKFYQLGY

PVNYTKQMNRINTFWILQTCLLVPFSVIVFYLEQSFIKILFRLYGSVYFCITIAGLTNSI

AGVNMRLKTSNEIVRINLNQNETKSKIIVEEKTKTDSIELISTLTEIYDILIDCCGDINT

CYGFVIMTSFGLCFFGILFTIFTACTHFLHHGNLSISIIISISFSIYYLFVLMSVIYTSA

RNKIEAQHLLKSLNVIMNHSKDPLKTASALSFSLLIKRRPPTVTCGLFDVDWKILFSMIS

SATTYLVILMQFESSAASKRHE

>GR37_CLUMA_CG001930-PA

MFLKHRLDWKFLFNFLLGLFTLSGFCPIDANAKRKGRKEIDFSRLLIILSLLHLAVAIIL

IFFACKAFYDNKFEVASFTNILKFSIMALTYAVAIIESLILRWNFVEIWARIRMIDVLIG

NMLPEFHSLLEIFYKKIVTKIFSYILLTVLLEIVIISNIKSDEAWTFMWIISIIPLSMSR

LRHLQHSLFIDSLTCRFKVVKKEMKTIVRLTKLEKNQSFSRNQSYYESVFNKISTIKKVY

NALWETSLCINRGFEMSQLLNLLQNFVQLTCDLYLLYSFLYNSNMTYLLELLFQLCPTMV

ILLIVLSSCEKCLDQVRYIGFVLHNIEKNIDDERINTLTENFSLQILHEPLIFTTGFFDM

DFMFLKTIIASITTYMVIFIQFMPKTEISTEALITANQTRAIKDHKNEYEIALMESHRHS

FESHIVPKLLCCYFSHKPELKGFVKCFPLIFFTH

>GR38_CLUMA_CG014388-PA

MRKNSQIVVDFTDQHDKVLNPHQRIFLEDTKRVKEKIEQLNHGNGLNSFMRKEEEASDPD

EMDKRDSFYHTTKSLLVLFQIMGVMPIQRSPKGTVPRTSFSYTSRVHFWAYFIYALETIV

VYTVAKERFIQFISKSDKKFDEIIYNVIFLSILAPHFLLPVASWRHGSEVAIFKNMWTSF

QLKYLKVTGTTIEFPILYRITWFLCFFSWGISFGLILSQFYLQPDFYFYHTFAYYHIIAM

LNGFCSLWYINCTAFGLTSRAFSLQLKATLTKNKPAKKLAEFRNMWVDLSHMMQQLGKAY

SNMYGIYCLIIFFTVIIASFGSLSEIIDHGFTFKELGLFMIVIYCMSLLFIICNEAHHAS

RRVGLKFQEILLNVNLTTVDIPTQKEIEMFLVAIEKNPPTMNLDGYANINRQLITSNISF

MATYLVVLMQFKLSLLRQTVKKSFDGSEDVNLNS

>GR39_CLUMA_CG011577-PA

MEPFVNFARFWGLWPQSLRRSSPGISIPSLLWYFVIVAIYIYVIYINLDKKFWMSFIEVL

NCDYQVYGTWLQILSSLMISLISILMTLFTFRKIWTLFEMIVDVDIILNKDFSLIVPKRV

HRTINYITVIILHVIYLVLLCVTYYIFNLTLGLDSYIICIDYLLANIPYLASMISFYLTT

NGISRRFHQVNCILLQLSPNDIPKDTFEVCSRSSKNDRHMPTIELNEIYSIYGNHLRKGS

PMSPPIKPLRDKNEISREIKKLTTKLENREENLWTKIHRRRVIEIEEYKMVKLINPDDII

EHLTKLLDIHAVLLDCIGLQNQIISLQILLIVAEIFIFEVYAMFSIYRTFNNTAIRSIPL

AYGNVFWVIFYSIMLYLIMSIATDCVKQGKNMGTCIHKVINKIGSHAHPKVIEKLSTMSH

QITMRSPDFNCGLFSFDWELIFSIISATTIYLVFLLQFDIAKNANDAEKI

>GR40_CLUMA_CG007501-PA

MTSRFTRFLLKYFYISSFMSGLVLVRFNFSKCCVERSYSGTALSVVWNLLFLTNYFRVCH

LAGWLHNLDNTLENTITAEIIAFFIMVTAIIVFQLLTFNSSKIFLTKLLRLIKASEVNYD

FLADSFGRQILFAFIFSEANHIFAYLYNISGWFHPNQMGEIFTLNPLNNSWLNNYFMSMP

TIFTIRLSTMVVSCIDIIRRFVQIMNSNIEKTLKIANNNNDKLRSEIDMVIQIYEDITDV

IRLFQQNYGPLILILQCYCMFVTINQLFYLYGFGLSFKNGSILFKLMLIVFAMLHSLQLL

LIAKAAKYLQHEGNRTKHLWYRFNFLPQNLPVAIEKSVEEMKLHMVLNPIAIELCGMFTL

NYFILYAVIATGAEYLVMLIQFDIGSSKFAKGLN

>GR41_CLUMA_CG007893-PA

MISYKVSNQSRFLSVFSVNFSVAFVSIKRMEMFCELLRQIFLDIFSLFGLGINRPIAFRS

IIHYLIFLVNVSIVITILIVYFLQGNIIVSSEPLSVTTDFIDLLFPISVHLLVLLSYTMN

QKRFHKICNLIENLDKSLKQFDLENFKIIEKRSLMSFTLKFFIVHGVGLGVNTIFMLISL

APGNPLWMIAIRTRAISLNILRLMSTQFMFYCDYLTSRFVIFNKECEKIIKTSDQFGTEI

EILQKIQLLKALDLKMMTLLHCFQKYFKTMLMLNITADVVAIVIAIYWIYGGLTYGNPFE

LQSDLVPCAKMIALYLVFHSGSSLLAERRKTATYIYLIKSKSLKFAVIKRHFLLQKLHTD

GEVFEFHGNGFFYVNFQTLVGVKSV

>GR42_CLUMA_CG016090-PA

MTARQFQNLLIFTNITSKLSGFMFISFHFKSKKLNCERNFHDKILFALSLFWSFLAFLLY

DGNFDLYGIVQSQIADNLVNIFVKSFNIIICILKIVNFVCSRSYSRIFLKLYDCQLEFER

NKISTVTKLQYIATSLCAIFHIIFMVILTIASYFCYKENGLYDGLKLKNFLLAFSALSFY

MMYYTSLLKLLSCIYCLINALNEYLEDNLKSPTVQSFSKITRKVSILNDKICDTLEVISR

NYLIYILNLVGCNLIISIVNFYNSYILLMSNGNIIFHIYFIEGICYLLFFTPFIISTTIL

SSWIESENMRTAELLNQPLMIKNDRNALIRLKNMSQQIEHRKPTLSCGIFIVNWKFMFSM

ICGIFNLAIVLIQFYDVKT

>GR43_CLUMA_CG017953-PA

MKYQTLFTLSSIMDVINDKDFDSALDHLNKDFHFLSTNRKDVNSFQSNDQRVKERHLRET

LQSRHGPTAEIHDQFYRDHKLLLVLFRVLAVMPIERSSPGRITFSWKSRASIYAFFFYTF

ATVIVVIVGLERIKILGETKKFDDTIYGILFIIFLIPHFWIPFVGWGVASEVARYKTSWG

AFQVRYFRVTGENLQFPRLQMIIVIISIGCLLCAVLFIVSLSFLLEGFPLWQTSAYYHTM

TMLNMNSALWYINSRGIKVASENLSRCFRKDVEIDCTAYMISKYRFLWLNLSELLQSLGN

AYARTYSTYCLFMLANITLALYGTMSSVIDHGFQFSFKEIGLIVDTVYCSILLFVFCDCS

HSSTLQVAQGVQDTLLTINLLVVDRPTQKEIDIFTQAIEMNPAIVSLRGYAEVNRELLTS

SIATIAIYLIVLIQFKLSLVQQQSRN

>GR44_CLUMA_CG015588-PA

MNQIYAFMPTLKVFQIFGINPFAVNKNFQPEESRNWKIYSIFCIAFSILPLSASWYDGKL

YLAEDKSDRGFIVDFIQLLGVRLTHLIIVFEAFYQQKLLLEYFVKLYELDMIIETDIGGV

KIERNRGKHLRMLMTAALFYVGVLLVSLVFLMKRDLTIYIYAISYLLSYLISCFRYLQYF

HCVWFIRQRLIILNAKFSEIEIDNKNIERKVKNLKFIKSVDVLLYKNDLKSQKYKKNFEL

LILMRKMYNKVYILSTLVNYSFGFSLLSNIANDFFSLTANSYFICLSMINSSELIEKIFN

GVRSFLWCSPHLLNLILASIVCHLTIRTSNKTGLILHKLKVDYNNEIQTLFIEQFSLQLL

HQKIQFNAFGFFNLDLSLLYTIVAAITTYLVILIQFYISGRDSS

>GR45_CLUMA_CG015611-PA

XXSSTEFLMILDSIGDKLENLPFSKTSMKNFKTYLKRTCTFFMSGMFIFYIMITIFYTYT

TKEEIDSKFMFEVLNYYGFGAYFNMILLFIGFLVIIIKNYLEMCHQNLHGYSESFGTFQN

KIKEVMTIQNDITQAIKLFNESFGFLYFGLYVYLFTNFATSLYFAYTSLIYDFNDSQAES

TLNILVNMIWILPFNIFNQLIGINCSQVQNKVFDIKRTLTKLYDDDHSGISGQLLSTVCE

DDFIFTANGFFPINRAIFMNIIISTSTFLIVAIQFYQQQ

>GR46_CLUMA_CG022066-PA

MQLIQLFENLKILIKILNFFGFFSVEMKPPWKNTWKWKIRATLMNSILAVGFSYTAIFNI

DAFSIFLNDKTYITWLCYIVEVVVIVFALFINTCGVFSATENSIEFLNILDSIGDKLEKL

PFSKTSMKSLKKYLKRSCSVFLSGMFIFFLLLTIFYSYYAKEFKSLRLILTTLNYMTFSI

YFNLSLLFIGFLVIIIKNYLEMCHQNLHGYSESFGTFQNKIKEVMAIQNDITHAIRLFNE

TFGFLYFGFFVYLFVTFTSEIYFAYASLLDDLRERQVEATIMLSINISWLLPFNIFNQLI

GINCSQVQNKVFDIKRTLTX

>GR47_CLUMA_CG011107-PA

MSLFFNTDDGAHIERLNSEQKKHGIFINVRPSRAWDDNGRTQSQHQTPTFIHRRNNLNIS

PGELKREAFTEVIPKKNIIFDCLKPIYNFLRALAVFPLSRRDDSDEFQFHIQSPAMVYSA

FVFVVVTIYSSYILMDRIVIVRTLEGRFEEAVIAYLFIVNLLPIIIVPLMWYESRKIAQI

LNSWSNFETIYFKTVGKSLQVDLKRKSLIISILLPILASGSVILTHYVIKTEDVQIGKAF

LLQVMPYCYLDTLTYMMGAYWYLACEVLTYTARILAEDFQKALRHIGPAAMVADYRSLWL

SLSKLTRDTGTATCYTFTFINLYLFFVITLSIYGLMAQVSEGLGIKDIGLAVTAFCSVLL

LFFICDEGHNASFNVKTIFQKKILMVELSWMNSDATTEINMFLRATEMNQANINLGGFFD

VNRNLFKSLLATMVTYLVVLLQFQISIPSEDAAVIKGNNSDYVA

>iGluR_AMPA_type_CLUMA_CG009342-PA

MLALLGILHKKKFLIYYCFYDDTLGAIFSEEQRDTSIELAFKYAVYKINKDRSILPKTTL

VYDIQYVPDSTDSFRTSKKVCKQIENGVTAIFGPPSDPLLSPHIQSICEALDIPHLETRF

DIDTGAKEFSINLYPQQQKLNEAILETVKYLNWTRYAIIYEGVFKQQDLMKSSERGMEVY

IRQALPETYRLVLREIRQKEIYKLIIDTSPQNINKFFRTILQLQMNDFRYHYMFTTFDIE

TFDLEDFKYNSVNITAFRLVDVESPKVMDIMEKMKKFQPNAKDASNGTSVIMSDSALMFD

AVTVYAHGLSELDSGHKLKLVNLSCDSEIPWNDGLSLFNYIDSVSLTGLTGKIEFNEGKR

VNFKLDLLKLKREELLKVGQWTSENGLEIKDPNAFYESNNTRITLTVMTREEKPYLMLKE

GNFTGNDRFEGFCIDLLKGIGQQVGFQYTIRLVPDNMYGVYNPETKSWNGIVRELIERRS

DLAVASMTINYARENVIDFTKPFMNLGIGILFKVPTSQPTRLFSFMNPLAIEIWLYVLAA

YTLVSFTLFVMARFSPYEWAKNFCHQEQVVENQFSVSNSFWFITGVFLRQSSGLNPKATS

TRIVGGIWWFFTLIIISSYTANLAAFLTVERMITPIENAADLADQQDISYGTLEGGSTMT

FFRDSKIGIYQKMWRFMESKKPSVFVATYEDGIKRVLEGDYAFLMESTMLDFAVQRDCNL

TQIGGLLDSKGYGIATPKGSIWRDRISLAILELQEKGTIQILYDKWWKNTGEICSRDEKN

KESKANALGVENIGGVFVVLLCGLAFAILVAILEFCWNSKKNAQNDRQSLCSEMAEELRF

AVRCHGSRQRPALKRNCTKCSPGAHTYVPSTFNLPPPHHYGQEIQYQNVSLPMMELKKYE

VDNNS

>iGluR_AMPA_type_CLUMA_CG009343-PA

MTDFRVLLSYLLHTISGAIFREDQKNSLHDLAFKFAIHKINRNQHILPRTTLIYDIQYVR

VDDSFHANKQVCSQFKNGSITIVSSEKALSNHIQSTTRALDIPHIQTHLDADSTDGNKEF

SINIFPSQTILNKAMFDVMRFLNWTRCAIIYEQDAGMLRDLLSFSPIDILVKTADQKSYH

GILQELKDKEIYNMIIDIHDTESMSDFLKAILELQMNDFKYHYLFTSFDLETFEMEDFRY

NFVNITAFRLVDTNDVFVKEILKEMEQFSRQHNVKFKRNLAVETQVSLIYDAVFVFAIGL

QTLQHSSEILFSNVSCKDEKALHSGSSLINFINTVEFKGISGQIEFKEGQRNAFKLDLIK

LKQNFFVKCGEWSHPTGLNITDYDSLFEGNQMNTTLVVVTILQVPYVMIHTGKNTTGNAR

FYGFCIDVLERISKIIGFNYILDLVHDRKYGAKDPISGDWNGEIFKTVNIKADLAVASMT

INYARESVIDFSKPFMNLGISILFKVPESEETKLFSFLNPLAVEIWIFVFFAYCLVSFTV

WIVARFSEWSVATKCNTNSGLYENHFTLSNSFWFVIGTLMQQGSDLNPKATSTRIVGGIW

WFFTLIIISSYTANLAAFLTVERMITPIENAEDLATQTEISYGTLESGSTMTFFRDSMIE

TYKKMWRTMENKKPSVFVPTYEEGIRRVLEGNYAFLMESTMLDYNVQRDCNLTQIGGLLD

TKGYGIATPKGSIWRDKISLAILELQEKGEIQMLYDKWWKNTEETCTRDEKHKDTKANSL

GVGNIGGVFVVLLCGLAFAVLVAIVEFCYKTKKDNFNDPLNLINSKNSLCAEMTDELCFA

LSCKNANHQRQAFKKTICNECRNSNNLFLKSQSNMINDIM

>iGluR_AMPA_type_CLUMA_CG021399-PA

MCSLRMFGAHGLLILIFIALGTNGQMLTEKIPLGAIFEQGTDEVQSAFKFAMLNHNLNVT

GRRFELQAYVDLISTADAFKLSRLICNQFSRGVFAMLGAVSPDSFDTLHSYSNTFQMPFV

TPWFPEKVLTPSSGFLDFAISMRPDYHQAIIDTIRYYGWDKITYLYDSHDGLLRLQQIYQ

GLNPGNETFQVEIVKRINNASEAIEFLQKIELLNRWSRKYVVLDCPTDMAKEIVKSHVRD

VSLGKRTYHYLLSGLIMDERWESEIIEYGAINITGFRILDTHKRYVKEFLDGWRKLDATT

SPGAGKEQISAQGALMYDSVFVLVEAFNRIIRKKPDQFRSYTMRRQNGQVNGTTKFLECN

TSKGWVTQWEHGERIAKNLRKVEIEGLTGDIRFNDEGKRINYTLHVVEMTVNSAMVKVAE

WSDVSGITPILAKYTRIRNHEIEKNKTYIVTSIIEEPYMMLKQQDLSGKVLDGNDKYEGY

CKELADLIAKQLDINYEIRIVKDGNYGSENPNVIGGWDGMVGELIRRESDIAIASMTITS

ERERVIDFSKPFMSLGISIMIKKPVRQRPNFFMFLNPLSKEIWVCVILSFLGVSIVLYIV

SRFSPQEWRPISAQTPPNNNFTMQTQTITTHQTTSLNEFSLLNSFWFAIASLMQQTCDFS

PRSLSGRIVGSVWWFFTLILISSYTANLAAYLTVERMVTPINSPEDLAAQTEVQYGTLYH

GSTWDFFRKSQISLYNKMWEFMNSRKHVFVHSYDEGIRRVRTSKGKYALLIESPKNEYIN

EREPCDTMKVGRNLDAKGFGIATPLGSPLRDPINLAVLHLKENGELAKLQNKWWFEKTEC

KGDKDNPTMSRNELSLTNLAGIFYILIGGLLVALAISLVEFCFRSQKSPTTTSLTSNTMQ

MTDTMKPKSRLPITSARDYDNGQYYPQSPSMEHPETHHINAHPQT

>iGluR_kainate_type_CLUMA_CG017925-PA

MITKVIVIVLICFKLDYTASLPDIIRIGGLFSPLPTDNDHQTTDPQEIAFRYAVDKINAD

NKILPRSMLQAEIERILPQDSFHASKRVCHLMKTGVAAIFGPQSSHTASHVQSICDTMEI

PHLETRWDYRLRRESCLVNLYPHPSTLSKAYVDLVNAWKWKSFTIIYETNEGLVRLQELL

KAHGPSEFPITVRQLSDSGDYRPLLKQIKNSAESKIVLDCSTEKIYEVLKQAQQIGMMSD

YHSYLITSLDLHTVNLEPFQYGGTNITAFRLVDPNNSLVRQTVKNWTSGDTKNGKKVNFN

HDNSSVIKAETALMYDAVHLFAKALHDLDSSQQIDIHPLSCDNQDTWPHGYSLINYMKIV

EMRGLTDVIKFDHQGFRTDFVLDIIELSPSGLRKCGTWNSTQGVNLTRTYIDQQQEIKEI

LANKTLVVSTILSAPYCMRKQSFEKLAGNAQFEGYAIDLIQEISEILRFNYTIRLAPDGR

YGGYNSDTHEWDGMIKELLDQRADVAIADLTITYNREQVVDFTMPFMNLGISVLYRKPIK

QPPNLFSFLSPLSLDVWCYMATAYLGVSVLLFILARFTPYEWPAPEQPVQPETQFTLINC

LWFMIGSLMQQGCDFLPKAVSTRVVAGMWWFFTLIMISSYTANLAAFLTVERMDSPIESA

EDLAKQTKIKYGALKGGSTVSFFRDSNFSTYQKMWAAMEAQRPTVFTASNQEGVERVIKG

QGSYAFLMESTSIEYTIERICDLTQVGGMLDSKGYGIAMRQGSPYRTLISGAVLKLQEEG

RLHVLKTRWWKEKRGGGACKDDTSKSSSTANELGLANVGGVFVVLMGGMGIACVIAVCEF

VWKSRKVAIEEK

>iGluR_kainate_type_CLUMA_CG017927-PA

MLTWLLILNNLVLILSKRQIHIGGMFHEHNYESEIAFRHSIERVNMHERTFELVPLIYHV

SPTDSFKVERIEGVAAIFGPSSSETSGIVASIAESVEVPHLTAFWERESTGGEGKPTQMT

LNMYPDNEVLSRAYAELLIDYTWKSYTIIYENDDGLIRLKDVLQIHDPQSPPVTVRQLED

GPDYRPLLKEIQSSGESHIILDVSPDKIVTLLRQAAEVKMMEEYQSYIITSLETHTLDYE

ELKFMRANITAMRMIDPTSFEVMNAINDWEQGERQHRRNFHITPEKVLTETALYHDSVRL

FATAVQELESAEDADKIEAKKMNCKHPTPWEQGSQLVNYMRLKADKGISGEILFNEQGKR

SHFHLEITELSKEGFKKIGTWDPVHGVNYTRTLGEAYDQIVESLQNKTFIVASRIGAPFL

MWREVENGVIYEGNHQWEGFSMDLIDAISKILHFHYRFELVPDGKYGSYNKVTKQWDGLV

KHLLDRKADLAICDLTITYERRTAVDFTMPFMTLVNLCLIFDAFLILNMTGISILFSKPV

KQPPDLFSFLSPLSVEVWVFMLAAYLGVSVMLFVLARIAPDDWEASHPCNQEAEELENIW

NMHNCIWLTMGSIMGQGCDILPKGLSTRIITGMWWFFALIMLASYTANLAAFLTMERMDA

TIDNAEDLAKQSRIKYGAVKGGSTMKFFQDSNFSTYQRMWATMESTRPSVFTTSNDEGRE

RVAKSKRQYAFLMESTSLEYITERSCDLTQIGGLLDSKGYGIALPLNSPYRTAISGAVLK

LQEEGKLSTLKEKWWKGGKCRDESSSGGGDDAAELGIANVGGVFLVLGFGCLSALIVAII

EFLWNVKKVAVEEKLTPWEALKAELLFAVNISIVTKPVHNRLSESESSKSTRRSQRFGSE

TRSTLRDKSERSVNINNESPQFSSRSLTGLNRIGNLFGKKNSD

>iGluR_kainate_type_CLUMA_CG022092-PA

MFFGKWIFITFFIFSHSFVNARKENSGLFDDESHDAELAFKFAVQAINNQRNKQTDGFLE

AVSRRIRYGNAFQASQELCRLMKNGISGIVHGPLSSKAAVHVQSICDTKEMPLLETRYDP

FTEQPIINLHPHPKVMSKLFLDLVNAWEWDSFTIVYESSPWLPLMNGLLKMYDKHGFTVT

VRELDITGNQNYRHQLRRVKHSGDKNIIISSSIKALPEILKQAQQVGLLTDLHQFIITSF

DMHTIDLEPFQHSGANITGVRMIFPDNPMVVHVTEFFKQKYFDRLEKEKYKSINNIEDDD

NPNDDYDSEADQEDENDNEMDEVPSGLTADNMRLDTALTYDAVLVFSEVVKRECGIRNKN

IKCDDDDGLRVHGFSDLMAMKTIPSIKGLSGDIHFDQKGYRSNFQVEIIELASDGIRRIG

MWNTTKGISLARALPNVDDSELSLRNKTFLVLTALSAPYGMLRETHLALTGNDRFEGFGI

ELIQKLSEKLGFNYTFKLQEDGAYGSLNKETGEWNGMIRELMDDRADLAITDLTITSDRE

GAADFTMPFMNLGISILFEKPKKEPPELFSFMSPFSSEVWMYLGTALFLVSLSFFVMGRL

SPAEWDNPYPCIEEPEVLENQFSFRNSVWFSIGALLQQGSEIAPKAPSTRIVASMWWFFT

LIMVSSYTANLAAFLTIESLSSPISNVEDLANANGAIPYGAKRGGSTFGFFKESENPIYQ

KMYEFMSNHPEHMTGTNDEGLTRAKAGKYAFLMESSSIEYIIERNCEVTQVGGELDAKGY

GLAMKKNSPYRGALSEAVLQMQESGEITQMKIKWWKEKRGGGACTDVAADSGAEELKVAN

VGGVFVVLISGGGVAIIVCLFEMLIDIRNRSKELETSFLQELISEVKFIAKCSGNTKIVK

HKKSPSRDESTEHDSRSASRSTYSPNRELTTADVYSFHQPSLKSLEKLDLMSEDNEISER

NHERV

>iGluR_kainate_type_CLUMA_CG022093-PA

MKFELKFLIILFNFVSQFSEAAKIGGIFDDTQEAEIVYKYAIMSMNNQRDRHTVQVLGTS

KRIVYGSEFAAAQTVCKMIENGAVGIIGPSSPESAVHVRSICDSKEIPLIETEIDGSSRH

VINLHPTPEDLGRAYLDMIHSWDWQGFTILYQDAPWLSFVEYILKNYKMDKTITVRQLDI

TTNNNYRPQLRRVKQSNDKNIVLCSSIEALPEILKQAQQVGLMTDEHQFIVTSLDMHTID

LEPFQYGGTNITGFRLVSPTDPMVIQVTDFFKEMYLGGSGDRIEPQQEQQESYYPANDPS

ARSEEVPAGLTAEGLQLKTALTYDGIMLFSQVMSQHRGIYSGSILCDDIESSFVNGTSIF

NSMKTVPPFKGLSGEIQFDQHGNRENFQLEILELATDGLKQIGTWNSTKGIQALPGNQIE

IINSNGNPFKNKTLIVLTVINEPYGMLKETPLQLTGNSQFEGFGIELIEKLSARLGFNFT

FKLQEDKKYGSLNKETGEWDGMLRELIDGRADLAITDLTITAERESGADFTMPFMNLGIS

ILYEKPKKEDPELFAFTQPFSGEVWICLVACFFLVSVSLFVFGRLSPAEWDNPYPCIEEP

EVLENQFSFKNSMWFSIGALLQQGSEIAPKAPSTRIVASLWWFFTLIMVSSYTANLAAFL

TIENPSTVIEGVEDLVAGKVMYGAKETGSTLSFFRDSESDDYKEMYKYMVDHPELMMATN

EKGLERAEKGKYAFLMESSTIEYNTQRKCSLVQVGGKLDEKGYGIAMQKNSPYRGPLSEH

ILNMMENGEISRMKTKWWKEKRGGGACESPDDAQVEELSMRHVGGVFVVLAGGSSFALIM

GIFQWLVFIKKASKELDVPFMDVLREEFRFFRRFGTNVKTVVTRKSSSQLDSISASNGSR

SRSSGNVQNDSHPISLRMSLKNINKIAQDEDDFETN

>iGluR_kainate_type_CLUMA_CG022137-PA

MKFYRHFIVFVLFCIIEVKSQPKRVVTVGGIFHGDDDISQIAFKHAIERINSRSTLYQIV

PMMFNISRTDSFKAQTIVCQLASEGVDVIFGPSSVETSGIVASIAEKFEIPHIIFHWKTK

PLYWEKSVEHTMTLNLYPDSNALAEAYGNVLSGYSWKSYTIVYENKESLIRLKDVLQVHD

SSSLKIEIRKLDDRYGSILKEIRARGDVNIVLDIGPDKIVPFLTEAASVKMLGDYNNYFI

TNLDTHTLDISQVQNRTANITCIRLVDINSDELINALRVWRQREYNFNMDEKQVPHEAAL

VHDAVQLYFNGLQSIGHNYRFQKTTHNCSEPRSGSKSNFGFQLINFLKNQEFDGATGKVV

FNNILPNKGGRTEFRIEILEMVKDKFISIGHWDTTEKVVVNRIEEELDMQRTEAMHNKTF

KIISKWGEPFLMPAEAPDGTILEGNARFKGYVPRLIKELQKNMGFKFVLEVVPDGEYGSL

NKETKKWNGLVKHLLDRKADLAVADLTITYERKTAVDFTMPFMGLGIGILFVKPAKKETN

LFSFLDPFDINVWKFTGAAYLSVSVLIFILSRINRDDWEPAHPCMQEPEEVESIWNILNC

VWLAMGSIMGQGCDILPKGSTTRIVTGMWYFFALIMLASYTANLAAFLTMDRMDKTIGSA

EDLAKQVKIKYGAVKGGSTMRFFQESNFSTYQRMWAAMETNGDDSHTTSNQEGVARVLKD

REKYAFMMESVPMQYAINKECDLKQVGELLDSKGYGIALPMDSPWRKAFSEQILALSEKG

VLDQLKREFWKKKNDCGETKTKDELGIENVGGVFYVLAYGCLIAFIVALAEFLWNVEKIA

VQEKITPWDALKSELAFVLKFFITTKPVRSKSSELISGKSSPPRSLKTRSRYGSESRSLK

NKSTRSLIYDDSNSKSLLNLN

>iGluR_NMDA_type_CLUMA_CG005272-PA

MSSTMRHTMSEFYLWILILFTLNNSVLLQKPSGENPTFFNIGGVLSNGESEKNFSETIAT

LNYVHQYVPKGTTYFDKTIRIDKNPIKTALNVCKHLISKRVYAVVVSHEEKDSSGHDLSP

AAVSYTSGFYQIPVIGISSRDAAFSDKNIHVSFLRTVPPYYHQADVWLEMLSHFGYTKVI

IIHSSDTDGRAILGRFQTTSQTNYDDVDVRANVESIVEFEPKLESFVEHLVELKTAQSRV

YLLYANKEDATVIFRDAAIFNMTDAGHVWIVTEQALFSNNTPVGALGLKLNNDNETEHIR

DSVYVLASALKEMMVNETITEAPKDCDDSGVFWETGKKLFHYLKSRNINGKTGRVAFDDS

GDRINAAYDVINIGKKGGMKIVGSFFYDNEREKMRLKINDSEIIWPGKVKKKPEGFFIPT

HLKVLTIEEKPFVYVRKLTDDEIDCEEDEIPCPHFNVTDGNEKDFCCRGYCIDLLKALSH

RINFTYDLALSPDGQFGHYILKNLSSTLGVKKEWTGLIGELVSERADLIVAPLTINPERA

EFIEFSKPFKYQGITILEKKPSRSSTLVSFLQPFSNTLWILVMVSVHVVALVLYLLDRFS

PFGRFKLTNNDGTEEDALNLSSAIWFAWGVLLNSGIGEGTPRSFSARVLGMVWAGFAMII

VASYTANLAAFLVLERPKTKLSGINDARLRNTMENLTCGTVKGSAVDMYFRRQVELSNMY

RSMETNNKATAEQAIQEVKNGGLMAFIWDSSRLEYEAAKDCELVTAGELFGRSGYGIGLQ

KGSPWTDAVTLAILDFHESGFMESLDREWIFHGNVQQCEQFEKTPNTLGLKNMAGGFILV

GAGIVGGIGLIIIEVVYKKHQIKKQKRLEIARHAADKWRGTIEKRKHLRQSMAMQRQYNI

GLNSVTKSISQVVDKNRYPTLTHRGPERAWNTGALSAQDLSGRRNGEEFIFNNQRHKKPP

KYKPPIPTFASDVSHLVV

>iGluR_NMDA_type_CLUMA_CG008543-PA

MPLLFTVQTNSLLLLLLIWAFVSGELKDYNEPKVEPLLQSNARPSGQSISFSPGQSVSEL

PIFPERSDAVYFVVAVPGGAKSWGRALARTLLDMGTPFSSPQGPPLRPIYVDLPTSGRFS

AKVLTTLCDQVEGVPLSGMIVVGDGQAARSIALSGNAMKVPVLWAKGGTAQLHAGGSEAQ

NFLQATLQPSAKEILQAIRAIFLQTHWHSFFVLSDIGSTMVLGSSLGGILKKTPLSPTIL

PLSSNKDDVFRQLAKISRSTRGIVLLLCDLNAARSVMSEAQRLKMTGGHFIWIWADTSST

AEFFQPNSVPNTDDKDQMMNAKGNYEEFNNRKKAESQIERHQGGGGSKRQQNNTRYKSSN

NNNRFQQIIGLNEDDERTFFTDGQVPLKVKRFKQNLESNDYTGSVEKHSQNNLKIKNIES

KEFNANYYDPYSIGRQGEDDDNDSSYGSDLNESTNIENNYETFDKINPYESHREVTTSAG

FEETTRRPKSSDVNEKTQANNNKNTNRNHRNDGNNNKPITPTVSAPKIFDDNENLDLEVY

TEASNDLKAKRADNSPSTFNISSHVFFHHFKDFPVGLLALRHIKMNIDRVFVRAAVRLFA

STWNIVEKDEELRTASGGKFIFQKINWDDDWSDNDYDDDEIVNNFKATKKLKSKGNQNVN

NRHMNARGSRKYKRDVQLNENIEASVIQTTFVNSRNKSSNSNTKVVDDLKNKTNLQHVSD

LNSHPSDDVNKSNKNNKNITSNSNTPSAVNVHRETIQSLKLDGRIDIVKRQNSWWSNIFG

GKTPEKPKITRGTPHYKGGCFGVPTRADVNRSEIFARYLREAVTLALSGRTLPGGTMEKN

LISNFEILNLVPTQSKARMQQQHQRKKNELKKNSNLMSSKSENPESTKWRRVGLVLGRKV

HLDTIVWPGGDIVVSGLSTRARSVFRVVTSLSPPFVMESNLDEDGMCLRGLPCHRLSTSG

KHNLTLMFNSIETKERFEEDAVEHGNQIPLNEDHKSSEKVSYKTRCCYGLSMDLLDNVAS

ELGFGYIIYIVSDELFGSKQVKKNFQPEFNRPVDAVQEHLRDRDRERDKNINEKSNRRFN

QRFHDQSVWNGIVGDLVVGSADMSFAPLSVSKSRAEVIDFSAPYFHSGVSLLAAPKVTTD

IPLLAFLMPFSPELWIAIFIWLNLTAVAVAIYEWLSPFGLNPWGRQRSKNFSLSSALWVM

WGLLCGHLVAFKAPKSWPNKFLINVWGGFSVIFIASYTANIAALIAGLFFHNAANTYDTS

LMNQRVGIPRSSAAESYVQHNDKHLWEKMKKYTFSSIEDGIIGLKNGSIDLLMADSPILD

YYRGSDLGCNLRKIGENYVEDTYAIGMSKGFPLKESISALIAKYSSNGYLDILVEKWHGG

LPCYRDEDHIEIVQPRPLGVAAVAGVYLMLGLGMILGVLILIFEHMFFKYYLPILRHQPK

GSVWRSRNIMFFSQKLYRFINCVELVSPHHAARELVHTLRKGHITSLFQKSVKRKEDEQR

RRRKSKAQFFEMIQEIRRVQQEEKEQPVVPTLAVVPEVEPNQSPNPEIQSIASSVSSVKP

KASPKLRIFSLKRDGRSRSNSSSLNVRRFSTDSIMGERLDTIGRRLSRDIASDLANSPPD

LGHRFETFGKTETTSKFDTFSGKGLTKSADDLDKSSREKNKFDTFSGVFDEAKPALPAKK

NPRNKLKRKQISKEIFESKHQVTFENQSTLPLRDSAREVIKESYRESLHPAIFQLDSSKA

VLRDKLHEELKQKYGKNKTLLKAKPPKAMSSSVQRSQIKSQNPNDSFLKVQSNDSLNVRP

VPSPRSKSRNYSEDDDDDEGLDPTYATIQPRNKSPRVSNNSVPLGRLSKEDLLNLSSRTE

SEIHEFLNGKPGTKNSKTDPP

>iGluR_NMDA_type_CLUMA_CG014919-PA

MRLLVLVTILMLINLSVGFRGNSNRGSSSISSSQSNQLNIGLIAPHTNFGKREYLRAINS

AVTGLSKARGQKLTFLKDYEFNPSNIHFEALTLNPSPTAILNILCKQFLHANVSAILYMM

NYEQYGRSTASAQYFLQLAGYLGIPVISWNADNSGLERRASQSTLQLQLAPSIEHQAAAM

LSILERYKWHQFSLVTSQIAGHDDFVQAVREHVVDIQDRFKFTILNSIIVTRPSDLVDLV

NSEARVMLLYCTKSEAIDILKAAKELHITGENYVWVVTQSVIENNQPHSQFPVGMLGVHF

DTTSASLVNEISTAIKVYASGVEYFLNDPSNRGKKLNTHGLSCEDEGRGRWDTGEIFFRY

LRNVSIEGETNKPNIEFTTDGDLKSAELKIMNLRPSVNSKGIVWEEIGVWKSWPTHSQQK

LDIRDIYWPGNSHAPPQGVPEKFHLKITFLEEAPYINLSPADPISGKCLMDRGVLCRVAA

DPDITDIDQSHKNGSFHQCCSGFCIDLLEKFAEELGFTYELVRVEDGRWGTLENGKWNGL

IAELVNRKTDMVLTSLMINAEREAVVDFSEPFMETGIAIVVAKRTGIISPTAFLEPFDTA

SWMLVGVVAIQAATFMIFLFEFLSPSGFDMKLTVQTNSNGVLPYRFSLFRTYWLVWAVLF

QAAVHVDSPRGFTARFMTNVWAMFAVVFLAIYTANLAAFMITREEFHEFTGLDDTRLSRP

FSHKPPIKFGTIPWSHSDSTMAKYFKEMHYYMKKFNKTSINEGVAAVLAGNLDAFIYDGT

VLDYLVQQDEDCRLLTVGQWYAMTGYGLAFSRNSKYVQMFNKRLLEFRANGDLERLRRYW

MTGNCRSGKQEHKSSDPLALEQFLSAFLLLMAGVLLAALLLLLEHLYFKYFRARLAKKDR

GGCCALISLSMGKSLTFRGAVYEAQELIKNHRCRDPLCDTHLWKVKHELDMTRLRVRQLE

KAMDSHGLKPPQIRLSSMSNLIAERKEKANILGNLSLGGSAQDLYRWTYKTEIAEMETVL

>IR_CLUMA_CG001153-PA

MLIKNVSILNKILIFFVIINSSYGELKPLAYESDLAFTPCLINFTQKYFHSERALRGSLV

IINFGKHPEGFFLLKILEALNEDKNHTFGLMPKDGRWHDDPAKVIDKAQNYMLIMLDMDD

LRLNVDQWKSLPTWNPLAQTIIVLLKTFDTIKEKDEFVRRVFEELLADGILFVNILFRMT

STLNKMAVETWFPYYDKGCARFVDNIYKIGECIVSEKMNRLTGISEKTWKIYEFNEEKYP

KIPKIFNNCPLIISTFVWEPFVVGNEKIETGLEILMIKTIAQQMDIKLQFNIINDDLVTA

KISGDNQTGIYSDVLQKKADLMLGGLYENPISRKLVSPSIPYYQDDITWCVAKAKFAQTW

LNVFIIFNLTTWFIIIFTLVASSVFLYAIAYFTKQTKENFTWVFLITVTLSTGQYGHYWP

NRSSIRFFLIILYFYGLHINTAYHSSLINVLTNPRYEDQIKTMEMAIEAGLTFEVNVNTM

EFFNSKGDWISKYLLSNHKLCRNIDKCFKKIKKDRKVAVALSRAHALNNPFRFIEDEDFF

CFPVSDDVVIYSVVMMFRRFHHLLADINKKIRTISESGLLTKWQKDSQSEGDNEVEDSSD

AVGHGSVKMVLRVEHVEGAFLLVLLGLATGFLVFLVELSIFYIRKKYKIIMKRRNEKKNI

RK

>IR_CLUMA_CG001651-PA

MTFFTELQSEITDENRFDISICVNEILRKYLKPDYMKNSSTIFLNLLPNFETTSMMTQQR

ILKLLNEDETHQVNLIIKDDAPPQENEPKAREKAKNYLILVTFPTEIVENIKKLQKLSMW

NHEAKFVVVVTERFSDQFEMEVVVSGTFKLFFDYSILNVYVLIQNLDVDNLLQSFIWYPY

DGNSCSNILSYNNLETIDECEMFNHTSEGEKKFEMRELIPEQPSHLPDKFHQCPMIVTTP

IWEPFVIGTSEAPTGGIEFLLIKTIAEKLDMKLIFRVVDDATAFRLVTEDEETGFYSDLI

KRKIDVMIGGLYDNEISRKLLSTTIPYDSDEITWCVQRSGLAPNWMNVFAIFDIMLWIYA

IICIFGCSLFLFISVKIENDRKENFLWALMIIMCYSIGIYGHYDPRRGFIRYYIAFLCFY

GMHFSAAYHSFLLSVLTTPRYSHQTATIHEAIDAEYHFTGGENLKAVFERPDKASNHLKE

AYKPCYEMDKCLMDIERDEKLAVAISRQHATNARIPLNVENMYCFDKANNIFSFSVVMLF

KKDHHLLPAVNVLIRRITESGFILKWKADNEKVKINEIRKQRGDSHENAEPLNLGHVLGS

FGLMFVGFCLAIGAFINEWIVYYLVHKRNNKFLQKYIEEKFLYS

>IR_CLUMA_CG001691-PA

MLTFKIAFLCIISICRSHKLLPDNLCSQHNEMFMEIIFRYFNDQHFLTAVELPDVFDQRM

DVLRCFGANSIRPIDLFNTLNMKFGAKVDTSSVSSFPTTHGFYANVRNETSLFKLFEKVA

SFNPRTKLMVSVEEISSDLVKESLTVGYKKFKLLNIALLTSIARRIDKEDFDHEIVLCQY

NPFTDDRNNLEYPMHHCLDFTPKNMKENLIQMEDFQKLRLGNLRGFPLRISLFEYEMKSV

AVYDEYGRITHYIYPDGELVTVLAKYMNFTPAYLPEIGNGLKYGFQSPSGIFEGALGDSE

YDRADLLANPKLIADYNTTNSVFLRPTAMTKLYFIIKKRITYKKTILSFFSIFDDISMWI

IISLFIIFPISYSLINRIETFLVTGRKKCVFGRNTLYIFQLLNNISSRHSNLNASRITAA

LVFFYALVTTALFQGSLIQKLNLNQNAGSLNKIEELFENNFTIAMAPILSFVFTVQGTDR

VTKRIMEASNGDVTEIVMRSDDAMEELAYNEKLAFLWIEEATGTFLNRFYDNLTGENLFE

RVPESAFEFYIAMMLPKSSPFIDRFNHFINIYVEAGLYQYHVKKAAEDNEKVWIQRIREG

KVPKQNSRTLEMDDLKIAFKLYLFFVVAATIAFFFECLLSFASKRLKCRKFR

>IR_CLUMA_CG001692-PA

MLGKNRKGNRFDVSICVNQILRKYLKPDYMKNSSTIFLNLLPNGETSSMMTQQRILKLLN

EDENHQVNLIIKDDSPPQENEPRALEKAKNYLIIVTFPLDIVENIKKLQKLNSWNHEAKF

VVVVTERYADQFEMELVVSGAFKLFFDYSILNLYVLIQNLDADSLLQTFIWYPYDGDSCS

DVISFYNLEVIDECEMLKDSRDGDPKFELREIVAEKSRLPDKFHQCPLIVTTPIWEPFVI

GSPEAPTDGIEVMLVKTFAEKLEMTLEFNVIDDATAFSLVTEDEETGFYASLINREVDVM

IGGLYDNEVSRKLLSTTIPYDSDEITWCVQRSGLAPNWTNSFAIFDIMLWIYAIICMFVC

AVFLYISVKIEKDRKENFMWAVIITLCYSIGIYGHYEPKRGFIRYYVGFLLLYGLHFSAA

YHSFLLSVLTTPRYSPQTSTIDEAIDAEYHFTGGENLKAVFERTEGASEYLREAYKPCYE

MDKCLMDIERDEKLAVAISRQHATNARIPLNVENMYCFDKANNIFSFSVVMLFKKDHHLL

PAVNVLIRRITESGFILKWKADTEKANLKEMERGDPHENAEPLNLGHFLGSFVLMFVGFC

LALAAFIGEWIVFFLAKKKKEN

>IR_CLUMA_CG002476-PA

MKIVKFYISFILICYHYKNSAANILLQSSSNSSIPNFISWLINENNKREPQRNHNVVLID

LELTYKTEIFNNTLEEILRSNPDNAVMTSKRTKRIPPYRIHTASFIIIISDLDDSIKIAA

CIHNIFLFNKWQDSTKFIFVSTLQESVHRKIFFIAFDSIGVLNKILLRVNQGLLEVFSAK

YFLRKIYSHGVKSQMNLDQLFPDQLKNLHGYTFQILHIQQYPRFMKVAYRDFRGIDVWMM

QIITLKYNSRMNFPISIPWTGYKNISKFIIEMINIRTQKSLDLTLSTIVTLSDLSTKYIN

TYDENGYCALIPLPPRLTFLHFILTPYDTLSWMFMIGLVIICSILWKLSSRNNQRKDSAF

YFFLIQVANFLGQSIPFRNSRRIQLLLLQLCIMMTFIMGNAYQSLIIPSMSRSREGLRIK

TFDELIKSNISIAVTTGFYHILNQSNEFPTLIERSKVIPNHIKFTEFGQNRTALVMRCDL

IESAFFHERHGQVTTNLYYILPDKIMKFYEKFLINSYSPFYEMLQMNYNKLFESGIKQKY

ENKFNINVKKIMEKREQNFIANENYLLRMQDIYGIFFILLFGYGVSFLTFIFELISRHKF

EQIINKLIGSRN

>IR_CLUMA_CG006216-PA

MEVWKKIIFISYILIFAKSEFFYVEELIPRYLSFLVKENNKKELQTQKHDVVLIQMELNY

RTNCYENILEELLKENPENPIFTHSNLNSFEIEGIHTSSFFIIVTDVTNSTILFELFKKI

FNVPKNLIRNYSKFVLVHTRLQSLNLLTMIFIIFDHFGITNGVVIRQNDKLTVFKGMMFL

KTLEQIKTQDLYNFDLVFPDKLKNLHNCQVNLFFIQEETTCVILNGEVDGVHYRFLRMIL

KKYNATMNITKSIDISDGRIITKELQEFFKLYSVSLSLSTLFTPQEYTKEVNTYDEDGFC

ALIPKPQRVTFLHFILSPFDLFSWVGMISSIVLCSILWKFLKKRLAKINSSFYFAFAMVA

NFLGQSIPLRSSRWNQKLLLQVCILMTFIMGNSYQSLIISTMLTSREGLRIKTWEEMFNS

SLTYKVDSIFKRTLAESGEFATVINRMSPFDKYSDYQNYANEEIAIIATCNQIEKNYYKT

EVSNSFYLLPGRFNPFYVRFPLGPWNPLYELLQRNFDYLFESGIRQPMHRFFQDRDKKYI

ESEIRFIENEEYLLKMNDVYGIFYILLVAHLISILTFLCELLWSFKQRRKSRRVKNQAES

ENQ

>IR_CLUMA_CG007411-PA

MLRKFVLCCVFLQFRCSLAAIFILKAETSGNLTIPKYISFFVNENNKQDQTHNYDVVLIR

LELQHQNETFENIREELLKVNRDNPILTFDLFDESQPKVQFPQIYAASFIIIVTDLTDSA

PLRSKLFDILEKITWQVSIKFVIITPKMSPKLLNEFVGALNYFGFINHLILGISETVNAY

SSNMFTSAIYWIADEDIKNFSVVFPDKLKNFQSYQFPMLFVKEYSRCMIRGRKIDGINFR

VMKMILKKYNGTAKITTILDPADPQASLKVQKYFYGTNNAAFSLSTVMGLQQWRKWIYTY

DENGYCALIPKPPRLTFLHFILTPFDGLSWTFMIISIVVCSIFWKLLTRSYNSLRSNSPF

FFIFGVIANFLGQSIPFRNSRRIQKLLLQLCVLMTFIMGNAYQSLIIASMSTSRDGRRFN

TWQEMFNSNLSFESGEIFYNIMNRSGDFPDVVRRINVSRKINFERISLKNVVGVSRCYDL

QAVVNYKKDVDVMEHFYLLPEKFMPFIEKFPLSPSSPVVETFQRNFDYIFESGIRQYLNR

QFQNSFKRNVDDITKFIINEDYLLTMNDVYGIFLILVGGYVVSFFVFICELIWRHRKKIT

NLKIKTKNRKLKIERKHQ

>IR_CLUMA_CG007415-PA

MKMLIKFVLCCVFLQFRCSFAAISILKAETSGNLTIPKYISFLVNENNKQDQTHNYDVVL

IRLELQHQNDTFENIREELLKVNRDNPILTFDLFDESQPKVQFPQIYAASFIIIVTDLTD

SALFYQKLSVEFYNKTFSDSTKFGLITSKKSSKSMKQFFTILDYLGFINLIILRDDDDHQ

IKIYTFNKFGKIIYAISSEDRWNFPVVFPDRLKNLNGYQFPMLFIEEYSRCTIVNDEIDG

INFRVLKMFMNKYNGAMSLTASVNPADPQISIKIQNYFSKNLAGISLSTSVKLSDWKKWI

YTYDENGYCALIPRPPRLTFLHFILTPFDGLSWTFMIISIVVCSIFWKLLTRSYNYLKSN

SPFFFIFGVIANFLGQSIPFRNSRRIQKLLLQLCVLMTFIMGNAYQSLIIASMSTSRDGR

KFNTWQEMFSSDLTYEVGEIFYVSLKESGDFPDVINRMNVAREIQFEKLSLQNVAIILRC

NDIQTEIDYNKDVDVMKHFYLLPGKLTPFIEKFALAPSNPLLGILQTNFNYIFESGIRQY

LERYFQGLRKKNVDDITKFIINEDYLLTMNDVYGIFLILVGGYVVSFFVFICELIWRHLK

KIANSKIRANQERKI

>IR_CLUMA_CG007416-PA

MLRKFVLCCVFLQFRCSFAAISILKAETSGNLTIPKYISFLINENNKQDQTHNYDVVLIR

LELQHQNDTFENIREELLKVNRDNPILTFDLFDESQPKVQFPQIYAASFIIIVTDLTDSI

TWQVSIKFVIITPKLNLTLLTDIMWCLNHFGLINHLILLGSSQIVNAYTSNMFTDKMYSI

TLEDIKKFSVVFPDKLKNFHNYQFPILFLNEYSRCMIRDGKVDGINFRVMNMIMNKFNGA

LNITAIFDPVDPQASLKVRKYFYGSNAEFSLSTTIELTHWKKWIYTHDENGYCALIPKPP

RLTFLHFILTPFDGLSWTFMITSIVVCSIFWKLLTRSYNYLRSNSPFFFIFGVIANFLGQ

SIPFRNSRRIQKLLLQLCVLMTFIMGNAYQSLIIASMSTSRDGRRFNTWQEMFSSNLSFE

SGEIFYNIMNRSGDFPDVVSRINVTRKINFERIALKNVVGVSRCYDLQALIDHGTDFDVM

EHFYLLPEKFMPFIEKFPLSPSSAVVETFQRIFDYIFESGIRQYLDRQFLNSFKRNVDER

TKFIINEDYLLTMNDVYGIFLILVGGYVVSFFVFICELIWRHRKKITNLKFKTKNRKRKL

KIERKHQ

>IR_CLUMA_CG007417-PA

MKMLRKFVLCCVFLQFRCSFAAISILKAETSGNLTIPKYISFLVNENNKQDQTHNYDVVL

IRLELQHQNDTFENIREELLKVNRDNPILTFDLFDESQPKVQFPQIYAASFIIIVTDLTD

STLLRSTLFDILEKITWQVSIKFVIITPKISPKLLNEFVGALNYFGFINHLILGISETVN

AYSSNMLTKRNYWIADEDIETFSMVFPDKLKNFQSYQFPVLFLKEYSRCMIRGRKVDGIN

FRVMNMILKKYNGTVKFTTILDPADPQAALKVQKYFYGTNNAAFSLSTIMDLKEWKKWIY

THDENGYCALIPKPQRLTFLHFILTPFDGLSWTFMITSIVVCSIFWKLLTRSYNYLRSNS

PFFFIFGVIANFLGQSIPFRNSRRIQKLLLQLCVLMTFIMGNAYQSLIIASMSTSRDGRK

FNTWQEMFSSNLSYESGEIFYNIIKDSGDYPDVVSRINVSRKINFERIALKNFVGVGRCY

DIQSVIDYGTLFDVTKHFYLLPEKFMPFIEKFPLX

>IR_CLUMA_CG016004-PA

MNVLRKFNLFLVFLSSVKLFTSAGFFDVSENQIIPRYISFLINENNKKDQTQNHNVAIIR

IEYNPVTDIFGRVAAEILKENVFNSVVIYDYMITKQRIYKKIISRASFIIIVSDLWNRSF

LIQTLSNIFINNPWKDSTKFIFVYSHDNRLTPEAMYEIFFVFEWLGVLNLIITSEQLIYG

KKIRVYGSNEFQNRVVFMIRNPSNIDSVFRDKLKNLRDYEFKIFLTDDDVRIAVNDKTRP

IEGIDVSIMKIIAEKHNGRIMFETLLYSSDANFPNKFVDLLTGRKSDMTLSTNYYMQKVL

KYINTFDENGYCALVPIPPRLTFIDYILTHFDNYSWGFMCLSIIVCSVFWRFLTRQTFPS

NSLFYFIFGVVANFVGQSIPFRKSRRIQVILLQLCILMTFILGNAYQSLIIGSMLTSRDG

VRMKTFDELFNSNMSFKVDGMFYNKMSFSEDYPSAVSRMQVVNSAPKDIEKSARENIVLI

GGCELINQLMFFHANASVILHYYYLLPEKIMKFYERFPINNQSPFFDFIQSNYLYIFESG

IQQPLKYFFSLIAGNKGEKIVEQQNQFISNESYLLKWNDIYAIFYILIVGNIISFVTFIF

EMIFRYYKKCFKCLKCSKCFKCFKCIR

>IR_CLUMA_CG018833-PA

MRLEIVLFFVVYTSAIKLDGLLEISSLEEEIFKNVSSKLADIMKNDEAYKKGFIELTVSE

TNETIESGKGLQELIRQLNLQDILIVFRSRKSSHNEQEYFRLPDFGLILLDSKCESTTNH

LLTMPINFPDKFVNLFVICNSLVSKTSARRNMNFIKDDLMDLKYRKFISILIFNSSEYEI

SSGLTEDKIIKVVQYDMQPFTKIVNRKITGPEGKFLDTFCTKYNLTYELINSNNITNQVN

FAEIQNSIFNEIADLNLNNGFSISHPRFVKINIGILDGSCMLAPKNIYISSYENSSFPFD

TMTIVLMIVSIISTILLWKMLSSYAKTQFKISFIMINIFKCTIGQEVSGEKIMSRKEKIV

IYSYIFASMIFVSLYQSILIALMLSEPSLRSVESLKELNDSNTNIYEYMHFDSDYTPSFR

EEKILTKLGVRGKYALELSVPMNFDQNLAYFVTCSYADEFLKSERNYDGNRRLLDKIPEQ

LSFFPQQYILSSAFLLRKEIEFVASVLRESGIRNYWISKAVDDPLDQSKKKVVHVEEKSY

VDFKNMIMPFVVLSSGAVLALFAFIFEIIISFMNERKMRKVVKVKQNPRRHSPKQFLK

>IR_CLUMA_CG020671-PA

MKTIQVLVHLFVLVKLNKIFDETIKELICANETKISSQAILHYFEKQRSIFVIDLSKDSF

FNTNICNQFDSISIYVFNKWNKVNIEDKVVDSGLSAHSRSRGFFLTCTYREALQLFKQIS

DYNPRVKTLISLRSSNFSEARKLLQIGYEKFKMLDVAILMKIDQFSEVKFSICLYNPFQG

DKIMRSPEFRCFNISIESKPFVAIDTFIETRVRNLHQFPLRVNIFEEPLLSNAVKNKQGK

IIEYRFLVGETIKILKFVMNFTPIYITSVDGSRQGFNLPNGSFTGSLGLIEDNSVELAVN

PIFIADYGTKKMIFLQPIAIKSLYFIIKKRKTYKLHMTSIFFQYDNWAYATATALMFLLP

LIYVVVNKMESKMMASQSEKTIPRNIFYVYALIFNVSMQHSTHISSRMVVAVVLFYNLMI

SSLFEGTITKNLSRSQQNNEIKTFDQLINENYNMTMRKSLAMIFIKQGGSKYSDKLREVA

YNRKELVETYGEGLRKVLNVPRVAYLTTRATSIITDRYYDSKTGVNKFESVTEPIYEDYA

SQLAPKHSPFIECFNFWINVIREIGITKYQFSRADDSNKILMIQRVKAGKVSRDDDKVIQ

LNDLASTFYLYLFQMFICCLCFLFELLYFHLELRKRTLGQSNREVSDDKFFEFII

>IR_CLUMA_CG020672-PA

MFKLFIIFLTLYSVASGEIEVEKDPPKLVLIESILRKYLVDQESLTIFDTGKSKNVFKYK

SQFLSIFHSILPCITYRNLNDSLVRKISDMGNEISLPSTSKSYLALGSYSISSHLPLYAK

INTNGKWIFLLIELKHVQVERILINAWTNHKMANILVFSLDKRLKLFVTSFNPFKKQGNN

YGTFWSMQINKEKFSTILKAVENIFENKISNLQEYPLKGIFFRKEDYLDEIMVDIFQKAF

NVKYEIVLPRHGNFIG

>IR_CLUMA_CG020675-PA

LLIELKHVQVERILINAWNNHKMANILVFSLDKRLKLFVTSFNPFKKQGNNYGTFWSMQI

NKEKFSTILKAVENIFENKISNLQEYPLKGIFFRKEDYLDEIMVDIFQKAFNVKYEIVLP

RHGNFIGTRLANGSFNGLLEDIEDGVADIGLNNRLLVPMNSTNCAFLNPVVVTEMKYITS

KKFPPPTQIFHFVLQAYDIETRWIYFCCTILTLLSWYFLDFIHHQLKAVEKREEIGKLLL

IITAIQVSSTAYIRDNIPSYQRILVGSLLIVSLIMCNAFQGEILRNLRSPQKSPDINSLE

DLLQSDYELTAIIAVPDLFQPDENDSNVNHIQKRLYYRQTVSTNFSTPVPKTIAENPKQA

FLMRGEFVKKFLKIEINNATGEDFCHIVKESPISFFLTYIVPKTSLFIRRLNRAIAEAKE

FGFIEKAKNKADAILKQRQAKRLNILLKAVQNHPPISIENLKNVLIFYGSFIVISCGILF

MEIFLNCFKNRAAMKNKKKRIRRRVKLKY

>IR_CLUMA_CG022105-PA

LNDSLVRKISDMGNEISLPSTSKSYLALGSYSISSHLPLYAKINTNGKWIFLLIELKHIQ

VERILINAWNNHKMANILVLSLDERLRLFVTSYNPFKKQGNNYGTFWSMLINHENLSTIL

KKVENIFENKISYLQEYPLKGVRSGDLTEFHKNLNELMKEIFQKALNMKFKTVAPRDGKR

IGTRLPNGSFTGVLKDIEDGAVDIGLNNKLLVPMNFTNCTFLNPVVTSDFRYLTSKKSPA

TTYLFYNVLQAYDLETRWMYLVCLIITLFCWYFLDFIHYQSINVSKREEIGSLLMIFIAI

QTSSTLSVKDNGPSHQRILVGSLLIVSLIMCNAFQGEILRNLRSPQKSPDINSLEDLLQS

DYKLTAFIAVPDLFQPDENDSNVNQIQKRLYYRQNISLNISNLGCKPLMRNPKQAFLMRG

ELAKSFLNVEINNATGEDFCHIVKESPISFFLTYIVPKTSPFIRRLNRAIAEAKEFGFIE

IAKKKTDAILKQRQAKRLKVLLKAVQNHPPISIENLKNVLIFY

>IR_CLUMA_CG022106-PA

XEVEKDPPKLVLIESILRKYLVDQESLTIFDTGKSKNVFKYKSQFLSIFHSILPCITYRN

LNDSLVRKISDMGNEISLPSTSKSYLALGSYSISSHLPLYAKINTNGKWIFLLIELKHIQ

VERILINAWTNHKMANILVFSLDKRLKLFVTSFNPFKKQGNDYGTFWSMQINNENLSMIL

KVVENIFENKISNLQDYPLKGVFFREDDCLDNIIVGIFQKALNVKYEIVLPRDGDTIGTR

LPNGSFTGELKDIEDGAADIGLDNRLLVPMNSSNCAFLNPVVVTEMKYITSKKFPPPTQI

FHFVLQAYDLETRWIYFCCTILTLLSWYFLDFIHHQLKAVEKREEIGKLLLIITAIQVSS

TAYIRDNIPSYQRILIGSLLIVSLIMCNAFEGEILRNLRSPQKSPDINSLEDLLQSDYKL

TAFIAVPDLFQPDENDSNVNQIQKRLYYRQTVLMSFATAVCKTIVENPKQAFLMREEFVK

ELLNVEINNATGEDFCHIVKESPISFFLTYIVPKTSPFIRRLNRAIAEAKEFGFIEIAKK

KTDAILKQRQAKRLKVLLKAVQNHPPISIENLKNVLIFYGSFIVISCGILFMEIFLNCFK

NRAAMKNKKKRIRRRV

>IR100a_CLUMA_CG017665-PA

MKFLISFCLFFGSCFIKVSTSKFANEFINASNNTIVVINIDNSLNESYNFITNSDKAFLK

FNFQSESFSYLEKLKRVVRRKHVALTTQLRRYREFIMLKDFIKILDCLNCDYIIFSSTLF

LNSVLKLFVNSFGNFLVILIDNKTSFSQQELMDVLNRTWIDNIALKVSLAINDNVYSFDP

FHRNPNGIFGKLNQFKQTTCSQRIKNLNGYNLNVEMFSSIYSALSVKNPKIVEDFYGPDV

NVATFIKEQLNATMTLVVNDGEKFGFRKPNGSLTGALKSIQSRKADVTFVGYFIKDYETQ

DVEFSSSIYSDKLCIVVERAGRIPQFILPLIIFDNSLWIFLGIETVLGILFWSFLRFLNN

KLYIECYEKQIKFNLPSHTKTSPTFLQYIQIIVDFTMLLFGTPMRRFPRVKSERVFISSV

FILSLNIVALFQSSLAMVFIKPMFYENIDTLEKLSEGNQNIIIKYPAMLNDLFPEDSSDT

FRDLHNKMKLITKSSVGPREIIENLHMATVTRKQNFNMHSIYNDYHMVAECPKHYNLAYI

FAKHSIYSEVINALILDIVRFGLMNKWINDVEYESKLKNNLGIQDVVSKSLTLNDLQLPF

FTVIFGQALAVVVYIIEFFVKFKTKAEHGIKTAN

>IR21a_CLUMA_CG011952-PA

MLLLVVLPLIFIENVDLKFMRSDDILRNLITYDSVQVKRNHEKLLGNAFETQYYSPFKAI

ANSSNDTESHQMKLRKKPSIRFKRKTDSTFRGRPKTIQEVWSRNFNISTQQFSQSTSLVT

LMNKIILKYMSACIPVILYDEYVEKSEGFILLRLFQNFPTSFIHGKIGKNFTIENDILLD

PPHIRCRSYILFVSDALKSRQVVGPQIESKVIIVPRSTQWKLQEFLSSPLSRDIINLLII

GESYSTDLTKERPYVLYTHQLYVDGLGSNKPKVLTSWLKGKLSRPHIDLFPVKLRKGFAG

HRYSVAAVNFPPFVFKKLSTDGVGNLHIEWDGYEYRMLEVLEKKLNFTFEVSEPRELEML

GPGDAVGLSISKRQHEIGMSGMYVTTERNSGTEMSVSHSTDCAAFITLTSKALPRYRAIL

GPFQWQVWVCLTFTYLIAIIPLAYSDSLSIKYLIEKPGQIENMFWYVFGTFTNSLTFSGE

LSWSNSKKTSTRLLIGFYWVFTIIITACYTGSIIAFVTLPVFPETVDTIRQLRNGFYRVG

TLDRGGWERWFVNSSHPDTVKVLQNLELVKNLQEGLGNVTKPYFLFPYAFIGSKAQLNFI

VQTNYSDDKLGRHSTLHISDQCFALFGVSFVFQRESVLRDKINHGILILQQSGIINKIKN

DVRWDMVRSSTGKLLQISEGKTLRIANQEERGLTLADTEGMFLLLGIGFLIAGAALISEW

VGGCTNKCMQFMRVKREQEEETHRIEEEKENARVEAETARLALKSASSVIGITFSVKTED

EKTVQDGNEPQELKPTSEVLSGSSRNSRHSRSDSITYPDLNAAMLSEMYHGPKTRASNII

MMDGKMMSESDANEHANQMKKDDEMKQRLSDGGSGEVLRDFDFLNNGKDEDADEDKDNNV

SHHVCEVEINLQAPTDTEFDN

>IR25a_CLUMA_CG000026-PA

MLSTHNVFINEVGNEIAQKAVDVAGNYIKKTPSLGLSVEIMSVEGNRTDSKGLLEAICTR

YGESLQASQPPHVILDTTKTGVSSETVKSVSSALGIPTVSASFGQEGDLRQWRNINDKKK

NYLLQVMPPSDVLTEIVRSIVRYMNITNAAILYDETFVVDHKYKALLQNIPTRHVITAIA

VDTERSEQITKLRNLDINNYFILGTLESIKKVLESAKKEYFERNFAWHAISESKGDLVAN

VENATVMFIRPTPDGQSKDRLGIMKTTYNLKGEPEIESAFYFDLALRTFLAIKALLQNGL

WPPNMKYLTCDEYDGSNSPDHSIDLRSYFFDVNEQPTYAPFYFPKPSETFNGFSFMKFDA

DINAVTIRGGASVNTKNLGIWTAGLDSPLKIKAEDEMKNLSANTVYRIYTVVQAPFIMRD

ENSPKGFKGYCIDLINEIAKIVEFDYTITEVEDGKFGNMNSKKEWNGVVRKLIDKQADIG

LGSMSVMAERETVIDFTVPYYDLVGITIMMVLPSQPSSLFKFLTVLETNVWLCILAAYFF

TSFLMWIFDRYSPYSYQNNREKYKDDDEKREFTLKECLWFCMTSLTPQGGGEAPKNLSGR

LVAATWWLFGFIIIASYTANLAAFLTVSRLDTPVESLDDLSKQYKILYAPLNGSSAMTYF

QRMSDIETRFYEIWKNMSLNDELDAYERSKLAVWDYPVSDKYTKMWQAMQETGLPNSLDE

AVRKVRNSTAASGFAFLGDATDIRYQVLTSCDLQMVGEEFSRKPYAVAVQQGSPLKDQFN

NAILKLLNKRQLEKLKEKWWRKDDVSAKCEKPEDQSDGISIQNIGGVFIVIFVGIGMACV

TLAFEFWYYKFRKVPKVFGSDDAFNNKFTKKNASIKGNNSFGSGGGLIDTGSNNTLKSRY

PPAGNFRARF

>IR40a_CLUMA_CG009212-PA

MDARNSILVNELVMQLSKRGLQTCIFNEKEKFFKFIETNLLGSLEVTGLVFHDPKVLIPE

IHARNLAHRLSLYVFFWNVKKLPKNHEHLNLQEPLRTVMITNPRKFVYRIYYNQASSAND

GNMKLVNWFDGNNLGLNAEPILPDMKNIYKNFNKRVFIVPIIHRPPWTFISYKNLNESGD

DLIDQFQMPQNDSDISLDISGRDHSLLEILSEKMNFKFKYIDVKSIISSDNITQPHELGL

KMLQNREADLLFGDTVITSEKLQEIEFSFLTLPDSGAFLTHAPRRISEAFALVYPFDASV

WPPLVFTVVIVGPILYFMVVILEKLKKRKKKQKYSNSYGKMIYTREIFSMKIDGKRKKHA

RILEQDGLLSLCVWFTCHIFLRQPANFPYDNNSVRLFSIILWLSSTYVLSDLYSAQLTSQ

LARPSKELPINTLQRLEHVLNKSESYKLLVETNSASHNILKNGIGIMNRLYNRMVHQGSN

STYLLNSVEEGVQVLLNKDSTVVFAGRQTLLFNMKRFGIKNFQLSEKLFTRYSAISLQKG

CLFLDSLNENSLMKLFEGGILDKITNDEYKKMYQSMPMDLSGGEKKNADQENGKIEGKMN

GEDSMGSKAKTADEKEQTALNLRMLQGAFYLVMIGHIFALITFAIEIQYRKP

>IR41a_CLUMA_CG007498-PA

MNSTVEEVLRFEDAVQNILNFVVSQYFSLFYLVCVITSKTSDIKPRLSGFNLYSFEYENS

SKESRDNFRKAVESGCQSFVISDDAILSFMWDFRDIHDDCIQRFPNKHIIVYPTQAKGKS

QNLISSWKTISSIEDLPNILLVEYQELTQTFSFLTTKYAGNNNDSTDLVMLSEIYISDNP

RMKLQSVKLFPDKTSNLQGREIVLAIFNYMPYVLWKEVNKTNANFNSREKLSRTPLYIDG

TESWVFLEFYEEGEWGVIYENKTGNGILGAVAEKRAEVGVGALYSWYHESLYLSLSKYIS

RTGITCITPKPGLLSPISTHILPFSTELWIAVIVSYFICALLYKTLIFFRNDNDQSLNIQ

SDESLSKIFLDMLGIFVLHSVALKFKQFAVMIFIAAVLIVGLLIGNSYSSAMASVLTIPH

YEKGIDTVEELADSDTEWGSTHDAWIFSLQLATQPMILKLLDKFRTFSKEILQYRGVRQD

ISFSIERLPYGHYAVGEYVTEETVKNYQIMLNDIYYELCVAMSTKTWPLMEQLDDLILQI

AQSGIQQFVELDVVTRNTNNKIQAEIALSRHRDNVGPINLSPSHVIGPFILLGVGLTFSS

IVFISEILIKRNARMKCLK

>IR60a_CLUMA_CG003005-PA

MFLISCVFMLNLLFNRINCETYLRDIEKGEAIGGINCIKQVEREYFWTQFSHQVENLAVF

HTTNMTNLATEIEIAYLRDLHDRIINRDEKGKHFQLKVLSHFAKGITRKDIVLTNVYIII

ADNAETLNQSLDALTTSPSFNEIAKFILFINNPSQRNGGEEIASYFLIKMFKEHRALDVV

LLYASDAFAYDIYTGNPYHENKTDCGEMKILNIGKCANGKFEDENSTKEVLQTPKIPSTI

NDRVYKFCARVQEPFVNEGCHDGLEIDILQLVMKEMGYKVDIFCSKLGRGERMDDGTWSN

LLGQVRNGECDIIAGGFFPDHEVHAEFEATEFYLQDYYTFYVKKASFAPRWKSLIIIFKF

RTWVAFSIVVLVSWISWYLLGRMSSESKAHKQIVLTYLNVLALSLGVSTNNRPNSNPLRL

LFIILSLYALTLNAIYTSKLITVFTSPPYDYQIETIEELLESEIPIGGRLENQDWFENDD

EFDRMISHRYNYSETFRPSTKSLKRVMKGEQALLISQLYVRSNKYRNSVFGLSKSIFSNQ

LEMIFERGFPLQHRINKIIASLRDMGYMSKLFKDFYYNMTIIESIKKLKRFRKKFGSDIE

QLELALADDDDEDEEETPRIALTIEHLDGAFSNLFLGLAISSGIFIMEIILKSKFINRNV

KLFGRLLSKMFCNHKRKRKQEIIEGKMIPFRN

>IR68a_CLUMA_CG015147-PA

MLVFKSLFVLLTISTIFAFNFTNFLNDSFYVLKDEIETREIQDDIIDLISSILIKFKVSK

VSIITDSIYKGHLINTNLIDSLEYKIIFMMSIKDSEAFDEKPSGNIETMLRIMKREIIDA

YVILITNGIQMSSFLKFADYYRLLNSQALIILLHDYRLLSPNFHYLWKRIVNVILIRKCE

TIHKDMYEILTVPFPEKIREILVLSTINYWSPLRGFKWKHKIFDTRAKNNQLNGVELNIV

VLEHTPTVFKQLEENETIGYYGLEIDLITTLSSVMNFSTNFYESEDAITEKWGKKVSQAI

FSGMLNEMDQSRADIAIADLHYTSFNLDIMDLSLPYNVECLTFITPETLGDNSWKTLILP

FSFEIWIGVLTSGKPTEKLQYFIKDLFDEFSACILYTYSMILVVSLPRLPFRWSIRVLTG

WWLIYCLLVVVAYRAALTSILANPQPRLTIDTIEMLANSLLKCGAWGEQNKNIFLSSSDP

ASQKIGTKVEHIDNADKAIEQVAQGEFAYFENRFTLQQLRMQHEQQKDSIQNLHIMDECV

INMPISIGIEKNSPLKEQIDKFIRYIIEAGLIKKWLMDSVKGFESNTESQPEEALMDLKK

FSGALVALVCGYVFAILVFTIEKCYWKFIIEKHPYYDKYFRAIKLPKIKNTEPQKITKSK

RPMTSDKKNHAVLN

>IR7.x1_CLUMA_CG000997-PA

MKLKLAIILIFLIEIQAILNFHLNHKTDSLGESLRNLIKSFYEKKCFDVAFFIENNQGFG

VHLMPNVLLNFTRPTVSISLASEALNVKVYCGIFFLEKENSINKIILSDKVHRSGYYVII

LEIKNRKSLKNIFKVFWKNFFFNINVLGKEKVLQNISLFTFLPFNGAICGDVSPVIINEF

NENYNDWSTNEYFPKKLKNLNQCPIRIGSHSNPPAVIDRNESSVNTYGGNMIDFVRMLSS

VLNFKIDFHLYSVGAGAFANGTATGLMEKAYSNDVDLIIGLLSLQQIRMEYLSETRAFYH

DDIILVVPPPPLIPSIRKLFMPLDFYCWVALLLILLISSIILTILKILPKSYHNLVIGEN

IKNQYLNILEVLFGESQKKLPVKNFPRFILMSFMVFCLIMRTCYTGSLFNILKNDISSKK

IKSIEELNQMDYKFIIYDTLAARLNDKKFMKREVISYSKVEEYCRMTLDPSFKGAVFGYH

STISYQNLLNRKEYSVNILQEPLVSNQIVSYFTKNFYMVNEFNEKISQFKASGLINFWIS

KYFNEDGEIDQDMASSITLKHFSGIFTIYIYGLICSLIAFIIELISKRTKQRRQVTVRRS

RFLY

>IR7.x10_CLUMA_CG003835-PA

MPFTKNNCRDTKPILINQYQNGKFIKPSNFFDPLKIKDMKGCPVRVALTNSSDAVFYTSI

ETSKNKMCGPEIKLISTLSKALNFKLNFTFIGHIGFLFENGTSEGPFRVLLDENADLAVG

NWWLKTDRLKFFSSTHSYAQQPLIFIIPPGKPVTHLERLISAFSLQSWICIGLFYLIGSL

VILFVQLKSPCVQEFVFGAGVTSPMFRMFSVFIGDTLNNLPKRNFARFLLMSFIIFTLII

RTVYQGSYFRQMRTFRHHKEVASINEMIEKDFNFYANPGMIDFLLGFPAIRERVRELNNE

DVPEFLKQITTNPNFKAAYANALLIKDYLNSRNSDESQHICKEVFATNLPVVIYSLTDFY

LLNALNEKINAIIKAGLIDHWTRESCRQNVRKVQESIKPKVLTFDDLEGSFLILIFGCLL

GFIAFLCEKFLL

>IR7.x11_CLUMA_CG003836-PA

MRLLYKLLLLMLINLLSSQAENSFSSLGSFKTLSNAVVKVIEDVWGDDQKSVNFIISINN

PENIYVSSIVSDIMLQSSLNPTVGYHVETIEYLKIKQKLPKLSAIVVIETFEDFTNFNKI

LTSDLISFSSYQGDILLLFLNGELTELNEIFNILWKKQVYNVNAIYEGTNEIVEVKTFFP

FKVNNCSDTTPTLINVFENDEFKNDLENFFPDKMKNLKKCTIRVGTSNSSIPYVFASKLP

NGTYELSGRDISLIRTLSELLNFEIEFAYVGEEGHLIENGSSSGALKMLLEGKVDLIISD

FWLKANRMKFIDNCIPYISQHIAFVIPPGAEFTAFEKYFKPLDSYTWIGLSVVFMSAFLV

IYIFEKLPKVHRNFLIGENIKEPYLNVLVAVYGGSQHKLPDRNFARSLLMVFLMFCLVMR

TIYTGSLYRYLQAPITHSEAQSIDDMLERDFKFYTVSSILDLLQGQDRINKRLVLFPPDR

RNEIINEINTNPNFKGALFRSLTGILYYNQLNYNKTQSMICKEMFMMFHVVIYVPKDFYL

KEAINQKIQILIASGLIEFWHKSIIDQRFRRIEEDDEPESIKLEYLLGCFNMWMILCIFS

LIAFIVELIKGKILKKV

>IR7.x12_CLUMA_CG001079-PA

MFSMWKIFCFIFVVKPVLGTFEHFELLNYSSPLSRPLANLITNAEYNVSILYDRRESPKE

VEDELDRIFEIIDGDMDVIIKDFSRPILQKHEGNSFNVVFIHSNESLELFFSIVNSDKFT

SQSFFFIVIPASFKIFINLETLFERFLNYGVPYVYTLTVAPNDEDAILHTYYPFSANKCS

DSTPVIANYYTAKYENWTEADFFQFKVQKMFGCPLRVATFHFNPFMILKEDEDGKISVDG

IEGSLLNVLSERMNFTIEIITSNDRWGIIFENGTTTGNVQMILNRTADISLGYFSVHDKR

QKLMSSTSVYLISNLIWAVPPGEPYSPLEILLKPFEKSLWNWFFIVLISAFVIIRVLRMF

PKKVQYFVFGRNVRHPVLNVVNIMLGGSLPKLPVRNFARTIMMLFMFYCFIMNNAYKGGL

FQFMKSDQTKPPLSTTTQLYNKNFRFYSIPDGKIFLNETPKILDKTIFLNNPEEYDEGLK

HLRDPKFKRALMITQEKLAFENIKNSPDVYYHRANDVIFRQHLVIYMNKFTIFEWSFEDI

ILQLVSGGFMDAWIKKFTDTDILKDRVEERELALTLDQLMGAFQLLGVGLFISFTVLVFE

NCLFNIKKCLKSNSDQFCENCEMK

>IR7.x13_CLUMA_CG001444-PA

MKSFQISSFMVLLNFYFCLTQKSFQILNYDNKLNEAINELNLDFMSKLSSNIRIITGSQQ

KFLSEMKFTWNNLMSFELMEISTINSFKLLPSRANIFLINSSHDFALINMRNFERGGYFL

IIVEDCSKIDSDEIFQAAWKQYIYNVNILCENQGMLIVKTFIPFQPTSCSNTSSVTIQNY

SRKTIRNFFPEKVKNLFGCPIKLATFEYPPITMREKFDNGTFRYYGSEMDLAFGLADALN

FSYEITFIMRSGASGLLLENGTATGLLKDTIEGDVEYLTGFYYLTYVRTKYMSFTQSHYS

IPLIIMIPPGEQFSPFEKLFQPFEKIVWYCLLASFGISIIVIMIINRQGEKIKNFIFGEK

IKTPYLNLIIHFVGSSHHVLPSTNFARSLLMMFMLFCLIQRSIYQGSLYLFLQSDGRKPE

VTSVDEMIEKDFVFYIRETLEHNIRHMNFYNRRKVVQFNDYPELRLKTLDSSFKGGIIQP

LLEVIYLNEQNYKNFTFNVLKEYLFDVQIVNYYPKDFYLAKALNDKIGTLKAAGLVTLWM

DRYIDKSYIKINKQKTSARRLNIKQLFGGFQVLLIGVSLGFACFLLEISSPLKHFQFLRK

IFL

>IR7.x14_CLUMA_CG001445-PA

MKVVLFIGIFTLIKAWKVGENEIEKYDKTLMKATKSYLKSYYEQNELDIAAEFSDENLCQ

MLHMSNAGYKVKLQCFAHASVGKMSFESGVIIAQRLSSALEFLRKLFAAQKVKVKNKYLV

VLLNETDNNAEEIFKLFWSRKFMNVKVLTVVDDMTILLTFNPWKSRFYCNNTTPIIIATF

KNESWHDANVPSLNFHQCPIKVATSSYAPAVIFDESKEGLERFSGVDVRLVKELSKILNF

TLEIDLRTNFFGYIQDNGSASGAIAEVVNGNSDLVIGFYILNELKTRFLSFTQPHLYLPV

GVIIPPGELFTSLEKFQQPFTTIVWFSITGVLGLGFIIIFILHFPVQKSKLSEAFIVIAG

TMLGLSHKTLPQRNSTRIALMAFILFFMIIRTLYIGAWFKFLQVKQRHPEISTIDELIES

DIEVLMYPTFEQMIRGWKLHKKANSLSFSSFYQKLMMMKEPLSNLAVVATIDEVYYHNKF

STDTPPHIFLKEYLFTAPIAMYFTKESYIVEIFDDKINLIKTAGLLDFWSSYLMDSDYLY

VDLDKDHEPEQMNFQQLSGAFEIWAFCCSFSFLVFLCEHFHYRLANYLCHCQR

>IR7.x15_CLUMA_CG020518-PA

MYAQWTKLSVLHRIQNVPHKMIQNLLKFLIFSLSVKGILSFTFEHEIELDELHNALLKVV

EDSFVAVTLDIITCTHNNDIRSDIIGSLLEESQGSISYRIERCEELNDSIIRKYVLILMD

HSKTDFMIKQLTPHRFDFSGFYLIHYVNFKATFDILNNLFRNLSDLFIHNVNVISNDSNV

IQLKTFFYFTEKICNSTEPVVINRYENNQWKSKEFFLSKTKNFFGCPLKVATFIYEPAII

MEGSYDDNNYTLGGSEVEILKGVANVLNFSIQYNFDPTPGAWGRIKSNGEASGSYLKVIE

KEVDLMIGSITKTYIRTFYVAFSTVVNFNDVLVVVPPGQPFTAFEKMIKPFEGIVWIVLL

VILTVGFTIAVVLSRKPHSALNRIVVGKEIKMPAMSIIVALVGGSLHVLPKRSTPRVLLT

SFLLFCLVIRTLYTAALFNFLQSDNRRPGLSSLKEVIDKKFPVYVYPSFYDNYKELRLLR

RAKTFNSSIGEYYEKVHDANFEGVIPYFIDNIAYYNKNNYKKFVLRVLKERIFSVQSAWM

FPKNSFLVEIFNEKLEAFKENGLLDFSIKKYVNPKYLNIKKTKSGPKKLNFENLWSEFEF

LFIGIFFAFLIFLFEFLHFRLNLRKTGKVRVSEDYLKI

>IR7.x16_CLUMA_CG004578-PA

MTSNKSQWKIFLAIFINYFILLTHQFSNYLTPLKESENLESSTCSDPVYQIFKFINVNEI

NFILNRENFDILDLSEKYYKSISRDFKVNVYDFSDLKEIQKVFTVFLLIENEISLDQFIK

EVSSEHFYFQGYFLIYINDQNGLNMTRIFHEFWKRHIYNVNEISFKNNSIEMRSFIPYQK

NKCDDVNPKVINKYDINLCLWTSPDIFPNKLRNFYKCPIVISTYIYPPTIILDNINGTEI

ITGYDIELLETLGKMMNFTLKIELVTGQTAWGFITANGSSGGVIKKVMDGEADVAIGGYY

LTLTRAKFMSFCIYGNTKIIFVVPPGIPLSAFEKLLKPFSFYSWLALCITLFIGLAVIYV

TKLQKPHIQQLIIGSSKSSPYMNFCNILLNGLQQFSPKSSFSRSLLGIFLLFCMLIKTMY

QGALFKFLQTDQRHPQVQTIDELIEHKFDFYMYQSFEELSKGLKIHHRRKLIVNKTIEYY

QLKTLDPYFNGVTVGPLTEVWYLNQMNSKNFTLRVFPEILFSIPIAMYFPKDHFLKKIFD

LRVSELETAGLIDKMILKYLKPSQKIIPTHSPKKLSIENLIGGFYILIIGCVLGFVAFLL

EIIIQTKKFCLMSEDNQQTH

>IR7.x17_CLUMA_CG004579-PA

MFEILIKLILIIISSNSIACHASKYKQTHENSHDEISQVIDQFFSTRSAYFNVIKCIKND

DVAGMIIGTLLFKTHGSISYRIEDCKNLKDSTKRRFVLIIMDHLNTDLMIEELTPNRFDY

SGFYLIYYIDNQSDIDILHYLFKKLSDLFIYNVNVICNDSSGIQLRTFFFFNERSCNSTD

PLVINKFENNRWISKDFFPRKMRNLFGCPLKVATFIYEPAVIMQGSYDDNNYTLVGSEVE

ILKEIANVLNFSIQYSFDPSPGAWGRLKPNGEAVGAFLKIVERKSDLMIGSLTRTKNKSY

FISFSSVITFNGIFVVIPPGAPFSAFEKLIRPFDKSVWICLMIILSLGFFIVIAVAPKPR

SLMRKIIIGKEVKMPAMNIIVALVGGSQHILPKRSTPRILLMSFLLFCLVIRTLYTAALF

KFLQSDNSKPTLSSIKEVIDKKFTVYVDPTYYDNFWEMKILKRSQVVNASLFYDYLMKTS

DPNFKGVVLILMDELSHINKKNYKKFTLRVLSEHVITSHSCWAFPKDHFLVEAFDEKILA

FRKNGLLNFLAGKYMDPKYITIKRPKIGPRKLNLEHLEGGFKVWFCGIFSALFLFILEVL

TSQFVKIDCCPFTIKY

>IR7.x18_CLUMA_CG004581-PA

MLKFSSKIFFLLYVVDITSSFNINLEIKRELPSSVSLSEIIEEFYIRNALDLDIITCSND

GEGTNSIVEFILRNIHGSISYRIEDCKNLKDSTKRRFVLIIMDHLNTDLMIEELTPNRFD

YSGFYLIYYIDNQSDNDILHYLFKKLSDLFIYNVNVICNDSSGIQLRTFFFFNEGSCNST

DPVVINKFENNRWISKDFFPRKMRNLFGCPLKVATFIYEPAVIMQGSYDDNNYTLVGSEV

EILKEIANVLNFSIEYSFDPSPGAWGFIKPNGEAVGGFFKIVKKEVDLMIGMLGTTYTRN

IYVSFSTVIIFNRVFLVIPPGAPFSAFEKLIRPFDKSVWICLMIILSLGFFIVIAVALKS

RSLMRRIIIGKEVKMPAMNIIVALVGGSQHILPKRSTPRMLLMSFLLFCLVIRTLYTTAL

FKFLQSDSRKSPVSSIDELIAKNFTVYHYASMEETLRHLNIKKLLQLVNVTEFSSYQERT

FDPEFKGAVVSLMDQILYLNKKNYKKNYFRVLNEVVYTSLSSWVFPKNSFLVEMFDENIQ

KFRENGLLYYMTNDFIDLRYLNIKEPKQGPRKLNLERLLGGFQLWFLGISIAFLIFLLEA

LCGYSKTFLKERKNQLYVVN

>IR7.x19_CLUMA_CG004583-PA

MLRYSIKVVVVLAFCTEIISSSSNKEFIRKTRLHDALSQLQESFFPNKTLDIITCSCHDE

QANEIIEMFVRKAHGSISYRIEDCKNLKDSTKRRFVLIIMDHLNTDLMIEELTPNRFDYS

GFYLIYYIENQSDIDILHYLFRKLSDLFIYNVNVICNDSNAIQLRTFFFFNERSCNSTDP

VVINKFENNRWISKDFFPRKMRNLFGCPLKVATFIYEPAVIMQGSYDDNNYTLVGSDVKI

LKEIANVLNFSIQYSFDPSPGAWGRLKPNGEAVGAFLKIVERKSDLMIGSLTRTKNKSYF

ISFSSVITFDSVLVVIPPGAPFSAFEKLIRPFDKSVWICFMIILSLGFFIVIAVALKSRS

LMKRIIIGKEVKMPAMNIIVALVGGSQHILPKRLTPRMLLMSFLLFCLVIRTLYTASLFQ

FLQSNNSKPTLSSIKEVIDKEYMVYVYEIYYDNFKDIKLMKKSKSINAAIFHEYLMKTLD

PNFKGVTLNMMYNTIYINMRSNNTSFLKVLNEKLLTVYSSWVYPKDSFLVESFNEKLEAF

RGNGLMEFLKLEYVDPKYLAVKETKNGPKKLNLEELRGGFGLWMIGNIITVLLFLLEVSY

FSLRFSLMNN

>IR7.x2_CLUMA_CG003826-PA

MKVFSFLCLCLFYDYSLSSERFIRKENNYKNLADAVYDVVNEIFSDGYAAINVMLTNKNS

KNVMDFTSEFFRIIDKHPKVPTRYESIDKLGQVNRRRRFNIFVAGSFDDFMRIYNRMTSA

LFWFNGYYLIVFVDGEIPELKEVWRLLWKIQIYNANAIFYKNDENILVKTFMPFTKKNCH

NTSPLLVNEFKNKKFSKSVKDFFPNKFGNLHGCEIRAAISDESEPAVFVAHTKHNKLKVS

GRDIRLLQGLSEVMKFSLNFTYVGHSGYFFANGTSKGPLKALLNKEADLSISDWYLKEHR

AAFFQNSVPYFSEKLVFVVPPGKPLSPIEKLIYPMTLHAWILLLTCYSFGLVVIFVVGRS

SIKYQHFVFGRDKQKPYLNMFAAFIGVSDHALPKRTFARFLLMMFLIFSLVIRSFYQGSF

YQLLKSNKHHLKVQTIDEMIKKDFTFYVYSGLSDLFQGTEGIKRRLVKISIEDKISYANR

ILNNPEFLGALGDSMTMIAFHNQKMSEDFRYLICHDVVLIAPIVMYTLKDFYLLNEINSN

IDVFTAAGLVDLWHYRDIDKQALKIKSFDNPKVLSLIQLTGCFQVLIFGYSISLIIFMGE

ILLRSFSNTTLINYMEA

>IR7.x20_CLUMA_CG004585-PA

MLGVPSKILLIFYVVDIISSFNINFGTKRELPSYVAVSEIIEEFYIRNALDLDIITCSND

VEGTNSIVEFILRNIQESISYRIEDCKNLKDLTKRRFVLIIMDHLNTDLMIEELTPDRFD

YSGFYLIYYIDNQSDNDILHYLFKKLSDLFIYNVNVICNDSSGIQLRTFFFFNERSCNST

YPLVINKFEKNRWTSKDFFPRKMRNLFGCPLKVATFIYEPAVIMQGSYDDSNYTLVGSEV

EILKEIANVLNFSIQYSFDPSPGAWGSLKPNGTAAGAYLKIINKEVDLMIGTLSKTETKS

YFISFSSIITFDSVLVVIPPGAPFSAFEKLIRPFDKSIWICLMIILSLGFFIVIAVAPKS

RSLMRRIIIGKEMKMPTMNIIVALVGGSQHILPKRSTPRMLLMSFLLFCLVIRTLYTASL

FKFLQSDNNKPTLSSIKEVIDKEFVVYVYPTFYDHYKGMKILKRSKAINVSIFHEYLMKT

LDPNFKGVALNLMYNTIYINMRSNNTSFLKVLNEKLLPVYASWAYPKDSFLVESFNEKLE

AFTENGLMEFLKLEYVDPKYLAVKEPKNGPKKLNLEELWGGFGLWMIGNIIAVLLFILEV

SYFILRFSLMNIYSFIIVSYYYLI

>IR7.x21_CLUMA_CG004588-PA_pseudogene?

MLGVPSKIFFLLYVVDITSSFNINLEIKRELPSSVALSEIIEEFYIRNALDLDIITCSND

VEGTNLIVEFILRNIQESISYRIEDCKNLKDSTKRRFVLIIMDHLNTDLMIEELTPNRFD

YSGFYLIYYIDNQSDIDILHYLFKKLSDLFIYNVNVICNDSSGIQLRTFFFFNERSCNST

DPVVINKFENNRWTSKDFFPRKMRNLFCPLKVATFIYEPAVIMQGSYDDNNYTLVGSDVK

ILKEIANVLNFSIQYSFDPSPGAWGRLKPNGEAAGAYLKIINKEVDLMIGGLTRTVAKSY

FISFSSTITFDSLLVVIPPGAPFSAFEKLIRPF

>IR7.x22_CLUMA_CG004590-PA_pseudogene?

MFEILIKLILIIISSNSIACHASKYKQTHENSHDEISQVIDQFFSTRSAYFNVIKCIKND

DVAGMIIGTLLFKTHGSISYRIEDCKNLKDSTKRRFVLIIMDHLNTDLMIEELTPNRFDY

SGFYLIYYIDNQSDIDILHYLFKKLSDLFIYNVNVICNDSNAIQLRTFFFFNERSCNSTD

PVVINKFENNRWTSKDFFPRKMRNLFCPLKVATFIYEPAVIMQGSYDDNNYTLVGSEVEI

LKEIANVLNFSIQYSFDPSPGAWGRLKPNGEAVGAFLKIVERKSDLMIGSLTRTKNKSYF

ISFSSVITFNGIFVVIPPGAPFSAFEKLIRPFDKSVWICLMIILSLGFFIVIAVAPKPRS

LMRKIIIGKEVKMPAMNIIVALVGGSQHILPKRSTPRILLMSFLLFCLVIRTLYTAALFK

FLQSDNSKPTLSSIKEVIDKKFTVYVDPTYYDNFWEMKILKRSQVVNASLFYDYLMKTSD

PNFKGVVLILMDELSHINKKNYKKFTLRVLSEHVITSHSCWAFPKDHFLVEAFDEKIFAF

RKNGLLNFLAGKYMDPKYITIKRPKIGPRKLNLEHLEGGFKVWFCGIFSALFLFILEVLT

SQFVKIDCCPFTIKY

>IR7.x23_CLUMA_CG004591-PA

MLKFSSKIFFLLYVVDITSSFNINLEIKRELPSSVALSEIIEEFYIRNALDLDIITCSND

VEGTNLIVEFILRNIQESISYRIEDCKNLKDLTKRRFVLIIMDHLNTDLMIEELTPDRFD

YSGFYLIYYIDNQSDIDILHYLFKKLSDLFIYNVNVICNDSSGIQLRTFFFFNERSCNST

YPLVINKFEKNRWTSKDFFPRKMRNLFGCPLKVATFIYEPAVIMQGSYDDSNYTLVGSEV

EILKEIANVLNFSIQYSFDPSPGAWGSLKPNGTAAGAYLKIINKEVDLMIGTLSKTETKS

YFISFSSIITFDSVLVVIPPGAPFSAFEKLIRPFDKSIWICLMIILSLGF

>IR7.x24_CLUMA_CG022132-PA

CPLKVATFIYEPAVIMQGSYDDNNYTLVGSDVKILKEIANVLNFSIQYSFDPSPGAWGRL

KPNGEAAGAYLKIINKEVDLMIGGLTRTVAKSYFISFSSTITFDSVLVVIPPGAPFSAFE

KLIRPFDKSVWICLMIILSLGFFIVIAVASKPRSLMKRIIIGKEVKMPGMNIIVALVGGS

QHILPKRSTPRMLLMSFLLFCLVIRTLYTAALFKFLQSDNSKPTLSSIKEVIDKEFMVYS

YDTFFENYNEMKILKRSKAINASIFYEYLMKTLDPNFKGVTLGMMYNTIYINMRSNNTSF

LKVLNEKLLTVYASWVYPKNSFLVESFNEKLEAFRGNGLIEFLKLEYVDPKYLVVKEPKN

GPKKLNLEQLWGGFGLWIIGNIIAVLLFILEVSYFAFMRIYLKYSSKL

>IR7.x25_CLUMA_CG010954-PA

MWNKVFLILIALQSIEASKYLEPVKKPSMFLRPLKYIIEKYHDKFWENKHYSTVILFHKY

PNHNHAIEDEVDNILTTIGAKIPIKVKGFSKDISTKTETRNLNIVFCQSVYAYNDFYAMI

LAKQFNPHSLFILVVTFDTTKYDYLFRMFDLFWKLSVSFIDILTISPDNPNDILAYTYAP

FVGSCNNITIGTLNSYFAIHDKWISDDFFLPITKKIHGCPVRVATFDYPPYIMVKNDSNG

KLTVDGIEGNILNTLSQMRDFTTEIILADKKWGEVYDNGTTTGIIELVVNRKADIAIGYL

AISHERESLMTSGCVYYSSNLMWTIPPGEKFSPLQILMKPFQSPVWICFFTVLVLAFVII

GILNFFPTKFRHFVYGRNVRTPGLNVVNITFGGSLHRIPSRNFARTIFILFTFYCFVINN

SYKGSLFLFMQGDYTNPPVSSTDELIKKDFQFYMIPEAKGFANDLHILKRTVYIKSQKDI

LELYGNLSDSKFKGAMMNTEEKWAHKNIESSPNNYYHRTKDVIYQQNLAIYRNKVSLFRW

DFDNVVTRMLESGLINKWSKRFTDSDNLKKRDEQRKLPLNFEHFKGVFQLLGVGFLISFI

VFIAEIFLHKLKSR

>IR7.x26_CLUMA_CG011029-PA

MKYLCCFLSFCVMRLSNGQSHFHIPKYNNGIIQATVDIIEKFYMDRTNTLNFFYASLKQN

ETSSINNIDIINEILYQTREKVVVQIENSWKLKNSEISNCKLVKRVNNIFFIDSYNSFEN

IFRFITPDKFDYQGFYLIVITNYADEQYQVAKKIFNYLWKEFITNVNVIWMPDESENEAI

VFTYFPFSQFFCGKVHPMKLNHYRDGNWLQNYSDFFPKKLYNLHNCTLTVAVASSPPFMI

LKDDTDGKLSIDGIDAKLLKELSNQMNFNINIVQVDTQGGVFKNGTAYGAAKLIINNDVN

LTIGYTTCTSSENKHMSSSHVYYTSKLIWIVPPGRLFQSFEKLIKPFQRSLWLCVMLVLL

ISFISIIVVKCYPKRVQDFVFGKGITSPSLNMITVFFGGPLYKLPKRNFARTLLGCFMVY

CLIIKSTYTGALFKFIRSDARDKDVSGVKAMIKQNFSFYVVDSSHDLVHEIPKILNRSIF

IPRTMLSEIHVHLLQPEFKGALLSSEEHLSYLNMKSFPEKYYHHSKQIFYVFNLCIYLHR

QSCFTEEINQHLLKFASNGLIDVWASQFIDKKCLTEKVSTLPTQLTNEKLLATYEILFVG

FLMSFIIFIAEMSISKISKRLQTKL

>IR7.x27_CLUMA_CG011031-PA

MLKKIFILLLLHSACGKLSDFELPLDNGRKSHTITQIILDHFINISETINVVYTFSDANI

STFEGIDTIMMELEQSATLTLEHYTKIHPNMTKQSHNVVLCESIESFNSFLSTINPKYFN

FQGYYLIVIPGRLYEYMSDMFATLWSLHIVNVNLLPLTRDDDDFIYTYYPFTSLDCGSTL

PVQLDYYRDGKWMEKNELFPNKMKDLFNCTLSVVTFDIPPFIKIDKEKEDSPSGIENCGE

KHLNMKNQLESPIKMIKDLKANLTVGYFITSEGRDEHMKSSDTYYTSNLIWAIPPGEEYS

SIEKLLMPFESTVWICFLIVLIVAFIVIKVLECCHQNIKNFVFGRNIKNVTLNVVNITLG

GSLHKVPVRNFARTLFILFTFYCFIIQNVYRGGLFQFMRMTLRKPLISSTEELVNKDFLF

YMYPTAYNVLKLDILKGRTRITDPKGLDRLLDKSTDPSFKGAMLTSEAHLANKNIEASPD

NKYFYSTKDPIMTQNIAIYMHKESCFSDQINLILKGIINGGLFNKWVKQYTDTDALMHKA

SSPEGHRPINVDQLLGAFQLLGALLFISSAVFLIEIILRKMKKN

>IR7.x28_CLUMA_CG011032-PA

MWKKTLIIFLLHSACGKLSDFELPLDNGRKSHTITQIILDHFINISETINVVYTFSDANI

STFEGIDTIMMELEQSATLTLEHYTKIHPNMTKQSHNVVLCESIESFNSFLSTINPNYFN

FQGYYLIIMPSRLCEYMSDMFATLWSLHIVNVNLLPLTSNDDDFIYTYYPFTSLDCGSTL

PVQLDNYRDGKWMEKNELFPNKMKDLFNCTLSVVTFDIPPFIKIDKEENDSPNGIEVILM

SVLALKMNFNIKWIYSEELWGEIYGNGDVTTGKYFKFVEGRDKNMKSSDTYYTSNLIWAI

PPGEEYSSIEKLLMPFESTVWICFLIVLIVAFIVIKVLECCHQNIKNFVFGRNIKNVTLN

VVNITLGGSLHKVPVRNFARTLFILFTFYCFIIQNVYRGGLFQFMRMTLRKPLISSTEEL

VKKDFLFYMYPTAHNVLELDILKGRTKTTDPNGIRDLYNESTDPSFKGALLSSEAHLAFR

NIEASPRKYFYSTKDPIMTQNIAIYMHKESCFSDQINLILKGIINGGFFNKWVKQYTDTD

ALKHKATNGHRPINFDQLSGAFEILGFLMFISLAVFLIEVILKKIKAQKNK

>IR7.x29_CLUMA_CG005593-PA

MSFSAKKIIFILFHLSVVPSAFCGFEEVTRISPHKLIESVMELLENVYYRESSTVNIIST

SEHSKDLVESLLREISRNMSVNVVLESPSSIQRNNRRLCNIFLFQSHEEFMKIYEKLTTD

SFVFQGFYAILLTNERKVEVDEIFKSFWMKQIHNVLIFYFKDFQVKVQKFQPFNEKKCND

TKAVDVDMTEIKASFSSGLRDLKKCLIKVSAPHIPPFIMTNEKNEIVGRDIDLINVIAEV

LNFKIELQYLNYSLAFGILYNNGTATGAFKDLLESKSDLIIGDYYLTQSRLNFLDASSAY

FSSEIGFVIPPPMNLKPIEKLLQPFQLNFWIPLMICFCVIFFIAFVMRFVTSRYDENVMK

NISLEMNLVALIFGVSYPSESRKIYLRIFLISLVIFFLVVQAAYQGSLFKFLQTNSKHKQ

VQSVDDMVERDYKIYSIGTTDYVATHPKIKERIVKVTYPESRNILRKLRNPTFQNAYINS

LNQLLIVNQRNDFNFTFSWCKEIIVTIPIVIYFRKNSFLVNSVDSVVNKLETAGLIDYWH

FNYLDKRFLKIKLSQGEARRLNVEHLSGCFQLWGFLILTSSLCFVFEVLYDIYIKRKTRI

GNLNSFL

>IR7.x3_CLUMA_CG003827-PA

MKVLIYFIFLISRHISCLTIESVTDELTINLTLAVDEIISNVLSQHSSSVVLISPDNCKY

DFKDFTNHQLMKAFEKAKVSILFETSTSIKSESIDPILCGVFTINSFDDFLEIFRKISEN

NIIRHDGYFLIVLANGKIPEIEMIFKLFWNIQVYNVNIMYQQKENRFVTVKPFMPFNKLN

CNDTSAITVNKFVKGKFVKNLKYFYPQKMRNLHDCYVRVAVANSAPPFVIEKKLSNGSSE

LSGQDISMIKTIAEYLNFKINYSFIGHEGYLLENGSAVGPLKTLIDGDSDLSVTNWWMIP

VRLKFLDSTAPYLVEPVAFNIPPGQDLTAFEKLTFPFSTGSWILIGSCYLIGILVIFIVK

RCSDDVQNFVFGLNVRHNYLNMFSVFMGVSTSILPGRNFARFLLMMFLIYSLVVRSIYQG

IFFLLLQSNRQHKEVQTIDEMIEKDFTFYAYPGHYQIISKFDKIRDRSVILEFQDVPSFI

KRIKTDASFKAAHALSYLELIYKNQQNMSGLQLHICKEFLTSNIPVVVYTRKKFYLLNAL

NKKLDILKAAGLTEYWNFQPINKRLLTLKEIELPKVLTLNHMLGSFYILFLGILISFAVF

MLEFIHCI

>IR7.x30_CLUMA_CG015226-PA

MMNKNLILILIFNFHNEIFCEISNLEKKSDGHLLNSALLEIIDTFFATRTSTINFVICSE

HPKKAVSMLEYVLWKINVPINLETCGRLNSFKREYVIMLIDNENIDKMIKDLTSQQFDYS

GYYLIHFLNGNNKKNLEYLFKTFLKLFIYNVNVVTSHNDQNIQLLTFLIFTEKSCNSSDP

VIVNEYKNLKWNINLFYPPKMRNLHNCTMNVAGAIYEPAIMAQGDYENDNYTLIGVDVEL

INEVADVLNFSTKFIYESTPGGWGKINEDGDATGCFLQLMKREADLMIGFVARTEIVVVI

PSGAPFNAFEKLFQPFQSEVWIAFGATFVLGIILVTLITLKKNKMWKNILIGEESKTPVM

DIISTIVGGSIRVLPRRTTARILLVAFLFFCLVKRSLYTSALYKFLQSDSRKPAVTSIQE

MIDNDFIIYYYPAFKQNYQGSKIFVIGEEIDQSEVSAYQRKTLDARFKGAVVNYDSQVLY

ENKLNHKNYYFRVLNEPIVIALSTWSFPKDSYLLKIFDETMSALRENGLIDFISSKYIDS

YYLRIKEAKSGPKKLTFPQLSGSFDLLYFGLLIALILFILEIISVKLKNKFIKNIINLLI

>IR7.x31_CLUMA_CG005439-PA

MLFYVPNMSKETMMAIFKMSYFRWLRLNSFLVKVKGKLVLKSFSRFSQPNCRTQKLKAIN

YFSETNLKWSSSQFFLDKIDNFQGCEIIFGGFFRFPTAYGELQENKTIKITGYHVDLINI

IAPYANYKVNISFFKKVSSGRKKNISPDVFISSLLLHGTRNREHNFNTPTQAIHYELIRI

IVPPRESYTSFEKLLLAFDKPVWSLIIITFALAFIVIIILRFCPRFIREFVIGRNVKEPA

LNVIIAFFGLGQTILPRRNFARFLLIMFILYSLIIRTAYQGKSFEFLQKDMKRKGVQTVK

DLFDKNYTIYTNKFLQTEWKTADIIKHAKPVKSFVTVKKMVQEISTNSSSHSAFIMYENH

VKIIFAVLNRNNITTPIHTLDEIIAVAPKSMSFVANMELFKTFDEKFVRLYN

>IR7.x32_CLUMA_CG005442-PA

MDFYIIILFILINPSNSLIERKEILSIALNSIVKNHFNKNNERFDIVSEAENEFYFDDVI

NGILNATNQTVKITKSLSSKIKTEVINESTIFIFQNDDIFLNNLRHMKFGKNSRNIHMLF

YVPNTSKDPMMAIFKMSYFRWLGLNSFLVKVKGKLVLKSISRFSQPNCRTQKLKAINYFS

ETNLKWSSSQFFLDKIDNFQGCEIIFGGFFRFPFAYEELQENKTIKITGYNVDLINIIAP

YANYTVKISFSKRRLRKRRFDVFISPPLLLHHVQKRHHFNMPTQAIHYESVRAIVPPRES

YTSFEKLLLAFDKPVWSLIIITFALAFIVIIILRFCPRFIREFVIGRNVKEPALNVLIAF

FGVGQTILPRRNFARILLIMFILYSLIIRTAYQGKSFEFLQKDMKRKGVQTVKDLFDKNY

TIYTNKFLQTEWKTADIIKHSKPVKSSATIKKMVQEISTNSSSHSAFITYENYVKNIFAV

LNRNNLTTPIHTLDEIIAVAPRSMSFVANMELFKTFDEKLTQLREAGIMDHIKKPYYELY

KEKPNNDPKVLTMDDLSAGFVVFLVFLLISIASFSLEIIIDKLTMLFNKYLKGCVINK

>IR7.x33_CLUMA_CG005443-PA

MNFNILTLFMLINYLSANFIYKDSSKNHFSTAINGVVENYFNKHNERFDIISAAENEFHF

DEIVNGIMKTTNQTVRITELLPDEGPIIAINESTIFLFENDDVFISYLHFIRFSTNKRNI

HILFFVPNMTTTTFFLINKKLGIPCLRLNSFLVKVKGKLVLKSFSRFSQPNCRTQKLKAI

NYFSETNLKWSSSQFFLDKIDNFQGCEIIFGGFFRFPTAYGELQENKTIKITGYHVDLIN

IIAPYANYTVKISFSKRRLRKRRFDVFISPPLLLHHVQKRHHFNMPTQAIHYESVRTIVP

PGESYTSFEKLLLAFDKPVCNLIIITFALAFIVIIILRFCPRFIREFVIGRNVKEPALNV

IIAFFGLGQTILPRRNFARFLLIMFILYSLIIRTAYQGKSFEFLQKDMKRKGVQTVKDLY

DKNYTIYSVKSLQTEWKTADIIKHAMPLGSLAAIKKMVQEISTKSSSHSAFIMYENHVKN

IFAVLNQNNITTPIHTLDEIIAVAPKSMSFVANMELFKTFDEKLTQLREAGIMDHIKNLT

KYKLHKEKPNNDPKVLTMDDLSAGFVVFLVFLLISIASFSLEIIIDKLTMLFNKYLKGCV

INK

>IR7.x34_CLUMA_CG005444-PA

DEGPIIAINESTIFLFENDDVFISYLQIIRFSTSKRNIHILFYVPNLTTTTFNLINKKFV

VPCLRLNSFLVKVKGKLVLKSISRFSQPNCRTQKLKAINYFSETELKWSSSQFFLDKIDN

FQGCEIIFSGYISPPAAYEELQDNKTIKITGYHVDLINIIAPYANYTVKISFFMKRQRKT

KLDVFISPLLLHDVQKRHHFNMPTQAIHYESVRAIVPPRESYTSFEKLLLAFDKPVWNLI

IITFALAFMAIIILRFCPRSIREFVIGRNVKEPALNVLIAFFGVGQTILPRRNFARFLLI

MFILYSLIIRTAYQGKSFEFLQKYMKRKGVQTVKDLFDKNYTIYTNKFLQTEWKTADIVK

HAKPVKSFATVKKMVQEISTNSSSHSAFIMYENKIKIIFRMLNQNNITTPIHMLDEIIAV

GPKSIKFVANMELFKTFDGKLTQLREAGIMDHIIKPYYKLHKEKPNNDPKVLTMDDLSAG

FVVFLVFLLISIASFSLEIIIDKLTMLFNKYLKGCVINK

>IR7.x35_CLUMA_CG005447-PA

MNFNILTLFMLINYLSANFIYKDSSKNHFSTAINGVVENYFNKHNERFDIISAAENEFHF

DEIVNGIMKTTNQTVRITELLPDEGPEIVINESTIFLFENDDVFISYLQIIRFSTSKRNI

HILFYVPNLTTTTFNLINKKFVVPGLKQKSFLINEKGKLVLKSISRFSQPNCRTQKLKAI

NYFSETNLKWSSSRFFLDKIDNFQGCEIIFGGIVSFPAAYEELQKNNIIKITGYHVDLIN

IIAPYANYKVNISFFKKVSSGRKKNISPDVFISSLLLHGTRNREHNFNTPTQAIHYELIR

IIVPPRESYTSFEKLLLAFDKPVWNLIIITFALAFMAIIILRFCPRFIREFVIGRNVKEP

ALNVLIAFFGVGQTILPRRNFARFLLIMFILYSLIIRTAYQGKSFEFLQKDMKRKGVQTV

KDLFDKNYTIYTNKFLQTEWKTADIIKHAKPVKSFVTVKKMVQEISTNSSSHSAFIMYEN

KIKIIFAVLNQNNITTPIHTLDEIIAVAPKSMSFVANMELFKTFDEKLTQLREAGIMDHI

KKPYYELYKEKPNDDPKVLTMDDLSAGFVVFLVFLLISIASFSLEIIIDKLTMLFNKYLK

GCVINK

>IR7.x36_CLUMA_CG017661-PA

MCPRKSHLILLLCFAITEAFKGISINKGSEANLISAIKFIAEKQFQRDWPTVNILFSSNQ

RMKNILNDLVNDVLTSNEEKRSIQLSDCGNITKQAESLRTQNIILLSEISSFRTFESNLL

PSLFDFKGFFLISLIESSSSDLEEIFTAMFKKNIYNVDVVIDNCGKISLYTFMPFNSNKS

CGNTQPVLIETFNNNEFKTFNLFPNKFRNLQNCSIKVITFEDSFSIFKVNESDGRFDLKG

SDIDILYGLSRSMNFHPNITFLDTFEPWGMVAKNGTVTGALGELVNKNAEIGIGNYFLKA

NRIEILDHSISYNSLPLMFIIPPGEELSGFKKLLQPYSIEVWILLSIILVTALCVILIIN

FYLKGLRSFVYGHQVKNPVVNLTIIILGSSQRKLPKRNFARFLFIMFSFFCLVLRSIYQG

SLFIFLQSHELEKNVENLFEMYERGFDFFIYSAHFDILENYPNIQKRRVKFINTKKFLDK

PLKSSNKAVIMMPLVEVIASNKRHFNDFTHKILKEPMRVDNIVIYFSKHFYLIEAINEKL

SQLIASGIIKHWIENFLKLQYRMEVKEKKGPKILNLNHLSSVFNLYSIGVAISLISFIIE

NFQFYLAERFKRNVVG

>IR7.x37_CLUMA_CG017663-PA

MMKKVLIFLISRCLFNYVVESENHQKNLFNFHQKHSSFPVFKILETFYCKESSVVDVICS

NESEEILDAFNSVIAHFGSCTSFQFDSLNNVSRLHKRTRTFNVIILESFESFNILMNNIS

NFDFDFNGYFSMFFIDIETIELNRVFEFAWKYFMHNFIVLTGNGTKITLSTYDPFRNGIC

GDTSPVSTVIDIKTDIHNSIDLFPNKMINMNKCPLRIPLINYLPAINFESYRDDYIITGI

EGELLLTMKDLLNFTIKVLQLKGEQYWGVIEKNGSATGAMRMVIEGEADLIMGTYAMTLL

RLQYMDSSLSHLGFPFVLVIPPGRKFTPMEKLLKPFQMEIWIAILTFLGFLFMSILIIKK

GNIKARKLTLFGETFRLSYLTMIDSFLGGSFNLLPSKTSLRFLFASYFIFCLIVRNLYQG

LLVQILQSEDRAKPVKTVDEMMEKGFNFYMYDSYLEHTEHLKFSSRRKVHKYNDNEKYLK

MTLDPNFKGGVCSSLDEVAFLNKKNYKNFSYLILPEDVYLFKYVIYFQKHSPYVKKFNEK

ISLFKSSGLLEFWFSKYMDLSYVHVKEPKMVPRTLNIEQLKGGFILWLCGCSISLFVFIL

EAIYSVVIKYLL

>IR7.x38_CLUMA_CG022136-PA

MRLCGVVINIFGLSLNFLGVKCDRNQITDDINHGRKSEFQFSELLKTFYCSNTTVVDVIF

SNENEEILDAFNSVSVHFGSCTSFQFDSLNNVSRLHKRKRTFNVIILESFESFNILMNNI

SNFDFDFNGYFSMFFIDIETIELNRVFEFAWKYFMHNFIVLTGNGTEITLSTYDPYQNNR

SCGNTQALSSVFNANTDSIKLFPDKLANMHKCPLRLPKFEYYPATFYEEIGNETMLKGID

GDLVMTMKGLLNFTLDFVSLENPDEKWGSITENGSATGAMKMVVDGTADFTIGMYVISPL

RLKFMDSTISHVSFPFVLIVPPGQKYTSFEKLFKPFSWNVWITVGVTFLLSSLIILFVKR

SNNQKLKYFFFGAGYNPPYFYIIDAFFGGSLQTLPKSTSPRILLSFFLFYSLILRTIYQG

NLVKILQSDDRSRPVMTVDEMIENDFHFYMYPTFIEHSRHLKFFSRRKIYPTIENEEYLL

KTLDPYFKGGVCCTLDEVAAKNKQNRNNYTLRVLPEDIYLFKNGIYFRKNMPIVELFDKK

ISLFHSSGLINFWVSKYLDLSYAKISVTSETPKKLSIEQLEGIFLLWLYGLLTATTFLVL

ENIYSKTLINVKETIRTIVGKRM

>IR7.x39_CLUMA_CG017667-PA

MIVTLVILAFANRCYGTFTIDNLQPMSSDLVDAASEIISNHFKSNAVTINFISALTNETK

CERSFLINNLIAQCDVVFVEDIDVITKRHRLYNVIFIDTFQSFLRLFRRLTSKYFVIDGY

FLMIFINGPIDELGEITKSLWNNFIFNVAFLAQTEYELNISTFIPFSKPKCDNENCIQNC

GDTKPVVIDVFNKTFTKNKDFFPNKMENFFNCPVKVVTFNCPPMMMIKYDEDKNFDFKGP

DGEMLKLLSKKLNFKIDLIHISDLIRWGALYENGTSKGAIAMVINKEADLTLGMYTITYL

RTRFMTSSHLYYSVPFIVIVPPGAPLSPFEKLFRPFQIDVWTLLLITFATAVGVVTFVKF

QSPRVRNFVFGAGNRSPYLNILNAFVGGSLTSMPTKNFARTLLMMFFLFSIVKRTLYQGA

LFQFLQSDDRSKEVQSIDELVEEDFDVYMMASSLEHTQNMKFRDQRVVVTGTVLEERKRE

TSNPRSKFAVTSSFEQILYFNKMNYKTMTLTVCKEYLFTFQYGIYMRKNSYLEQAFNKQI

SSYKTSGLIDFWASFFIDMKFFNMKMSYDGPRKLSIEQLLGGFELLFFGYVIGFIIYIFE

ILSKRMRLRRLQRIIEFFT

>IR7.x4_CLUMA_CG003828-PA

SYAQQPLIFIIPPGKPVTHLERLISAFSLQSWICIGLFYLIGSLVILFVQLKSPCVQEFV

FGAGLTSPMFRMFSVFIGDTLNNLPKRNFARFLLMNFIIFTLIIRTVYQGSYFRQMRTFR

HHKEVASINEMIENDFYFYAHEGMIDILLGFPAIRERVRELNSEDLPEFLKEIRTNAYFK

AAYADALLQTDYLNFKNSNESQHICKEAFATNLPVVIYSLTDFYLLNALNEKILEMIKAG

LIDQWKRESFRQNVRHVQESIEPKVLTVDDLEGSFLILMFGCLLGFIAFLCEKFLL

>IR7.x40_CLUMA_CG017673-PA

MFGHKRVLIVLLLIQSSVAFEECFKIKHSIINLIKHAYSSQKIYFFVDGSQMDLLNSVIS

SVTDVSLQIENINEIQLQKEKRRYIIALFDSFESFVLFNEVLSDQNFDFKGYFLIISSNI

TSKEIDKIFETLWKKFIYNVDFLSCEDIEIQLKTFIPFSNEKCFDTSGKIINIFDDEKLQ

WKTNEFFPEKFKDLHNCPLRVTSFEYPPLIIVERFENGSFGFSGRDINVLEGLADVVNFQ

IDMIFLPNYGDWGISYENGTATGSQKQLMDNETDLVIGGFSLVYFRTFFLSNSESYLTTA

RTFVIPSGRAFSALEKLLIPYNFFVWIAFLLCLGIAVIVIITAEQRQSKLRSLIIGNEIK

TPFMELLIVIFGSSSHVLPSKNFSRYLLMMFLLFCLVMRTIYQSGLFKFMQSDKRKQGAS

TINELMEQEYDFYLFEFAFNILDDDSKFMKRAKIIEPSEMFEYYEKTLDPNFKGGVYALL

DEIAYYNMKNIKSHTFRVCREPIVTAQLVVLLQKNSYLQEAFDQKLILLKASGLIDHWIS

DYYNTKYINIKEAKKGAEPLSFKQLIGAFQVWIFGLCLAILTILIEFIFKYKKNVVLCLR

KTFCK

>IR7.x41_CLUMA_CG017674-PA

MTRSASLSSHEKLSIYTSNESAVLQELSQAVTKIVKSLEGDKPSATHIIKVTTHKADAKS

IDPFIETIATTANVLLIVSKIMSNRKMEKILLSCSGTSQQKFLIVLLKPFGNSIKLMLDI

MWRKFILNVHVVTLEANGDVALYTYFPFTKDFCGQVHPVVWNIYRNGAFVTQREHFPRKD

KNFFRCTLDVAVFNVPPFTLFINESTNFDVSGVDGNLIKVLSVELNFTINWIIVSDDLRW

GEIYANYSSTGATNLVMNRKVDLSIGMWSRTSRRTKALSFSSITYAQGGLILIVPPGAEI

SSFSKLLQPFNPSVWYFVIATLFIVVIATIFLNYSPRKVKDFVFGKHIRTPFLNIIIVMI

GSPMHHVPGRNFSRWILTLFILMWLIIRNLYQAVLYKELQSSERNRHVESLEEMIRQGFH

YYMIAPTIENIIHLPEVYERRIIVSRVESDLIVRGKFNDPTVKAGFVAGFHTLLYINKVN

LYGFHLNFCKEPLLLRQFGIVFPMNSFLVSSFDEKLIILVENGLIEYWLMENTESDGFAL

PEVREPMKLTLNHLLSAYQVLGLGLSLATTTFGFELIVRRINFLQKCL

>IR7.x42_CLUMA_CG005842-PA

MLKVVCGEIQIEVEVKIEEKNFSSATVEIIRTFFVHESSNINIFAIYEKHSMNDILIGII

SSTDSSTTFSITSSDDNIFHEAIDKKVAIIIVDTLTSFTKFQDTFLSCKKIAKNGFFLII

FPNATSQDLKKMFLLLWENFIYNVASLTIDENLIGMFTFFPFSQKLHCNNAFAVKINEYN

GQHWMSEKFYPEKFKDLKACQIRVGTFETEPSVMRKVVNDNKTKVYGSEVDVLLGMSDLM

NFTPIFHFYIKSSGDIYENGTSTGLMNKLMRKKEDVVIGFLSLMYSRAKFLSATTAFTYE

PLAIVIPPGGYFTAFEKLYFPFSTFVWILLILMLIATIFVVLITRLCFNNLYAFLVGDNI

KTPLLNFFLIVYGLAQHQLPKRNFARYLIMNLMLFFLIIRNYYQGVMFQFLQTELRRPDI

ASIDELIKEDFNFYVYKTLESRTKGHPFYNRRVVIEMNEIEKIRNKTLNPKFKGALFNYM

TQVFYLNQLNYKEFSYRICKEKFLSNPMVFYFQKDFYLVNEFNDKMNDLKENGLINLWIS

KYVDKRFTNQKETLVGALPLNVHHLLAIFELWIGGLVISFIIFILEHSTVKHFKLFLSR

>IR7.x43_CLUMA_CG007066-PA

MKWKISVLLIIFLIALMKSTKIQNVNQSSVLSHAISDLILDNFSNFKLPQFHYIKAIKNF

NENHVNDLISEIFFMTSNISATRTIESFSQSTNHQIFPVILFVDSIDSNKIQTRKCFIIV

LINKIGNYEMKIIFSSFWKMMTENVNLVMKNDDENSDLFTFLPFNEKNCNDIRPVKINTF

NSKIKKWKSRKFHVKKTKNLFGCPLIVGYAAGTSDPATMICNDSKGNLELAGIEFDVVLE

ISKRLNFTPKFDMSTQYSVIPLIMIISRSDEYGDFEKLFRPFTADVWLALGIIILLALII

IIIEELSSEKIYNFLIGDKVRTPVLNLTAITLGVSQKNLPRRNFGRFILMSFSIICLILR

SIYEGEIYNTLKSNERKPDVASIDEMIEQSYKFYIYETLESRVKDFQFYKNRFVYPNEEI

DHFRHMTLKSSTKIALFNYLDQILYLNAVNYKNFTYNICKEKFMTNSFVFYFRRNHYLVN

DFNRKLEQMLKSGIIQHIKFKYFDPNFI

>IR7.x44_CLUMA_CG018285-PA

MWKKLYLFLLLHLTIGAFINFKIPNYSTALARAVIDLIDNYYATKSHTIHMFCTSIENES

SSESHYVMNEVIRQIDKKVAIDVLENLNSINNGSILANKKHKNVDNVFFCNSQKSLFSLI

NNITTKTFEYDGSYAIVIIGYVGDIYEFMSEIFQNFWIKHIVNVNVLWKTSDDQNEEAFM

FTFYPFHSSECGDTTPVQINTFHDDIWAFKAHAFPNKMKNLYGCPLQITLSHQFPFIYWK

DGSLNGTIEDLEGVEADLLKVLSETMNFTSNLTMFDEEISWGEIYDNGTSTGPVQAVIEG

KANFTMGFFTELPERDKYMKASHVYYTSELIFVVPPGKFYTPLEILLSPFQLTVWIFFFI

AILLALLTIEVLSFLPLEVRNFVIGRNVKYAGLNVTAVVLGVPMHKLPTRNFAKTLLILF

TFLCFIINNSYRGSLFKNMQKTLTKPPMASNEELFKTDFKFYIYYSDYLLIPELTHIIER

SISLDDNEFNLKYDDVTNPDFKGALMSMKDYVKRRNKLKHRNGKTHLRSINDAIFQLNVV

TYLNEESNLVHEMNEIILRLIDGGLIKEWAKRAFGEDIGSYPKDDTTEKALSIKHLMGSF

YLLGSLLLLSTIIFIAEISIVKLKK

>IR7.x45_CLUMA_CG008428-PA

MWNKFLFIFVLQLIFGTAEAFNHLEPSEHSLPITQPLIKLVSDVMEIHQRYRGLNIFYFD

PKSSKDDVEIYLRNILCNFHGKFVVQLEDYSKPILPKEYDKLGRKFNIVFVQSIVTINAF

AKTLRHWNYDSSSFFLIVFLKPFGYETFGGMSTLSKHFWGVNIVNVEYMINYDKLGVVET

STIFPFMKLARSADCKSEFGLTGRYDISVDKWIVKNYFQHKNFHMKGCPLNVATFSYEPF

MIIKKDGNDELTLDGIEGTLLNVLSEKMNFTPKITLVEEKRGFLYDNGSASGAMEIVMSN

KVDLTLGFFVYHPKREKFMTSSKIYYTSKAIWAVPPGDSFTPFEKLTKPFQSAVWICFLI

IFILALLIIEIINFCPKRIQDFMFGKNIKTPKLNVVKIILGGGLSKLPTRNFARTILIMF

IFYCFIMSNSYTGGLFYFLQGDLRNHHMSSTQELLKNDFKFYAMSHVKTLLNDTPTILGR

TTFIDDEEVYDRMINNLTDSKFKGAVMIPEENLAYRNIRASPNKYFNRANDDILLYNIVI

YMNKFSMYQASIDRVLQNLISGGFINMWVKKFTDVEYLKNRHESRELALNFDQMQGVFQL

LIIGLFISFIVLMLEIFIAKLKRRRNNVN

>IR7.x46_CLUMA_CG007258-PA

MKIFEIFFICSLSLWSVKRLPKTASATIIYQDVLASRKLLAISVHEIINKVFPKHGTSND

LFYSINDFIIKDFIEELFAIHPRLIFRHGNFTFLPKDLTRCVVLVIKSFNEFLEFYSSIP

STSIKSNGFYLIVFVNGRIKEIGEIFKILWKIDFFNVNVMFADKDELIKVETFIPFTEGN

CNNTTPKLINEFVNGTFNQGIDEFFPKKFKDLKGCFLRFTLPNDSEPFIIPKVFINGTYN

TFEGSEMMLGKVLAEALNFQINITYAGFFGYVLDNGTAVGPLKELLEGRADITVGAWWLK

LNRIKVLDMTSSHMSETLIIIIPPGQQLTPFEKLIFPFTLPSWILIIVSYFCGFMVVILA

KFRNKSVRNFIVGSNIKNPVLNFYAACLGVSNHVLPKRNFARFYLMMFMISSLIIRTLYE

ASFFLLIHSNKHHKEMQSIDELIAEDYHFYMRTGTFDFAKTMKKLEGRSSISEYEHEEES

IKRIREDPNFKGVFIHSQIEVLYKYKKRPSEAQPTFCKELFLSNLPTVAYTRKNFFLIDS

LNEKIELLGAAGIIYYWNRETKKNPMENAEVNVALNIYHFLGCFQLLLFGFLLSFLTFLK

EKFTNRCK

>IR7.x47_CLUMA_CG007926-PA

MRKLVFLIFVIQIITKVTKAYNEHPDTRYWTKISHPLIYFVHNDIFERYKGRFNILYYNS

PERSEMMEVQDEINILLTQLSGDIIYQLEDYSEPLPKKEKNPLGRRNNVVFCYTRESLLS

FREKLNSENFDSNSYFLIVSKPRDLLEIPKVLSVLYTSSWATEIPNLLMMIHIPGNPDVI

LYTYFPYQTNVCRYLRPHVINSYDLQTHKWKNHKFYGSKTHNMSGCPLRIATFNFEPFMM

VNKNDEGKLIASGIEGDLLNFLSETIDFTPEIILADEKWGEIYDNGTSSGATEMIVNKEA

DIAIGYFISHPNRESVMFSSLVYYTSNAIWAVPPGEMYTALEKLMKPFDTSLWIGFFIVL

ILAFLIIEIINFCPEKIQDFVYGEKIRNVRLNVVNVMLEVSLPKIPARNFARTVLILFVV

YCFLMSNSYKGSLFNFMQKDLRNPPLDSTEKLLQNNFNFYAMSASETSLNATQNIFKRTS

FVDDEKIIEEIYDNLTDSSFKNALLVPEESWAYKNIHSESTINYNRAKDVMFQQNMVIYM

NKLSIFKVELDKRLRELVCYGFIDMWAKKYTTSGFSYTDSTQTTKQLNLEHLEGAFLLVA

VGWFLCIFIFLFELFYFKLKKLLKKN

>IR7.x5_CLUMA_CG003829-PA

MKIQILVTLFIISINYVCAENQSRYSILAEALEEYLSSRASTRTASLTLERDGDNFQATY

EVKDFLDEFLKKKFTIPKIGIRIETFAVPTERKRVFRIFIVSSYEGLTRILNERSLKDIL

LNGHFLIILPKCENHDFEKFFEFLWNLRIVNINIICAKTNQSVSVFSFMPFTQNNCRDTK

PILINQYQNGKFIKPSNFFDPLKIKDMKGCPVRVALTNKFGAVSYSATDKNKKRISKPII

KLISTLSEALNFKLNFTYMGYKGFFFENGTSEGPLRVLLDENADLAVGNWWLKTNRLKFF

SSTVSYAQQPLIFVVPPGKPVTHLERLISAFSLQSWICIGLFYLIGSLVILFVQLKSPCV

QEFVFGAGVTSPMFRMFSVFIGDTLNNLPKRNFARFLLMNFIIFTLIIRTVYQGSYFRQM

RTFRHHKKVASINEMIENDFYFYAHEGMIDILLGFPAIRERVRELNSEDLPEFLKEIRTN

PNFKAAYADALLQRYLNFKNSNESQHICKEAFATNLPVVIYSLRDFYLLNALNEKILEMI

KAGLIDQWKRESFRQNVRHVQESIEPKVLTVDDLEGSFLILMFGCLLGFIAFLCEKFLL

>IR7.x6_CLUMA_CG003830-PA

XXVASINEMIENDFYFYAHEGMIDILLGLPAIRERVRELNSEDLPEFLKEIRTNPNFKAA

YADALLQRYLNFKNSDESQHICKEAFATNLPVVIYSLTDFYLLNALNEKILEMIKAGLID

QWKRESFRQNVRHVQESIEPKVLTVDDLEGSFLILMFGCLLGFIAFLCEKFLL

>IR7.x7_CLUMA_CG003831-CLUMA_CG003832-PA

MKIQILVTLFIISINYVCAENQSRYSILAEALEEYLSSRASTRTASLTLERDGDNFQATY

EVKDFLDEFLKKKFTIPKIGIRIETFAVPTEHKRVFQTFIVSSYEGLSRILNERSLKXXX

VFIGDTLNNLPKRNFARFLLMNFIIFTLIIRTVYQGSYFRQMRTFRHHKKVASINEMIEN

DFYFYAHEGMIDILLGFPAIRERVRELNSEDLPEFLKEIRTNPNFKAAYADALLQRYLNF

KNSNESQHICKEAFATNLPVVIYSLRDFYLLNALNEKILEMIKAGLIDQWKRESFRQGVL

NVQESIEPKVLTVDDLEGSFLILIFGCLLGSIAFLCEKFLL

>IR7.x8_CLUMA_CG003833-PA

MRIQILITFLIISINYVCAENEGRSSILADAVDEYLSTRVSRSTANYILERDGDSFQTTY

EVKDFLDEFLKKKFTIPKIGIRIETFAVPTERKRVFRIFIVSSYEGLTRIFNERSLKDIL

LNGHFLIILPKCENHDFEKFFDFLWNLRIVNVNIICAKTNQSVSVFSFMPFTQNNCRDTK

PILINQYQNGKFIKPSNFFDPLKIKDMKGCPVRVALTNSSEAVFYTSMEKNKNKICGPDI

KLISTLSKALNFKLNFTFIGHSGFLFENGTSEGPFRVLLDENADLAVGYWWLKTVRMKFF

SSTVSYAQQPLIFIIPPGKPVTHLERLISAFSLQSWICIGLFYLIGSLVILFVQLKSPCV

QEFVFGAGVTSPMFRMFSVFIGDTLNNLPKRNFARFLLMNFIIFTLIIRTVYQGSYFRQM

RTFRHHKEVASINEMIEKYFYFYSNPGMIDILLGLPAIRERVRELNNEDLPEFLKQITTN

PNFKAAYANALLFTDYLNLKNSDESQHICKEVFATNLPVVIYSLTDFYLLNALDEKLITI

IKAGLIDHWTRESCRQNVRKVQESIKPKVLTFDDLEGSFLVLIFGCLLGFIAFLCEKFLL

>IR7.x9_CLUMA_CG003834-PA

MRIQILLTLLIISINYVCAENESRSSILPDAVEEYLSTRVSRGTANYILERDEDNFQTTY

EVKDFLDEFLKKKFTIPKIGIRIETFAVPTERKRVFRIFIVSSYEGLTRMFNERSLKDIL

LNGHFLIILPKCENHDFEKFFDFLWNLRIVNVNIICAKTNQSVSVFSFMPFTQNNCRDTK

PILINQYQNGKFIKPSNFFDPLKIKDMKGCPVRVALTNKFGAVSYSATDKNKKRISKPII

KLISTLSEALNFKLNFTYMGYKGFFFENGTSEGPLRVLLDENADLAVGNWWLKTNRLKFF

SSTVSYAQQPLIFVVPPGKPVTHLERLISAFSLQSWLCIGLFYLIGSLVILFVQLKSPCV

QEFVFGAGLTSPMFR

>IR75l_CLUMA_CG013518-PA

MEIFRIFLIFIFTSATRGASIEESIKVIKHLLLSLNTSLTISVISCWDCDYEIKLIKTLN

MQNFMVSVQQEQDIINKSSFPIHQHNNFIVLDLSCENSKQLLNELTPNEFFQRKFILLDT

QSKINLQKYLEFKAILPSSEIFYFENDVGNIFVKQVYRTSLESSTNIETFGLWFKGNDAF

KDLRSTTVTSRRRNDLYGTELRASIVVTNNDTLHHLDDYHDKHIDTISKVNYQLTNRLIE

WLNATISYSIVTSWGYKNNETNEWSGMIGELEKKIADLGASPLFFTADRVDVIDYIACTS

QTKSKFVFRSPKLSYTDNVFVLPFDSGVWKSLGAMLMISSFILYFASSTEWKSNLVNVND

PTILKPSMYETSFVLFCALCQQGSSSLPISFGSRLITMICFTALMFLYASYSANIVALLQ

SPSSKIKTLEDLYNSRIKLGVDDTVFNHYYFAHADEKIRKAIYNDRIKRKDGKENFYNLS

DGVKMIREDLFGFHMEVGVGYKILLEEFQEDEKCGLQEIQYLQVIDPFYAIQKNSSYYEL

FKIGLLKLHEFGLQDRENSRLYTRKPKCSGQGSKFISVGLIDVQPALLIFLYGIGAGFMA

LFIEKFHFRFKSLVKN

>IR75p_CLUMA_CG015435-PA

MARFLVIVTIALSLNLVLCDNNENKNKISFILDFLKSQIRPTYLIVWRSCFNDDEKIELI

KDSFVFTSFYENSSLNESEFNENPQYWLFVTDFTCTNASDENIREVDSKLFAHPYRWIMF

VENNKILHHIKALVDSNVILAETTNDGFGLKQFYKIDEESNEIYFENYGSWDSKSGIVDE

RSTKIISRRRENLHGKLITSSYVALNKSSRNHLADFVDKSVDSILKYNYIMVNSVLDKLN

VTKKELFQDTWGYYNVKTKNWSGMIGDIVYNGADIGGTGLLIVADRIPYVDYTPLNTPTW

EKFIFRAPQLSSVSNIYTLPFRSNVWFSCLILVLISTILLYLTSHTDKSDVNNENKRFTD

SFLSTIAAICQMDPSLSSSVTSSRIIMFFIFLAFSLLYAAYTANIVSLLQSPSKSIRTLE

DLYNSKIELGFEGTPYNRYFFENADGPLEKKVYKKMAPPGEKDHSLKIHEGVARLRKGLY

AFVAEANGIYSIMEETFYEHEKCELVNIEYVKFSEPFIAIKKKSAYKEIFKVNFILAMES

GIQKRTFSRIYTNKPVCVNSQNFQSISFSDALPAILLIPYGILLSFIVLVIEKLLHVFNF

RRELCLRI

>IR75q_CLUMA_CG012625-PA

MIRCLVIIMLTLSRNLVLCDNNGNKNKVLFLLDFMKSQIKPTSMIVWRDCFDDVEKIELV

RNSFKLTSFYEQNPLNESDFKENPQYWFFTTDFTCTNESEEITRKIDINLFSHPYRWIIF

VENNEVLRHIRALTDSDVLIAQIINDGFNLKKFYKIEEESSEIYCESYGSWSMQSGIKDE

RIMKVISQKRENLHGKLITSSYVALNKSSRNHLTDFADKNVDSILKYNYIIINSILDKLN

VTKKELFQGTWGYYNVKTKKWSGMVGDIVHKGADIGGTALFMVAERFPFIDYTTLNTPTR

GKFIFRAPQLSAVSNIYTLPFRSSVWFSCLILVLISTILLHLTSQTDNSAVNSEKVNHHK

RFTDSMLSTIAAICQMDPSLTSSVTSSRIIMFFIFLAFSFLYAAYTANIVSLLQSPSKNI

RTMEDLYNSKIELGIEDTPYNRYYFAAAEGVFEKKVYKKLNPPGEKDHYSKIHEGVARIR

KGLYAMIAEETGIYNIMEETFYEHEKCELVNIEFLKFSDPFLAIKKRSPYKEIIKVNLIL

AMESGIQKRTLSRIYTKKPICVNRQNFQSISIDDSLPALLLIPYGIILSFAILLVEKILH

LLNFNREICLRI

>IR76b_CLUMA_CG022135-PA

MAGIAFLLSVICGNSPDVCISDQNSLGVNYNVIQTSPPSYKEQMDKIKEELEGQTLNVTT

LEDSPLSVLKRVDKGEYDGGGFAFEFFDHLSKKYNFNYNVIKSEYNIVGSSNDTEGSVLQ

LIRDKKVDVAVAFIPMMADLVEHVRFSTPLDEGEWKMIMKRPQESASGAGLLAPFDEVVW

YFILVSLIVTGPCIFGIIWIYHRFGKHTGEPYYNLWQCVWYVYGGLMKQGSVLSPNADSI

RVAFATWWIFITILTSFYTANLTAFLTLARFSLPINEPKDLSDKSQRFICHRGFAVNYAI

TNYDEELGILRSMLENKLGELVNQNNDTEILLNQVYKKNVVFIRDKPAIDHLVYDDYRKR

KTLPSERLQCPFAMSKIVSLKRKRGFIYPKDSKWAKLFDQQLRYMVEGGIINHRLNYGLP

KAEICPQNLGSIERQLGLNDLFTTYLTMLIGFSSALAIFVAEMIMRYFNGRKDMSMRERG

IEQSNTRQILVKPFRKTKASFIQESTKTISPPPAYGEAIKTIFNTQASVDSELSNSGSIE

GYRTLNGRKYAIIKDENGTHLVPTRSPSAALFNYQLAYNSYN

>IR8a_CLUMA_CG021272-PA

MKIEVFITFIILITLGKAQDEKNQSTTLTEKNIKTLEIVFLHHANHSKFMSAVDQIMEQL

QNETVAINYHKISYDKSKFAVSNHREICKLGSNGISLFIDATFNGLNFTVRDYLKRNNVP

YFRFDFSIQSFVKIMQNFLVARGALDAVFIFQDSETAEQALYSLLSVTSIKIIVLDQHQP

NVISRLKTLRPTPSYYAIIGDTVNVSRIFQAAFDFGLVSQLPDRWNLMFTDYDDEKFTFS

GYPEMSRLLLDKTVCCNPKCICSSQNTINFMAFKSFLHQILLVLEGHDYPIQRFECNKES

DKSVRAEKLLEDLSMTIRKNNLIYFDDDSKIFVNLKYNVKTNVDNSSVVATTAIVNQNGV

TSIGNNRIKVSKRFMRIGVTESAPYTFIKRDSKTKKILRDGNNHKIYEGFCIDFIEMLSK

KMNFTYELVEPTSGKFGKKLADGNFDGLIGDLERGETDFIIAALKMTAEREERIDFVAPY

FDQTGIRIVMKKPIPDTSLFKFMTVLRLEVWLSILGALSVTAFVIWILEVFSPYSGRNWS

YGEESRKFTLRESFWFTLTSFTPQGGGEAPKALSSRVIVAAYWLFVVLMLATFTANLAAF

LTVERMQSPVQSLDQLARQSRIKYTVVKDSDTHKYFQNMKYAEDTLYELWKNMTLSSRND

EARFRVWDYPIKEQYASILMAIEGTKPLPNASEGFRIVNERVLSDFAFIHDANEIKYEIS

RSCAFAAVGDIFSEQPYAVAVQQGSHLRDEISRFILELQKDRFFEDLTAKYWNSSNKGSC

LTSDDYDSEGITLESLGGIFIATLIGLVIAMLVLVGEVYYYKRKSGKVKEMNMTKPVIFT

TKIDPLHDINISVDRKIMKNSVLLGHGEFVPVEKRKSRLSFSPRIHHD

>IR93a_CLUMA_CG020350-PA

MTQFDSVTIEKSLCDDFPSLLTANASIAIIIDREYLDNKYENILNEIKVIIERVLREDLK

NGGLIVSYYSWTRINLKKDFTAVLSITNCENTWEIFEDSRSEYLLLMAITDPDCPRLPYD

EAIMAMLLYDNTFDRDMISRCVNALSRDFPDESGDLVKPLSVSIYRIKDSPHEWDRRKMI

RTMLKQLPTKFIGKNFMVLVTAKLMQSLMEIARDLKMVDTFSQWFYVVSDTSFENHNISF

ITTLIEEGNNIAFILNHTRGGNECVSGIECHSNELLKSFVLGLSKAIREEAAVYGQISDE

EWEIIRASKKERRNDILNFMLDNLRKTSKCAACTTWNVKSSEIWGNQYKYSAFSDGFLKQ

VSSDGRDSKETSNGLKMIDAGFWKPFDGLKMTDALFPHVSHGFRNKQFKVITYHNPPWQY

VINNESGIVGMPSGVVFDILNEVSKKLNFSYVIHLAQVSTATNITDANGTVTKDIHLLTT

DIPTEVLSLLSENHIILGAVANTINERYKRLINFSVPISIQPYSFLVAKPKEISRIYLFA

APFTTLTWLCLAGMIVILPPVLCIVNRLSPFYEFHRHQRTLGLFKIDNCFWYIYGALLQQ

GGLYLPHADSGRIIIGSWWLIVIVLVTTYCGNLVAFLTFPKIDNQIKTVDELVKHHKSVT

WGMRGGTYLEDYIRDTDIHKYQLLYSGANFYPDENEEIIENVRKGKHVYIDWRSNLKQIM

RREHLKTESCDFSLSFEEFMEEQIGIVFPMNSPYLDLFNIEITRLHQMGLIERWMKQYMP

QKDRCSKQSTVIEVINHTVNMDDMQGCFLVLLFGFSAGFFFFLFEGILQYYRKFREKDII

EPYVD

>OBP1_CLUMA_CG022072-PA

MKVLAIIILCFTLSISAQTHKKSIAFKAIRKCAKEHEIDYLSASNFIKGDFSKANQATKC

FLKCVANDMEFWINEKPQKTNMMNYVDLLVKNSDGVSDIIDSCIENDVSDDFCEHLYNVY

KCFWSKAIVPTNQKLQLEKEFHAENLNDNELKKEASQS

>OBP2_CLUMA_CG022073-PA

MKFCVAVFLVSVAFSTVAGQIIRRFTVRQATRQCIAETGISYDTAVKFSKGDFSEDTESA

KCFLKCFAYKIKFVSPEGEFQKEELLKYVNLVASDSIPAGEIIDTCKNDTKRETDECDFI

FRAYKCFWDGVRASEANAARFSIYNTFEFDRLQDENETIPNESEIIPKQIEDEAKNVNE

>OBP3_CLUMA_CG022074-PA

MKLLVLALFCTFAFVNGSPNTLATKLEEFRIAFQGHIQHCAQEIGVTKDVVLRMTIGDFS

VNDQKTQCFVGCFFKRVGFMNEIGEPQEDVILTQMLSLGLKKDVVKPIVDKCVKIKGDDD

CQTAYEIFKCSWPGQFAAKH

>OBP4_CLUMA_CG022075-PA

MKIIFLVLSLFSLIAAFQKDEIKEAQDRHSFLTKITKECTEETGLSLDDAQKLLLGDLSL

KTNEAMCFVRCIFQREGFLDTADEPQVGYIAESLAESSKLNRKALLKILKKCSSFKYKDP

CESAFKLLECYMNNKIIRKNVEL

>OBP5_CLUMA_CG022076-PA

MKFIFGIFLVFVGAKALTEEQKLKVDEHTKICAEEVGLEYESGVKYRFSVIEDNTDSAKK

FVKCFFIRTGFMNEDGVLQKDVMLEKISLGKDDEAKAKISAVIENCTALSGETADETAFK

VHNCYWGHILAAKAAAEKKDS

>OBP6_CLUMA_CG022077-PA

MKISFALTVFGIFAVASAMPFTEEQKQKAQNYIEKCIQETGVSPEIVQKLKAGDFSNEEE

KTQNFALCFFREAGFVDDQGNQQLDVIIEKLSAGNDTEQAKAVVEKCKDVTGTTPSNKAF

NAYKCYRTAMQF

>OBP7_CLUMA_CG009075-PA

MKLFIVAALLIVAVQGDYVLKTLDDLQTARKECILENKVPQELVAEYQKRIFKEEGVTPC

YIRCIFTRLGLFDEKTGFITENYLKQLGRGDTVKDGVVGCYDNTGTDTCLWAYRAFTCFT

KQGFLPEGY

>OBP8_CLUMA_CG022079-PA

MKTELLLTKYFIGIIIIIAAFHISNGHQQIRLRRLKFAVYNIEDCAEEIGIDEENANKLI

MGDMTIDSDEAKCFLRCFYKKVGFSSQDDQPAQINFIAQSLKDCVTLKDEILKRIAKKCV

ELESENECEKAFEFHRCLFKEASKYNDEL

>OBP9_CLUMA_CG016340-PA

MKWILICLLIFCCGFASDILALRCTSSDGLSVEDMRKGKKLCMRKVSESDLETTNYDEYE

NSDDNDDNKKIYSGTSHFEATEHSNTFNNNTNPQINQHYNSKTGGNDYNPQQNDQRDANP

YQYTYNDNNKLNIRNHNDGHRNDSNNKQERDKACVLQCFFQELKMSNDEGFPDKHKALHV

LTRDLRDRELKDFYTDSIQECFRMIDTDPKLHRDNCLFSKALIICMTERAKMNCSDWQNS

IGEGVFI

>OBP10_CLUMA_CG014270-PA

MNILFVNIIVVCFLVGFIIGGDPACENVPKTKIDECCKIPKAIDDKIIKDVSTNVQAMEG

TPTFKKCKMSEEIFKKLNLVVNNDIDCNAGSQFAENSMSDPEWKKVMKEAFDGCCIEAPK

LAEKYQQLVQFPKDQCNILFDVIIDCVRLVSFATCPKSSWTDSQVCNDGKTFTHKCGNEL

EAMEKFMMNQYA

>OBP11_CLUMA_CG014539-PA

MEFQLLTVFVVLSTLQFDLTLSQNCNEVPTFNISDCCKFPDIASEKMVHKIKQELAKTPN

QSKYLKACRFYEKLFKRCDIVNSDNQIDKAAARKYFDAQIADDDWKQIFADAVDECIDDI

NDEDFPTNSRCDVRFIAAYECISLYAFRNCKDSDFTDCNQCNTAKDFAENCDGTRSMSRY

LNNQICDCL

>OBP12_CLUMA_CG014673-PA

MSIFFNNHYDHYNREKIFKVPIIIGCTGLCLAFSYLTVGFLFQGLNRECCRVPDYVTGKI

INKIHYELVKAPAMATYLKACKLFEGVFRKLNLLNGKEIDQEAARKLFDIQLKDPEWKYL

FSRALKSCLDDVNSQLDDDFIEVAAKDDDKTDEKSISTLRKDFCDNRYTATVECLRVISY

MSCKHIDWDKNPTCHKAKEFLENCEHDAEAMNRFFGFQRYLY

>OBP13_CLUMA_CG010580-PA

MKIFIVIACLACVVKATTMAEHKQMLMGIAQECKANEDATDDDMGRLVEKKPPTTKEGKC

LFACIMEQMDVIQDGKLSKEGFLEFASVIIDGDAGKMKVAEELANECGAIRDDDRCELAY

KAGGCVKMAGMKRNIDFGF

>OBP14_CLUMA_CG022081-PA

MKFISVLVVFMCAAYAKGDKDMMMKIIETCKGVTGASDADIARFITHAPPETNEQKCLFA

CFLTSLNVIKDGKPVEESFIEMIKTVTGGDAEKMKIAADVFAKCSGLVAGKDPCDDAVNF

GMCFMTEGKKHGIPLQI

>OBP15_CLUMA_CG022082-PA

MIFKSFFMLVLILFVDAEIHSGSEYDMVEKLFGDITSKCKGIENATDEDVAFMFVEDDSW

PETYQGKCFIDCFFEEIGIFKNHKFHKRGFLTTVLMIADIDDDESVEIEKLGDMIKTINK

ECGATTHADRCENALDFAKCCYHIFEHEFNDDSNEVDAVKE

>OBP16_CLUMA_CG010583-PA

MMFLHLIILGLICVTEISAVAEDTLKEIFLSMINDCQIQEKGTRDDSVSVLAGKIETPEA

KCLVTCIYETLGILSKGVVKKPSFMQAGKMIFKGDPEKMKIVSKTVDTCKSIAGERCQQG

ANFHKCVVDTYAANGVVLPTYTLD

>OBP17_CLUMA_CG022083-PA

MLDMRVVQLFILSAICLYKVSAVSQDNVKEIFMSMLTDCKVQENGTDDDMAGVLVVNFKA

PTVKCMVACMYESFGILKKGTFKKNAFMQGSKTIFQGDPEKMRISAIVADKCNGISGERC

EQATNLFLCVHNSYSANGVNLAEYVV

>OBP18_CLUMA_CG022084-PA

MRSIFLIVCLSLITVQVFALSAHKKRAFLEAVMKECKLEENGSDADYESIMAEKLSETGP

GKCMMACGYEVVGLFDQGAFNRGAFLHIARIIVDNDETKLKEAPGIADECGKITGERCQQ

AYDFSKCLHDYVKKTNADVHLF

>OBP19_CLUMA_CG010585-PA

MNCKLSMKILKYVALLLMICGVTFVKSDDREEKKKMAKEMMFAMSEDCREQEGATEADLD

VLLENKNPTTKSGRCLVACLQEQFGVVDNLKFVPEAFVSVSAMAIKEDEEKMKLVKEVAQ

ECKDVTDVDRCELAMKISDCLRNALKKRNVEF

>OBP20_CLUMA_CG010701-PA

MNLKLLQTLLRKAFTTMKKCGNAINHSISTLELNDTGSFPDEVEMTPMCFLKCYLESIGV

LDKELQINRVKAVELYNLNDDSETYDDCADDMSSENVTDECEKAYFFVRCVMSRKLFDNL

EEDEMV

>OBP21_CLUMA_CG021576-PA

MSVRCLLSAKHTILISSFLTLCCYFALAQLSQTDPNCINYHPPLSVLPEQCCMMPDFFDD

ADLIKCKTSHGTESLIRDVINRRKRFATTRIELGICYLNCVFKLADIISDDQIIDVERFK

EKLTSETPLESNSIDTINNSITKCVNDLDSGVIKAHDHSTYNCSSIPSSLMMCVHMNFFL

NCPSNRFNLTDQCIELQDYLRRCPITL

>OBP22_CLUMA_CG022085-PA

MNNFGVFCLFIALPLIACGVDAKPNDKHMEMAKALLTECKATEGGSDSDLEKLISDSDPG

TRSGHCMVACANEKIGLIKDGKLNKNGFLEAAKIVTSEANHVAKGAEIFSECEQTLNSIT

DRCDLAVAFEKCFHDGAKARNLEDFLH

>OBP23_CLUMA_CG008109-PA

MSTNAIYIFGFFIIICGVNCAKVTVEQMKQATEPIRKVCITKTKVGEDALSDLRSGVVQD

KKELKCYVNCVLEMIQIIKKGKLQYDAVMRSVDTMLPDELKDDTRRAVNVCKDVSVGIKD

FCESAATMLKCIFKENPNFFFP

>OBP24_CLUMA_CG008104-PA

MKAFIIYFICFGVVFINQTAAQCDKLPEEMDLEECCNFPDMSFDDAIDKIVDQLKDQHLD

GFMMACKVSESLFKELKLTKGTEIDDAATQKYIDTQVHDPNWKPVTKAAVTDCLKYVSEK

KDAIAKILEAAPFNIKKDKCNASFVAMGACYHFKVYQSCPKDQWTESKKCSATKDWIVKC

SNNMDIVKAFGSLNKA

>OBP25_CLUMA_CG005306-PA

MKAFIIFALFAVAYADFKIATGDDLNRYRDLCKTELTIPDEDIEKFKKWDFTSERSPCYI

NCVFRHMHLYDNETGFQIDNLVKQLGQGRTDNIRPDIEKCIDNTVTDNCQRAFKGFQCFG

KNNLQMIKSSVN

>OBP26_CLUMA_CG001935-PA_two_domain

MKFFICFIGLTTLAISGFEHTDVSEKTEKIKEAEEFKKKISKECSDEIGFSFEDGLKLLH

GDLTHRDDKAKCFVHCVFKKEGFINEQNQPEKDFIVNHSKDIVKFSLDDLTKVIEKCSKF

PHENPCENAYELLECYHEEEAMRKAEESKNLVMKIAKECSNEIDFSFENGMKLLEGDFSD

QTNEAKCFIHCVMSKEGFINQNNKPEVDFIVNQLTAKYKIPREELKNVVEKCSKFSSDSK

CENAYELFECFLTNDAIAKADAAKKRVD

>OBP27_CLUMA_CG022086-PA

MKFLIVLLALIVVVVCDDDDKLKKANENQKILEKYAAECSEEIGFDLESANKLLVGDLSF

NSREAKCFMHCFFKKEGFFNENDELQKEFIVESIATIAPVNKENLTLIVNTCSKFKTEDA

CENAFALLECFTRATTNPPKKEEL

>OBP28_CLUMA_CG001383-PA

MTSVHHVRWMVLIVVAVFSTDHDYSQQMKCFKDSETLRSCCHLDTISFPYSDECEESFER

LENSTGKYRKMNATAEHLLCIFKAKHIIKHKSCNVSENHLMTAVKEKYAQTDYNEAAILA

VDECVNYIGELKTRLNRSGDETCYYLPFALNFCITRHIADNCPESKWSNSEHCDNTKSNA

TLYKDCLRKW

>OBP29_CLUMA_CG001384-PA

MMSAVGFIFLVLSLTGLTNCNNSLGQKCASDTTMMKECCPIATSKSFHIKECDETFKDLE

SASESNKLRAFHENLECIYNERNIFEIDDCYTKLDEYKSLMREFYNEPELQKILSDGADK

CMELMNKGTRKHLKAGREDVCNVIPVYTYTCVAKYIASQCPPRYWTKSKKCETTRANLTP

VEVCFQS

>OBP30_CLUMA_CG000708-PA

MKSLLIFLCLIAVIAAEKGKDGRKSRGEIPGCENPPKTELSECCSNFPKFFNNGMMDNKC

KKTCRPKKKNDHKCCMVECSMEKLGLVTDNNFDASKAKEVLTKQLGNNEIWSSEVVENII

NECVSSAPTMVEALRDNVFKKGSKFPKCSDSTTLNMAIAHCIHRELFFQCPGLSTSAECS

ALVSYGKGCSKFPACKKRSGGRKSRKGRKRN

>OBP31_CLUMA_CG022087-PA

MMKIKVFVLIVIIAYASCGDDAMSKWMKMGECWKQTSALSKCCPLPQHKNMLSNEKCKQH

FDEKDEKDFKEQFKAKACGVDCIFKAEGLMNDNNVFDEAKIKEKFGAILTANDGGDFKDI

TMESIDFCLKRFGEKKEEWEKMAAEWKKKKGESADDSKCSMWPLSFVSCVRGSVVRKCPD

SKWQKNDECDGMKSKATPISECYQ

>OBP32_CLUMA_CG019521-PA

MNKFFVIFSLIAVALAELPEECWQGQPDGKSPHECCQLKEAIPMALFEKCKSLFPPPPPG

SHPKGCCMSECVLSETKIFAEGKMNKDEALKFLSKHFEGDAETIKVVHEAVEHCDKEYQT

KKAGFDEMAKQPAAPGEKVCNMASGFLIGCVNAQIFLNCPKDKVAADASCGPIKSFIDKC

GFLYPIKVVH

>OBP33_CLUMA_CG019503-PA

MKIWTASFICFLIIFDAKASAQCDKLPENLNLEGCCKFSIIAADDTVKKVLDEFKDQKLP

PAMMQCKMSEEMFKEYKLTKGDGVDKEAFLNHVENKVLEPIWKPIMKNAVEECYKGVSRK

TDEIVKELEDDPFNIKRDECNPIYMSIVTCIQLEAFENCPKELWANEKKCNDARTWITEC

GDNVESLKQIIKAKKSKH

>OBP34_CLUMA_CG022088-PA

MMIIIYKIRIVINFITLSHSSFIYEQKDNLAADCDKVPKGFNLAKCCKLPVLEKDYAVKL

ITDQFRNQRMSEVMFECKSIEAIFKEYNIAKDEGIDKEALLKTIEEKVLEPIWKPIIKAA

AEECYEDIMEERDEIVKELEMRPFNIGSDECNVIYMSLLTCIQLEAFDKCPQDHWVNSES

CNEARKWNEECGETVDSLNELGALKKS

>OBP35_CLUMA_CG022094-PA

MKIFYLCALFTVVYCQDVTPRRDDEWPPPNILRLIKPLHVKCVAKTGVTEEAIKEFSDGK

IHEDENLKCYMACIFHEIDVVDDYGNVHFEKLHDMLPEEMHEITLHMGKRCLYPQGENFC

EKAFWLHSCWKRSDPKHYFLP

>OBP36_CLUMA_CG015711-PA

MKTFALLFVLSAVNLCVSALSGKKNICNGRNVLKKDPQNCCKYPKLLFNLDQKKGCIKEC

SEKKENKEKYCFITCLLKDILSGEKDQEESDKVKFLTIFSEASEEKILSENWKTVIGNSL

DTCYEKISEVEARSGKILNCVFVENFLNCPSPVSEPQCDKMKEMLIDCSKTEHYRINKLI

PRFWKKEN

>OBP37_CLUMA_CG000709-PA

MKTLLILFCFIAVITARKNHGAGKKREATPTCENPPKTTIPECCANFPKPLIGGLMQTLK

IPTNSKFDASKAKEGLTSVLGENDAWNNVIIDKIFKECAASDSKNIMHIVNCIHRELFFQ

CPGMSTSDECKELVSFGKGCPKFPFHEAPLFRKIQE

>OBP38_CLUMA_CG022095-PA

MGHLSFMSLFLIFGVVIYLELTLFSRLVSGAMTMPQMYKMMETMRTTCSAKFKVTHEEMD

GLKVGKFDDNNKELKCYTFCVAQMAGTLSKKNEVSEKKSMAQIEKLLPEEIKEYMIGAVQ

ACKDVQKDYKEPCDRTFYTSKCMYEFDPKQFLFP

>OBP39_CLUMA_CG017963-PA

MKSVVVVLCFIGAISAQEKLPDCGAYPEKKLEECCGGIEKFIQPQFWGACEGECQHPDMC

CKVNCFADKLGIVKDGRFDKDTALGALAATLGDDATWTPIYTQAVETCIADVPQMKENFM

>OBP40_CLUMA_CG018049-PA

MKTVSIIFFFFIGAALTIAEIPNCGPLPAKRPIDCCEGLDKFIKREFYDECDKECSPMDM

CCKGNCFSMKLGILKDGAFDKETALKSLDDTFADPTWKAIYTKGLDECVARVASMKEQLT

PPPNAPKCDNVENGLIAFCLKKNLFMNCPMPSNDEECEQTRTFLNCVLP

>OBP41_CLUMA_CG020862-PA

MRPYFLFIFLNILYTTVLLQRLCSEKPPSDQNLKDCCSEFPNVIDLALIKFCNANFSSNT

QQQQQTIQNNQMPKGDCVSECITNSTKIYRGNGMIDRIHLARLLLNSVSGNREWSLIITN

SIAVCINETRIKADEFRQVTSMRPSFPNEILCHPISGYLLGCINTEMFRRCKNIAQSSDC

SNLQKYAENCHISMKYQEIKMK

>CSP1_CLUMA_CG016200-PA

MPKFAFYCVVAIILIAGCLCDETETYDVKYDNVDIDEILKSERLLTNYINCLLDEGPCTE

DGRELKETLPDAIQTDCSKCSEKQKEGSTKIMHYIIDNRPEDWERLEEIYDESGHYRTEY

LESKDAEESEESEE

>CSP2_CLUMA_CG018205-PA

MKLFLFVVPLIASFVAVTNGQFSKKLDNLNVDMILKNDRILSNYLKCLLDKGPCTSEGRE

LKVTLPQVLRSGCNNCSDKEKRNSRKVILHIQDKKPQDWAKLEKKYDPTGEFTQEFRRTV

QA

>CSP3_CLUMA_CG015020-PA

MKVLSYVICLSIVLLNFIPATNGQEEYYSSRYDSLDVDALFKSRLLNNYVDCLLDRKPCP

PEGKDLKRVLPDALRTRCGQCTKIQKTKALDVITRLYYQHPRLYTALAARYDPTGEYTKN

FENWFDEQNAVKPRPSDTIDNSRTTRIPSTWITTQTARITSRPTPRSTFRTLRTSVRTTT

QPQFRGNPIPTASLAAQTNPRRIETTTRFICIDGSTDPRCPPNCFINPNDLRCPRPTTTP

RRIETTSRFVCFDGSTDSRCPPNCFVNPFDPRCPRPTTTQPTTTTRRIETTTRFICTEGS

TDPRCPPNCVTNPYDERCPRSTTTTTLRTLPPTTTTERPSVVPITQPTFIQRSPATTVKL

ESPSILVELPKAEAPSSIRTIKEESSGFICQPRSIDQRCPPDCYPGSRDFRCPKATTTTT

TSTTSETPSTSSSISYRILPVSVRPSSASEPSFNQPTNVANKETFKPSTKASETSFRVSP

TTSVITEPSFNQPTNANTKETVRPPPKASVTPIRVPPPVTVRTSVITEPPFRRTTKIFTP

PPTPRLRIPFLPAIQRRPIEQQQPQPDFQPSLIDNFFIPNGNRPFRRSIDRIISRTGEVV

NNVAGMVRSTVKVIAG

>CSP4_CLUMA_CG015024-PA

MKVSLAILSLCTISLHLTPSTVAQSAEKNYYSRRYDSLDVNLIFRSSRLLNNYVDCLLDK

KPCPPEGKDLKRVLPDALRTRCAQCTKLQKGKALDVITRLYYQYPSIYTALAERYDPTGE

YTKNFENWFDEQNAVKPRPPIPQDQSQFDVPDNVQLGQAPGIENVKRDDISTQQAVRRSP

NIGNSNVRPNPNEIPTKRTRIPSTWITSSTTTTRRPTTTRAPLRTSSPVNRPITVRSPSV

TSRPPQQSPQTNFLFENIFAPRPEGPITKSINRFLDTTGKVVNDFAGMFRTTFQIITGTG

>CSP5_CLUMA_CG019523-PA

MKNFTFVHLFLLILIINQSWVYAQDDSPQNINKLLNNQVVVSRQIMCVLDRSPCDNLGKQ

LKAALPEVIVRNCKNCTKQQAQNAQKLISFLQTRYPDVWAMLVRKYRSKA

>CSP6_CLUMA_CG022151-PA

MKFFILTVCVILGVASAQRYTTKYDGVNLDEILKSDRLLNNYFKCLMDQGKCTPDGNELK

RTLPDALKTECSKCSEKQKQGTERVVRYLIKNKRSQWDQLQRKYDPENLYYQRFEKEAQA

RGIQI

>SNMP1.1_CLUMA_CG005205-PA

MKFDKEKLNNLNFKKIALISSLSLVGALIFSYGLFPPILRFVLKLKMQLKKGSLMRTMFD

VIPFPLVFKIHVFNITNPDAIMRGEKPIVADIGPYVFEEWKLRENWEEDVVEDTLTYDFK

NRFIFRPELSNGLTGNEIVQYSNLVLLGGLMAVKRDREAMLPMVTKALMSIFKNPDSVFM

RARVMDILFDGLKFECKGDDFTVDAVCSAIKSEAQGLKEIDETTLSISIFGSKNDTSFGR

FHVLRGVRNISELGQIQTFNGESMQSVWDGDECNKNIGTDSTLFPPFMTQDMGLWTFTPD

ICMSLRAHYLEPSSYAGMPTWLYSLDFGDFKNEPEKHCFCYDPPDDCPPKGTIDLMPCVG

VPVYGSKPHFLDTDPKLLENVGGLNPNRTIHDVFVNIEGISGTIFEGYKRFQLNLAIEPI

EDFEMMSKLPKVLLPLLWLEESVQLNKTYTNILKYQLFLGLKFNFVVKWMGIVCGTAGML

LSGFMLYQQQQQQKQQSPTTVSSATSAIDDDDKVNKNIANNVRSYSVGVSEAVK

>SNMP1.2_CLUMA_CG017212-PA

MWDETFPIILYVTGKLNSKFVLILFAILFGFVFVPKFLKKQFRNNLVLKPGTDLRKLWEK

APFAVTFKVYIFNITNADVVLMGGKPHVEQIGPYIFDEWKEKIDLVDNEEADTVAFDMVN

TFIFRQDLSDGLTGNEIVTIPHLLIMGGLIAVQRDKEPLLEMATKAMTTIFKPKSPFLTA

PVMDILFNGVGIDCSSEEFEAMSFCKALENEKPIKVVNDTYLKFSVMGGANATSMGRFEV

FRGIKDIMQLGEVVKFDDEEEVDAWDGECNEIIGTDSTIFAPFHDKKDILYAFAPDLCRS

LGAEYLKPSSYNHVPTGYYSMNFGDIKGDPRQHCFCRDQDVEKCPPSGTLDLYPCTGAPL

IASKPQFLDADETLAKNIDGLNPNRAEHDIFLHLEMMTSTPLSAAKRLQFALDCEPVEKF

ELMSKLPQTVIPLMWVEESVHLNTTYTGIFRAVYIFLSVNSFIKYSTMIFGILGLTFVVY

RIKNEMDEKKNLIQTVAAAVNKSASLTSAESVTKSAIMTAEKIVANGNA

>SNMP2_CLUMA_CG019584-PA

MSKWILVGPFVGVVLITVALIIGYVVVPPIVDQRVTENVQLVEGTEQYERWVEVPQPLDF

KVYIFNVTNVDEIQRGMIPKLEEIGPFVYSQSRKKHNIRFSRSLDRVSYYSQMSFTFNKE

KSGYLTEDMDITVLNMHMNSILQTVENETPFFMPVINDQLHTIFGPTNSFFVKTTPRKFL

FDGVEFCRDPVGVAQLVCQMVEDRKSATITKTPDNHALRFSMFNHKNVTHDGLYEVNTGI

RRLERLMRIERWNNARVLPHWKADKNGAPTTCQFINGTDGTAVAPFRQANDNLYIFSSDI

CRSVQVFYDEEINYNGIRGFRYSIRNNFLNEMPDCFCIDKIKGALTENSGCLYPGALDLT

DCLDAPVVATLPHFLNADPRYNVMVEGLNATPEKHNIFMDVEPYTGSPLRGGKRMQFNMF

LKQIEQIKLSENFKIPRLFPVLWVDEGLELNEEMTDLIKGDLTNVLTLVAVLQWSFVGIG

VALIFGMLLWYYLLTRKVKNSASVDPIYDVKG

>SNMP2_CLUMA_CG019584-PB

MTRGVERTIDHIPVVNVVKKMLDRYTPLQSILQTVENETPFFMPVINDQLHTIFGPTNSF

FVKTTPRKFLFDGVEFCRDPVGVAQLVCQMVEDRKSATITKTPDNHALRFSMFNHKNVTH

DGLYEVNTGIRRLERLMRIERWNNARVLPHWKADKNGAPTTCQFINGTDGTAVAPFRQAN

DNLYIFSSDICRSVQVFYDEEINYNGIRGFRYSIRNNFLNEMPDCFCIDKIKGALTENSG

CLYPGALDLTDCLDAPVVATLPHFLNADPRYNVMVEGLNATPEKHNIFMDVEPYTGSPLR

GGKRMQFNMFLKQIEQIKLSENFKIPRLFPVLWVDEGLELNEEMTDLIKGDLTNVLTLVA

VLQWSFVGIGVALIFGMLLWYYLLTRKVKNSASVDPIYDVKG
